# Cheek tooth morphology and ancient mitochondrial DNA of late Pleistocene horses from the western interior of North America: Implications for the taxonomy of North American Late Pleistocene *Equus*

**DOI:** 10.1371/journal.pone.0183045

**Published:** 2017-08-17

**Authors:** Christina I. Barrón-Ortiz, Antonia T. Rodrigues, Jessica M. Theodor, Brian P. Kooyman, Dongya Y. Yang, Camilla F. Speller

**Affiliations:** 1 Department of Quaternary Palaeontology, Royal Alberta Museum, Edmonton, Alberta, Canada; 2 Department of Biological Sciences, University of Calgary, Calgary, Alberta, Canada; 3 Ancient DNA Laboratory, Department of Archaeology, Simon Fraser University, Burnaby, British Columbia, Canada; 4 Department of Anthropology and Archaeology, University of Calgary, Calgary, Alberta, Canada; 5 BioArCh, Department of Archaeology, University of York, York, United Kindom; Kobenhavns Universitet Statens Naturhistoriske Museum, DENMARK

## Abstract

Horses were a dominant component of North American Pleistocene land mammal communities and their remains are well represented in the fossil record. Despite the abundant material available for study, there is still considerable disagreement over the number of species of *Equus* that inhabited the different regions of the continent and on their taxonomic nomenclature. In this study, we investigated cheek tooth morphology and ancient mtDNA of late Pleistocene *Equus* specimens from the Western Interior of North America, with the objective of clarifying the species that lived in this region prior to the end-Pleistocene extinction. Based on the morphological and molecular data analyzed, a caballine (*Equus ferus*) and a non-caballine (*E*. *conversidens*) species were identified from different localities across most of the Western Interior. A second non-caballine species (*E*. *cedralensis*) was recognized from southern localities based exclusively on the morphological analyses of the cheek teeth. Notably the separation into caballine and non-caballine species was observed in the Bayesian phylogenetic analysis of ancient mtDNA as well as in the geometric morphometric analyses of the upper and lower premolars. Teeth morphologically identified as *E*. *conversidens* that yielded ancient mtDNA fall within the New World stilt-legged clade recognized in previous studies and this is the name we apply to this group. Geographic variation in morphology in the caballine species is indicated by statistically different occlusal enamel patterns in the specimens from Bluefish Caves, Yukon Territory, relative to the specimens from the other geographic regions. Whether this represents ecomorphological variation and/or a certain degree of geographic and genetic isolation of these Arctic populations requires further study.

## 1. Introduction

Horses were a dominant component of North American Pleistocene land mammal communities and their remains are well represented in the fossil record [1–3]. Despite the abundant material available for study, there is still considerable disagreement over the number of species that inhabited the continent and on the taxonomic nomenclature. More than 40 species of *Equus* have been named from the Pleistocene of North America [4]. Several authors have attempted to revise the taxonomy of this group (e.g., [4–12]), but no consensus has been reached. The discrepancies in opinion regarding the taxonomy of North American *Equus* is the result of several factors including the use of different operational species concepts, the specimens included in the study, the choice of characters examined, and the specific methods used to study these characters, as exemplified by the studies by Winans [4, 10], Azzaroli [11, 12], and Weinstock et al. [13].

One of the first large-scale quantitative studies of the genus *Equus* in North America was undertaken by Winans [4, 10]. She conducted a multivariate analysis using linear measurements of cranial and metapodial remains. Her study sample consisted of equid specimens of Blancan to late Rancholabrean North American Land Mammal Ages (NALMA) largely from the Great Plains and the Western United Sates, and smaller samples from Florida, and Mexico. Winans’ approach was to identify morphological clusters in multivariate space, which she initially considered represented separate species [4], but later referred to them as species groups, indicating that some groups may include more than one species [10]. Five species groups are identified by Winans [10], three of which have temporal ranges that extend into the late Pleistocene: *Equus alaskae* (Hay), 1913 (small and stout-legged species group), *E*. *francisci* Hay, 1915 (small and stilt-legged species group), and *E*. *laurentius* Hay, 1913 (large and stout-legged species group). The other two species groups are *E*. *simplicidens* Cope, 1892 and *E*. *scotti* Gidley, 1900 [10].

Azzaroli [11, 12] identified ten taxa of *Equus* as being valid for North America during the Irvingtonian and Rancholabrean NALMAs (middle and late Pleistocene). The material he studied comes from different localities in the Great Plains and the Western United Sates, with additional specimens from Alaska, Florida, Canada, and Mexico. He based his taxonomic assignments on the recognition of what he considered diagnostic characters from a primarily qualitative study of the morphology of the skull, dentition, and limb bones as well as overall size. Nine of the species proposed to be valid by Azzaroli [12] have been found in late Pleistocene localities: *E*. *ferus* Boddaert, 1785, *E*. *niobrarensis* Hay, 1913, *E*. *lambei* Hay, 1917, *E*. *francisci* Hay, 1915, *E*. *fraternus* Leidy, 1860, *E*. *conversidens* Owen, 1869, *E*. *mexicanus* (Hibbard), 1955, *E*. *excelsus* Leidy, 1858, and *E*. *occidentalis* sensu Merriam, 1913. The other taxon identified by Azzaroli [12], *E*. *semiplicatus* Cope, 1892, is restricted to the early and middle Pleistocene.

More recently, Weinstock et al. [13] conducted an ancient mitochondrial DNA (mtDNA) study and a bi-variate analysis of metapodial dimensions of Eurasian, North American, and South American late Pleistocene equids. Most of the North American specimens studied by these authors come from sites located in the northwest region of the continent (Alaska, The Yukon Territory, Alberta, and Wyoming). Using maximum likelihood and Bayesian phylogenetic analysis, Weinstock et al. [13] concluded that only two lineages of equids, possibly each representing a distinct species, were present in this region of North America during the late Pleistocene. They do not assign species names to these two potential equid species and refer to them as the New World stilt-legged lineage and the stout-legged, caballine lineage.

The taxonomy of North American *Equus* has been in a state of flux for well over a century, and the studies summarized above clearly exemplify this. We believe that the most productive avenue to clarify the taxonomy and evolutionary relationships of middle and late Pleistocene equids is to conduct comprehensive morphological and molecular studies on the same set of specimens in order to contrast morphological and molecular variation. Here, we undertake such a study by conducting a morphometric analysis of the cheek teeth, using both linear and geometric morphometrics, and analizing ancient mtDNA obtained from a subsample of the teeth studied.

The study of the cheek teeth is important for two reasons. First, the use of the cheek teeth has been limited in the latest morphological revisions, even though they are well represented in the fossil record. Secondly, the dentition is one of the skeletal elements that best preserve ancient DNA and is less susceptible to contamination by exogenous DNA [14], allowing the opportunity for the recovery of molecular data for specimens from localities in southern North America. Furthermore, discrimination of *Equus* species using the cheek teeth is of particular relevance because teeth are archives of paleobiological and paleoclimatic information. Often techniques used to extract this information (e.g., stable isotope analysis) are destructive and are performed on isolated teeth, which are regularly identified only as *Equus* sp. (e.g., [15–19]), limiting the full potential of these studies. Refining the taxonomic assignment of isolated cheek teeth will allow for more in depth investigations into the paleobiology and extinction of Pleistocene North American equids.

**Fig 1:**
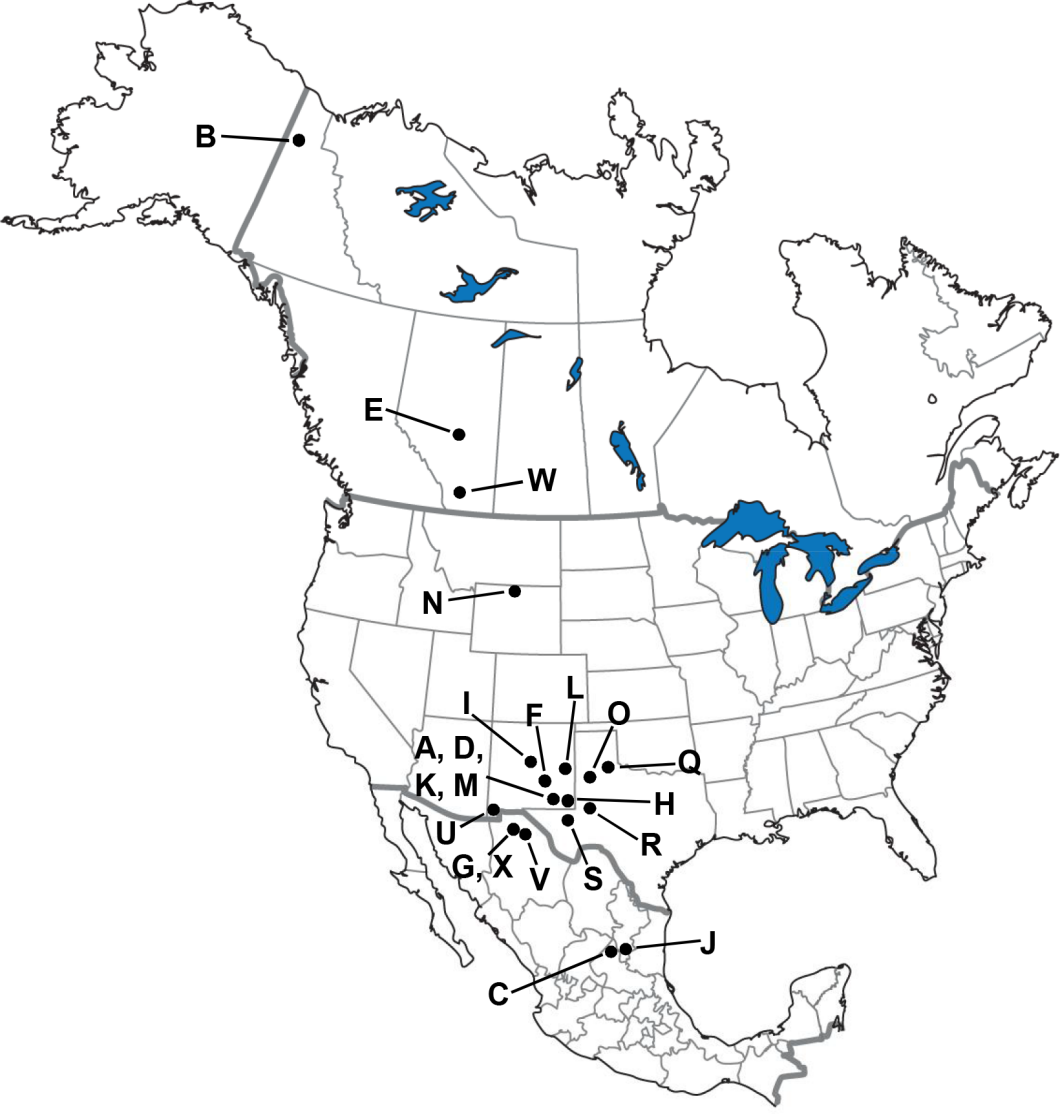
Geographic location of the fossil sites considered in the study. Northeastern Mexico: C = Cedral, J = San Josecito Cave; American Southwest: A = Algerita Blossom Cave, M = Big Manhole Cave, L = Blackwater Draw, K = Dark Canyon Cave, D = Dry Cave, X = El Barreal, F = Fresnal Canyon, G = Highway 45, Chihuahua, I = Isleta Cave No. 2, O = Lubbock Lake, H = Nash Draw, Q = Quitaque Creek, S = Salt Creek, R = Scharbauer Ranch, U = U-Bar Cave, V = Villa Ahumada; Wyoming: N = Natural Trap Cave; Alberta: E = Edmonton area gravel pits, W = Wally’s Beach site; Yukon Territory: B = Bluefish Caves.

This study concentrates on fossil material retrieved from five geographic regions approximately arranged in a north-south transect along the Western Interior of North America, from the High Arctic of the Yukon Territory, Canada, to northeastern Mexico (Fig 1). All of the specimens we examined are late Pleistocene in age, primarily from the mid- to late-Wisconsin glacial stage (approximately 50,000 to 10,000 radiocarbon years BP). Below, we briefly review some of the previous research conducted on horse remains from localities of the five geographic regions studied and discuss the species that have been identified.

### 1.1 Northeastern Mexico: San Josecito Cave and Cedral fossil sites

The fossil localities of San Josecito Cave (Nuevo León) and Cedral (San Luis Potosí) are two of the most studied late Pleistocene sites in northeastern Mexico. Stock [20, 21] considered all of the horse remains from San Josecito Cave to belong to a single species of *Equus*, which he thought was most similar to *Equus conversidens* Owen, 1869, but with sufficient morphological differences to be identified as a new subspecies: *E*. *conversidens leoni*. As pointed out by Dalquest [9] and Winans [4, 10], Stock [20, 21] did not select a type nor publish a formal description; thus, the name should be regarded as a *nomen nudum*. Moreover, Winans [4] proposed *E*. *conversidens* to be a *nomen dubium*, because she considered, in accordance with Hibbard [7], that the convergence of the cheek tooth rows toward the rostrum seen in the holotype (one of the main diagnostic characters for this species) was the result of a distorted restoration. Winans [10] assigned the specimens from San Josecito Cave to her species group *E*. *alaskae* (Hay), 1913. Contrary to Winans [4], Azzaroli [12] regarded *E*. *conversidens* as a valid species distinct from *E*. *niobrarensis alaskae* Hay, 1913 and identified the latter as a synonym of *E*. *ferus* Boddaert, 1785. He figured and described a partial skull from central Mexico, in which the two tooth rows converge toward the rostrum, suggesting that the holotype of *E*. *conversidens* was correctly mounted. In addition, Azzaroli [12] referred the fossil material from San Josecito Cave to *E*. *conversidens*, further stating that this species was closely related to South American horses, a relationship that has been suggested by other researchers (e.g., [8, 22]).

Three equid species have been recognized from the late Pleistocene deposits of Cedral, San Luis Potosí, Mexico, based on differences in size [23–27], metapodial proportions [24, 26], and features of the occlusal enamel pattern of the third and fourth upper premolars [25]. The large and medium-sized species have been tentatively identified as *E*. *mexicanus* (Hibbard), 1955 (originally described by Hibbard [7] as *E*. *(Hesperohippus) mexicanus*), and *E*. *conversidens*, respectively [23, 24, 26, 27]. The taxonomic identification of the smaller equid has been more problematic. Alberdi et al. [23] originally identified it as *Equus* sp. A, whereas Melgarejo-Damian and Montellano-Ballesteros [24] assigned it to *Equus tau* Owen, 1869. Recently, Alberdi et al. [27] have designated a new species, *Equus cedralensis*, for this material.

### 1.2 The American Southwest: New Mexico, western Texas and northern Chihuahua, Mexico

A number of important late Pleistocene fossil localities are known from New Mexico and western Texas, all of which have yielded large numbers of equid specimens, including Blackwater Draw Loc. 1, Dry Cave, Dark Canyon Cave, and U-Bar Cave in New Mexico as well as Scharbauer Ranch and Quitaque Creek in Texas. Blackwater Draw Loc. 1, New Mexico, is the type locality of the Clovis cultural complex and a large collection of bones as well as lithic artifacts and other cultural remains have been retrieved from this site [28]. The equid material from Blackwater Draw has been assigned to a variety of species. Stock and Bode [29] considered that only one species, *E*. *excelsus* Leidy, 1858, was represented in the material they studied. In contrast, Quinn [30] identified four taxa from this locality: *Asinus conversidens*, *Equus caballus caballus* Linnaeus, 1758, *E*. *caballus laurentius* Hay 1913, (originally described by Hay (1913) as *E*. *laurentius*), and *E*. *midlandensis* Quinn, 1957, a new species he named based on specimens from Scharbauer Ranch, Texas. Quinn [30] adhered to the proposal of dividing modern and fossil species of *Equus* into four genera, which consists of *Equus* for horses, *Asinus* for African asses and the domestic donkey, *Onager* for Asiatic asses, and *Hippotigris* for zebras. Lundelius [31], working with a larger sample from the Gray Sand unit of Blackwater Draw, agreed with Quinn [30] in identifying *A*. *conversidens*, but following a broader definition of the genus *Equus* he referred it to *E*. *conversidens*. In addition, Lundelius [31] reassigned the material identified by Quinn [30] as *E*. *caballus laurentius* to *E*. *niobrarensis* Hay 1913, whereas he reassigned the specimens identified as *E*. *midlandensis* and *E*. *caballus caballus* to *E*. *scotti* Gidley, 1900. A few years later, Harris and Porter [22] concluded that the specimens studied by Stock and Bode [29], Quinn [30], and Lundelus [31], with the exception of *E*. *conversidens*, appear to be assignable to *E*. *niobrarensis*. Recently, Harris [32] has revised his opinion and now considers *E*. *niobrarensis* a junior synonym of *E*. *scotti*.

In his study of fossil Equidae from Texas, Quinn [30] also examined, among other material, specimens from Scharbauer Ranch. Like Blackwater Draw Loc. 1, this locality has also yielded lithic artifacts and other cultural remains [33]. Quinn [30] identified some of the equid specimens he studied as *A*. *conversidens* and proposed a new species of large and stout-legged equid which he named *E*. *midlandensis*. This latter species is not considered to be valid by various authors. Lundelius [31] regarded *E*. *midlandensis* a synonym of *E*. *scotti*, Harris and Porter [22] proposed that it was synonymous with *E*. *niobrarensis*, whereas Winans [4] considered it a synonym of *E*. *mexicanus*, a species she thought was distinct from *E*. *scotti*. Winans [10] later proposed the name *E*. *laurentius* for the species group of *E*. *mexicanus*; however, it was recently shown that the holotype of *E*. *laurentius* belongs to a historic domestic horse and it is therefore a junior synonym of this species [34], a conclusion that had previously been expressed in the literature (e.g., [4, 6, 11, 12, 35]).

Dalquest [36] described an assemblage of fossils from a small tributary of Quitaque Creek, western Texas. Most of the equid remains collected were from a species of small horse, which Dalquest [36] identified as *E*. cf. *conversidens* based on similarities with specimens from the Valley of Mexico referred to *E*. *conversidens* by Hibbard [7]. There were also some remains of a larger horse slightly smaller than the average size of comparable elements identified as *E*. *scotti* from the Seymour formation of Knox County, Texas, which Dalquest [36] reported as *Equus* sp.

In one of the first studies that applied multivariate morphometrics to fossil equids, Harris and Porter [22] studied the equid remains from Dry Cave, southeastern New Mexico. They concluded that *E*. *conversidens* and *E*. *niobrarensis* were represented in the material they studied and also referred some specimens to *E*. *occidentalis* sensu Merriam, (1913), *E*. *scotti*, and a small zebrine species, which they called *E*. sp. A. Winans [10] studied specimens from Dry Cave and assigned them to the small, stout-legged species group of *E*. *alaskae* and the large, stout-legged species group of *E*. *laurentius*. Harris [32] has revised his interpretation of the equid remains from Dry Cave and currently recognizes *E*. *conversidens*, *E*. *scotti* (which he considers the senior synonym of *E*. *niobrarensis*), *E*. *occidentalis* (sensu Merriam, (1913); for the largest specimens in the fauna), *E*. sp. A (a small zebrine species), and a single partial upper tooth identified as *E*. *francisci*.

In addition to Dry Cave, there are several other cave sites from the American Southwest that have yielded equid remains. Two of these are U-Bar Cave, located in southwestern New Mexico, and Dark Canyon Cave, found south of Dry Cave, in southeastern New Mexico. Harris [37, 38] studied the fossil material collected from U-Bar Cave, separating it into mid-Wisconsin and late Wisconsin ages. He listed three equid species for the mid-Wisconsin of U-Bar Cave, namely *E*. *conversidens*, *E*. cf. *niobrarensis*, and *E*. cf. *occidentalis*, whereas for the late Wisconsin he considered that only *E*. *conversidens* and *E*. cf. *niobrarensis* were represented in the fauna [37]. Harris [32] maintains the same interpretation of the equid material from U-Bar Cave, with the exception that he considers *E*. *niobrarensis* to be a junior synonym of *E*. *scotti*. Regarding Dark Canyon Cave, Lundelius [22, 39] tentatively identified *E*. *conversidens* and *E*. *scotii* from this site, whereas Harris and Porter [22] referred to *E*. *conversidens* a small collection of equid remains from this locality housed at the University of Texas at El Paso. In his dissertation, Tebedge [40] described the fauna collected from excavations undertaken in the East Side Pocket of Dark Canyon Cave. He decided to identify the equid material as *Equus* sp. because of the confused nomenclature of Pleistocene equids [40].

The Vertebrate Paleobiology Collection of the University of Texas at El Paso houses specimens from different parts of Chihuahua, Mexico. Among these is a small collection of fossils from the ranch of Santa Barbara, located 9 km north of Villa Ahumada, northern Chihuahua [41]. In their report of this fossil locality, Comadurán et al. [41] identified the presence of *Mammuthus* sp. and *Equus* sp. Harris [32] examined the fossil material from this locality and identified the equid remains as *Equus francisci*.

### 1.3 Natural Trap Cave, Wyoming

The Natural Trap Cave fossil locality, Wyoming, has yielded thousands of vertebrate remains [42]. In a report of the excavations at Natural Trap Cave, Martin and Gilbert [43] mentioned the presence of three horse species for the equid material known at the time. They remarked that the most common species was a small, stilt-legged equid likely referrable to *Hemionus* [43], a group which has been treated as a genus or subgenus of *Equus* and which includes the extant Asiatic asses. Martin and Gilbert [43] indicated that the other two species were less abundant and that one of them is assignable to the subgenus *Amerhippus*. Winans [10] studied several specimens from Natural Trap Cave and assigned them to the species group of *E*. *alaskae*, which generally includes small, stout-legged horses. In a recent study using ancient mtDNA, Weinstock et al. [13] concluded that two clades were present at this locality, a caballine and a stilt-legged clade, each possibly representing a single species. The study by Weinstock et al. [13] further indicated that the stilt-legged clade is endemic to North America and that the presence of slender metapodials is, therefore, a convergent feature with extant Asiatic asses. In contrast, Eisenmann et al. [44] proposed that four equid species are represented in the material from Natural Trap Cave: a caballine, *E*. cf. *conversidens*, and a large and small *Amerhippus*, both with slender metapodials. According to Eisenmann et al. [44], the small *Amerhippus* is the most common species in the fauna.

### 1.4 Alberta, Canada: The Edmonton area gravel pits and Wally’s Beach site

The equid material from the Edmonton area gravel pits has not been described in detail. Burns and Young [45] listed two types of horses, which they referred to *Equus* cf. *conversidens* and *E*. cf. *niobrarensis*. Weinstock et al. [13] obtained ancient mtDNA from a large sample of specimens from the gravel pits around the Edmonton area. All of the specimens they studied were found to belong to the caballine clade, suggesting that only one species was represented in the sample they studied [13].

The archaeological-paleontological site of Wally’s Beach located in southern Alberta is remarkable in that it is the only known late Pleistocene horse and camel kill and butchering locality in North America [46–48]. Seven butchered horses were recovered associated with lithic artifacts [46]. McNeil [49] compared the equid material collected from Wally’s Beach to specimens from the Yukon Territory identified as *E*. *lambei*, as well as a skull from Papago Springs Cave, Arizona, identified by Skinner [50] as *E*. *conversidens*. McNeil [49] assigned the equid material from Wally’s Beach to *E*. *conversidens* and noted several differences between the Wally’s Beach sample and the sample of *E*. *lambei*, particularly in skull morphology and dentition.

### 1.5 The Yukon Territory: Bluefish Caves

The Bluefish Caves are located in northern Yukon Territory above the Arctic circle and have yielded, in addition to a large collection of vertebrate remains, some lithic artifacts and butchered bones, as well as other cultural evidence that extends from the late glacial to the LGM or possibly even earlier [51–53]. Burke and Cinq-Mars [54, 55] studied the horse remains from Bluefish Caves identified as *E*. *lambei* Hay, 1917. These authors documented the range of variation in cheek tooth morphology [54] and also constructed mortality profiles for each of the three caves [55]. *Equus lambei* has been identified as an onager [30], as a member of the genus *Asinus* [56, 57], and as a caballine equid [6, 58–61]. This species has also been considered a junior synonym of *E*. *ferus caballus* [4, 6] or *E*. *asinus* [4], it has also been assigned to the *E*. *alaskae* species group of Winans [10], and has been regarded as a possible subspecies of *E*. *niobrarensis* by Azzaroli [11, 12]. Burke and Cinq-Mars [54] concluded that *E*. *lambei* was a caballine horse, based on the morphology of the cheek tooth dentition. Weinstock et al. [13] successfully extracted, amplified, and sequenced, ancient mtDNA from one horse metatarsal from Bluefish Cave 3. Although the specimen was only identified as *Equus* sp., the sequence obtained by Weinstock et al. [13] placed it within the caballine group. Other late Pleistocene sites in Beringia have yielded fossil material of a horse with slender metapodials [62, 63], a feature that is present in extant hemione (Asiatic ass) species. However, molecular analysis of slender metapodials from the Yukon by Weinstock et al. [13] have placed this equid outside of the modern hemiones as a distinct species. These studies suggest that at least two species of *Equus* where present in Beringia during the late Pleistocene.

## 2. Materials and methods

All of the specimens studied are housed at one of the following institutions, with corresponding institutional acronyms indicated in parentheses: Canadian Museum of History (CMH; Bluefish Caves collections: MgVo-1, 2, and 3), Gatineau, Quebec, Canada; Quaternary Paleontology (P) and Archaeology collections (Wally’s Beach site; DhPg-8) of the Royal Alberta Museum (RAM), Edmonton, Alberta, Canada; Archeozoology Laboratory ‘M. en C. Ticul Álvarez Solórzano’ (DP), Instituto Nacional de Antropología e Historia (INAH), Mexico City, Mexico; Natural History Museum of Los Angeles County (LACM), Los Angeles, California, USA; University of Kansas (KU), Lawrence, Kansas, USA; University of Texas at El Paso (UTEP), El Paso, Texas, USA; and the Vertebrate Paleontology Laboratory, University of Texas at Austin (TMM), Austin, Texas, USA.

**Fig 2:**
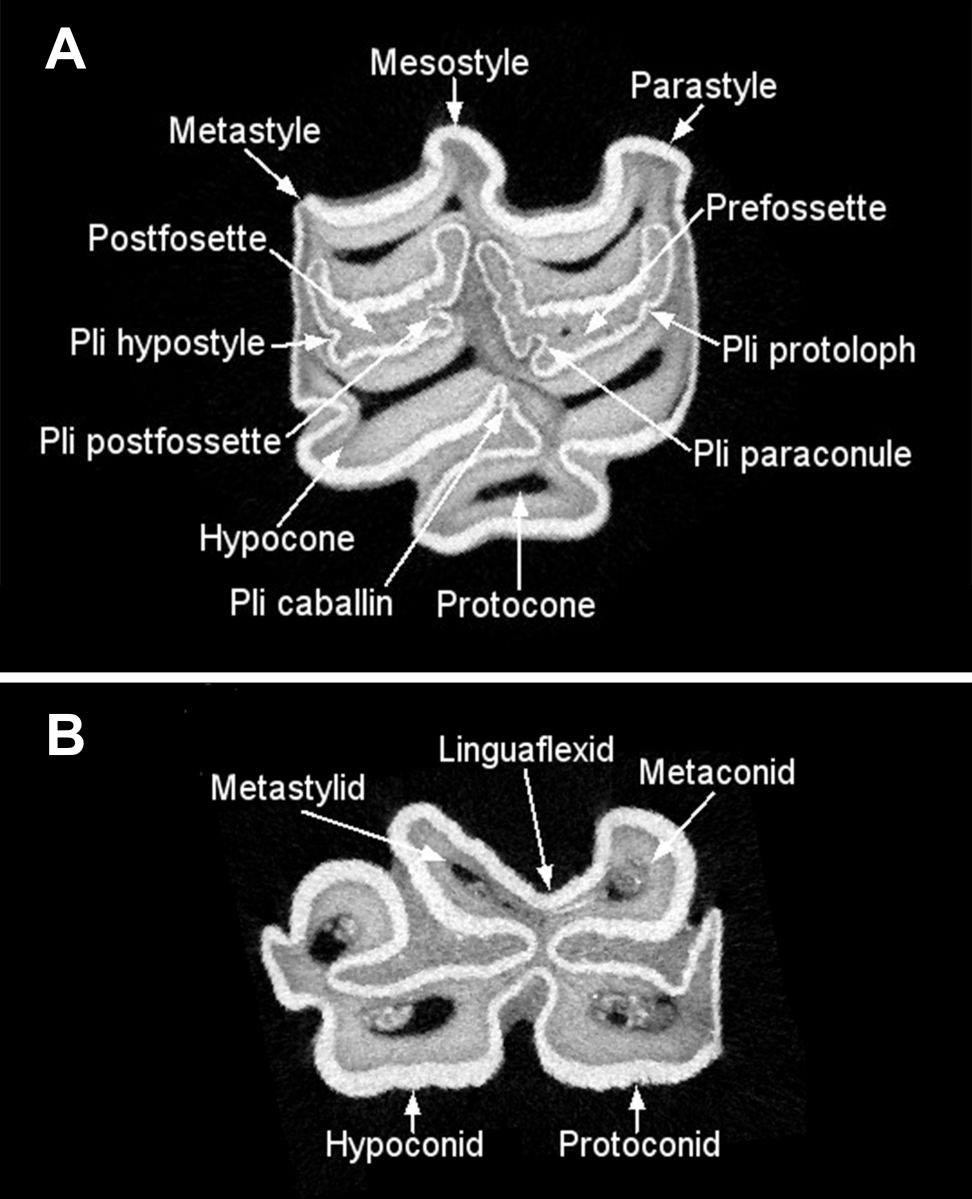
**Upper (A) and lower (B) fourth premolars showing the dental structures referred to in the text, following the dental nomenclature of Evander [64].** Computed tomography (CT-scan) images of LACM 192/156497 (A) and TMM 937–169 (B). The anterior side of both teeth is located to the right. The lingual side is located at the bottom in figure A and the top in figure B.

Throughout this study, we use the revised dental nomenclature proposed by Evander [64]. The primary structures for upper and lower cheek teeth referred to in the text are shown in Fig 2.

Before explaining the methodology for the morphometric and molecular analyses conducted here, we feel that it is important to include a note on morphological and phylogenetic species concepts and the approach we took in our study. Determination of species based on morphometric analyses is done under the morphological species concept, whereas determination of species based on phylogenetic analyses of molecular data makes use of the phylogenetic species concept. Under the morphological species concept, species are recognized based on morphological characters. It is assumed that species display a certain definable variability and are sufficiently distinct from other samples [65, 66]. The morphological species concept is ahistorical; that is, it does not consider ancestor-descendant relationships in the identification of species [67–69]. On the other hand, the phylogenetic species concept is historical and under the more common version of this species concept, a species is “a diagnosable cluster of individuals with which there is a parental pattern of ancestry and descent, beyond which there is not, and which exhibits a pattern of phylogenetic ancestry and descent among unit of like kind” (p. 92 in [70])]. In this study, we gave priority to the results obtained in the phylogenetic analyses when encountering discrepancies between these analyses and the morphometric studies. Nevertheless, the results of the two sets of analyses are mostly congruent with each other.

### 2.1 Linear morphometrics

#### 2.1.1 Measurements and sample size

**Fig 3:**
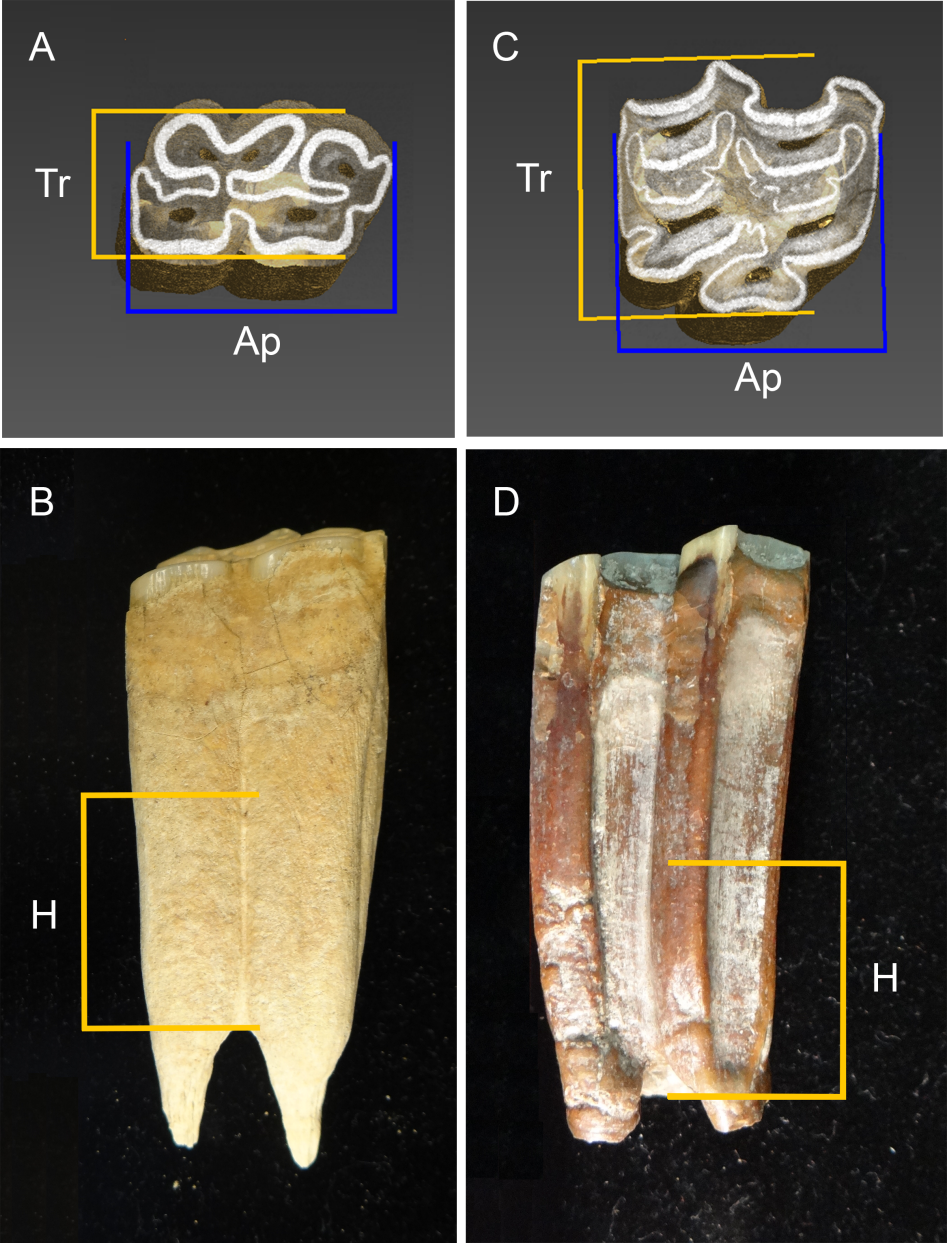
**Lower (A and B) and upper (C and D) third premolars showing the measurements collected for lower and upper cheek teeth.** Transverse width (Tr) and anteroposterior length (Ap) measurements are shown in figures A and C. These measurements were taken at a tooth crown height of 2 cm. In the lower teeth the measurements were taken 2 cm above from the bifurcation of the protoconid and hypoconid columns measured on the buccal side of the tooth (B). In the upper teeth the measurements were taken 2 cm above from where the mesostyle ends on the buccal side of the tooth (D). Figures A and B: left p3 (DhPg-8 3437.1). Figures C and D: right P3 (DP 3850).

The cheek tooth dentition of equids consists of three upper (P2, P3, and P4) and three lower (p2, p3, and p4) premolars, as well as three upper (M1, M2, and M3) and three lower (m1, m2, and m3) molars on each side of the dentition. We gathered linear measurements of the tooth crown dimensions of upper and lower cheek teeth using a Mitutoyo digital caliper with a measuring range of 0–150 mm, a resolution of 0.01 mm, and an accuracy of 0.003 mm. To account for measurement error, we took every measurement three times and used the mean of these measurements in all statistical analyses. All of the specimens were measured by the same researcher (CIB-O). The measurements collected are partially based on the methodology published by Eisenmann et al. [71]. For each cheek tooth studied, we measured the length and width at a crown height of 2 cm (Fig 3A and 3C). In the case of the lower teeth, these measurements were taken 2 cm above from the bifurcation of the protoconid and hypoconid columns measured on the buccal side of the tooth (Fig 3B), whereas in the upper teeth these measurements were taken 2 cm above from where the mesostyle ends on the buccal side of the tooth (Fig 3D). The occlusal dimensions of a tooth change as it wears down (e.g., [5, 72]) and taking measurements at a set tooth height compensates for this ontogenetic variation, especially in teeth with similar size and degree of hypsodonty. A potential drawback to this approach is that depending on the developmental stage of the tooth (and its state of preservation) sometimes a thin layer of cementum is present around the tooth crown where the measurements are taken. However, since this phenomenon was observed to occur in every sample studied, we do not expect it to be a source of systematic error.

A total of 1,454 cheek teeth were measured (738 upper and 716 lower teeth; Tables A–H in S1 File). Most of the specimens measured were isolated teeth, although there were some tooth series and some complete or partial dentaries and maxillaries. We determined the side and tooth position for every individual tooth using the criteria presented by Bode [73] and Eisenmann et al. [71]. The upper and lower third and fourth premolars (P3/p3 and P4/p4) are sometimes difficult to distinguish, as is the case for upper and lower first and second molars (M1/m1 and M2/m2). As a result, Eisenmann et al. [71] suggest combining upper P3 and P4 as well as lower p3 and p4 into a single category, respectively. The same suggestion applies to the upper M1 and M2 as well as the lower m1 and m2 teeth [71]. Consequently, we arranged the data into eight tooth categories: Upper P2, P3/P4, M1/M2, and M3; and lower p2, p3/p4, m1/m2, and m3. For cases in which different tooth positions of the same tooth category were associated (i.e., they belong to the same individual), for instance a p3 and a p4 of the same tooth series, we selected one of the two specimens at random and excluded the other from the analysis. For situations in which the left and right sides of the same tooth position were associated, for example a right P2 and a left P2, we measured both specimens, calculated the average, and used the average measurements in the statistical analysis. The final data sets for each of the five geographic regions and each tooth category studied are shown in Tables A–H in S1 File.

#### 2.1.2 Statistical analyses

We conducted a Principal Components Analysis (PCA) of the variance-covariance matrix for each of the eight tooth categories. Because of the possibility of a non-linear allometric relationship between the variables, we log-transformed the data prior to conducting the PCA. This transformation linearizes the data making it possible to use PCA and other statistical methods which assume linear relationships between variables [74]. For each tooth category, we first conducted a PCA for the five geographic regions combined, in order to place all of the specimens into the same multivariate space (i.e., morphospace). We then plotted the PC scores for the specimens from each geographic region separately (Figs A–J in S2 File). This facilitated the identification of different clusters in the morphospace, which were arranged along the first principal component (PC1). In order to statistically test for heterogeneity in the data that would indicate the presence of more than one population, we conducted a Shapiro-Wilk test for normal distribution for the PC1 scores. The null hypothesis is that the observations are drawn at random from a single population with a normal distribution. All statistical tests were conducted in PAST 2.17 [75] and STATISTICA v. 9 [76] software packages. The significance level for all tests was set to a p-value of 0.05.

### 2.2 Geometric morphometrics of the occlusal enamel pattern

The occlusal enamel pattern of equids is complex and has been used to varying degrees in the taxonomy of these ungulates. The occlusal surface and dimensions of hypsodont equid cheek teeth change with age as the teeth wear down (e.g., [5, 72, 77, 78]). This large ontogenetic variation has brought into question the utility of the cheek teeth in the determination of equid species. However, when comparing specimens at similar stages of wear, the enamel pattern can be taxonomically informative [25, 79]. In this study, we examined teeth with a tooth height representing 30–40% of the maximum crown height. This approximately corresponds to a crown height equivalent to the width of the tooth (or the length of the tooth for the lower teeth) measured at a tooth height of 2 cm as indicated above in the section on linear morphometrics.

#### 2.2.1 Image acquisition

**Fig 4:**
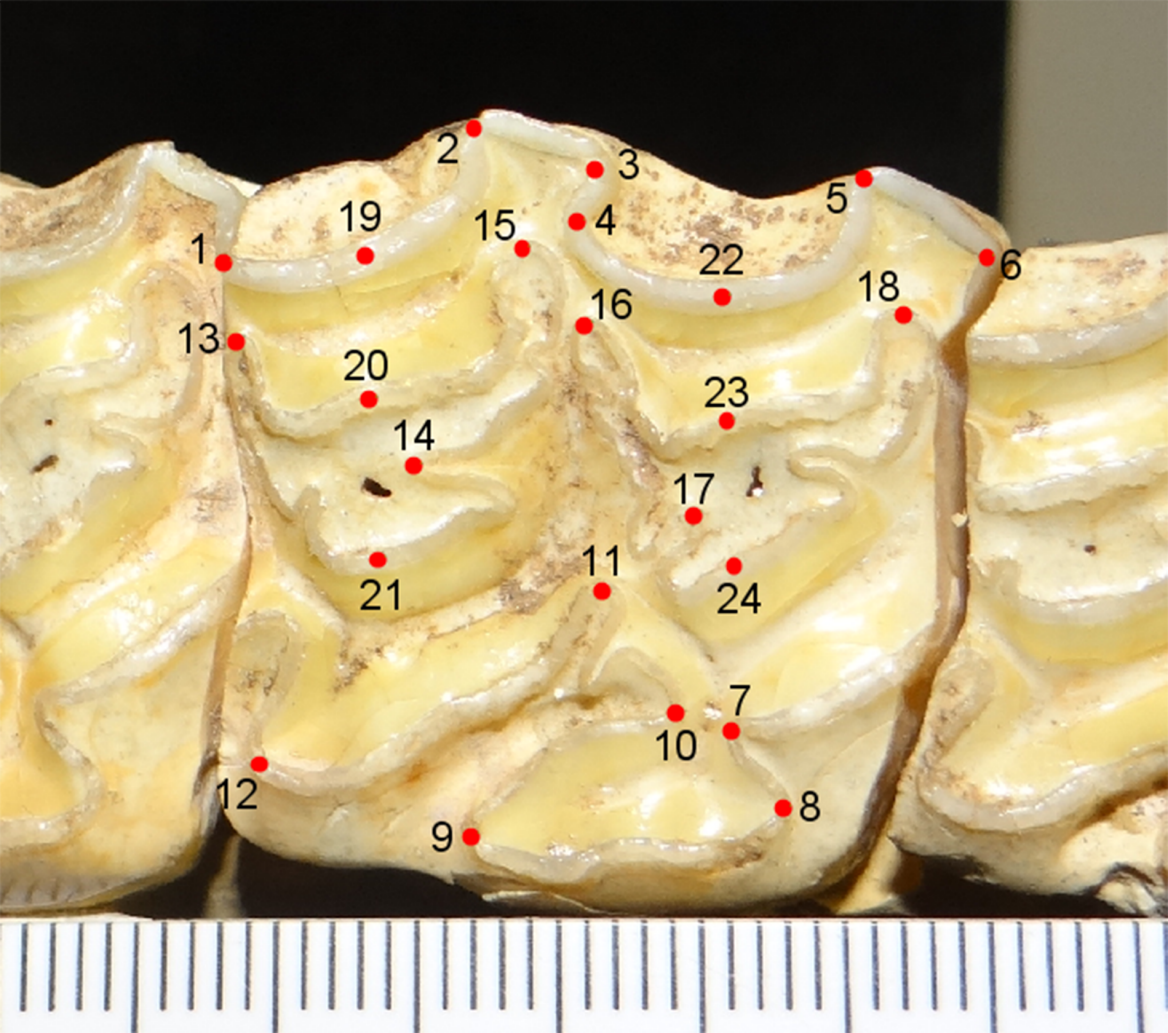
Occlusal surface of a P3 (LACM 192/18109) showing the 24 landmarks used in the analysis. Refer to the main text and Table C in S2 File for a description of the landmarks and details about how they were digitized.

**Fig 5:**
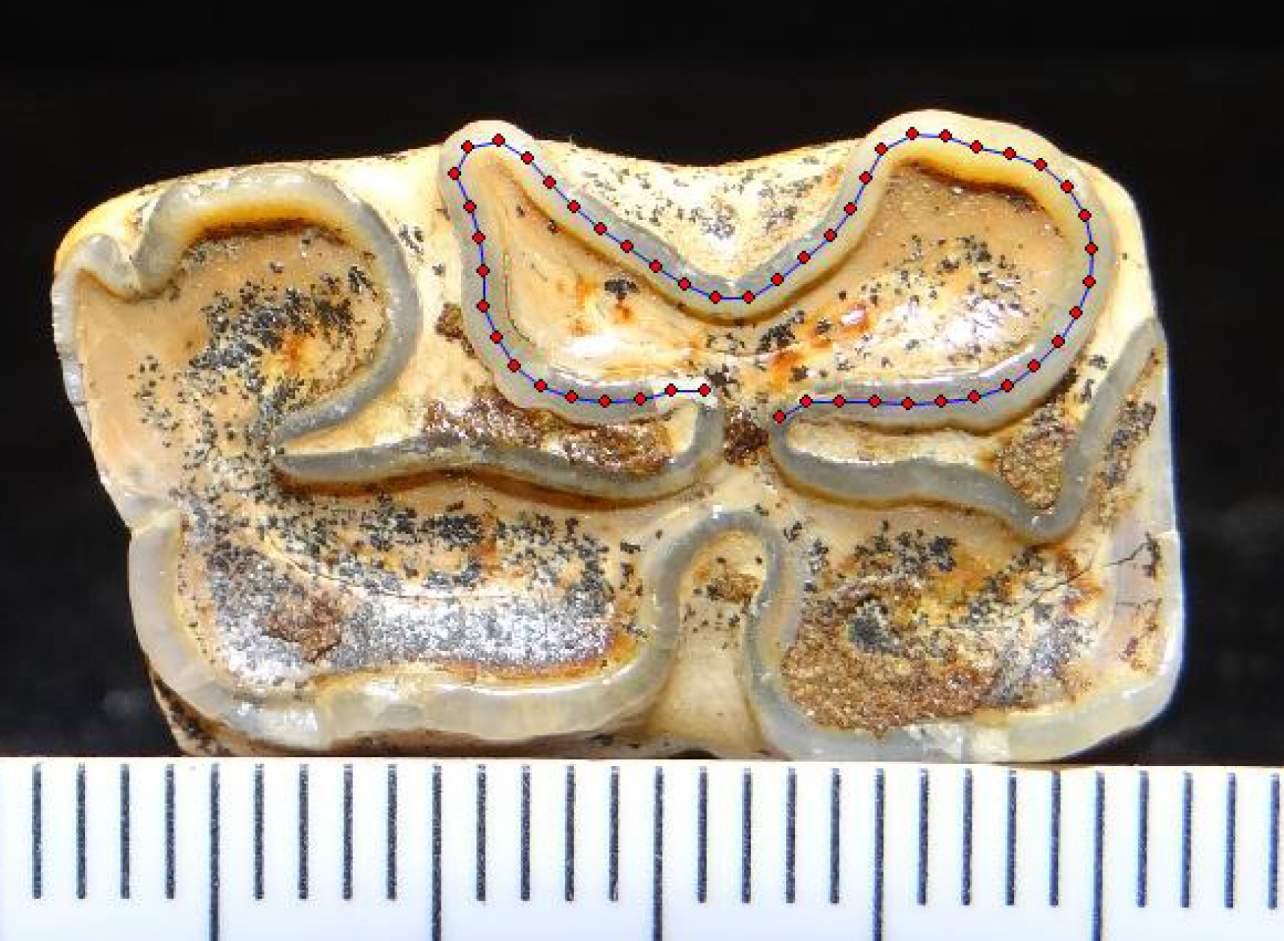
Digitized double knot (metaconid, linguaflexid, and metastylid) of a lower p4 (KU 50629) showing the 50 landmarks used in the analysis. Refer to the main text for details about how the landmarks were digitized.

We photographed specimens showing the selected stage of wear using a SONY Cyber-shot DSC-H9 digital camera. When taking the photograph, the occlusal surface of the tooth was oriented perpendicular to the camera lens. In addition, we placed a scale bar oriented parallel to the occlusal surface on the lingual side of the tooth for the upper teeth (Fig 4) and on the buccal side for the lower teeth (Fig 5).

Typically, fossil assemblages present teeth with varying degrees of wear; therefore, restricting the analysis to teeth with equivalent stages of wear reduces the effective sample size. In order to increase the sample size available for study we relied on X-ray Computed Tomography to digitally section specimens at the selected stage of wear. Due to limitations in CT-scanning time, we did not scan all of the tooth positions, but rather concentrated on the third and fourth premolars, as these tooth positions had proven to be taxonomically useful in a previous study of the occlusal enamel pattern [79]. As a result, our analysis focused solely on the upper P3/P4 and lower p3/p4 tooth categories.

In total, 139 specimens were CT-scanned, 64 upper P3/P4 and 75 lower p3/p4. All of the specimens were scanned using a SkyScan 1173 high-resolution micro-CT scanner at the Department of Comparative Biology and Experimental Medicine, University of Calgary. Three-dimensional (3-D) surface models of the teeth were created in AMIRA 5.3.3 and digitally sectioned at the selected tooth height. The specimens were not sectioned perpendicular to the long axis of the tooth, but rather the cutting plane was aligned with the occlusal surface of the tooth. Thus, the cutting plane was inclined lingually and mesially to varying degrees for the upper teeth and buccally and (generally) mesially on the lower teeth. The sectioned 3-D models were then oriented with the enamel pattern perpendicular to the screen and an image along with a scale bar was obtained.

In cases where there were images of associated specimens, for example left and right P3 of the same individual, we chose one of the two specimens at random for digitization. We also reflected all of the left teeth in the data set in order to have all of the specimens in the same orientation. We then renamed every image with a four digit identifier generated at random, with the objective of mixing the sample of images and removing the identity of each image to minimize any biases during digitization. The final data sets (including both photographed and CT-scanned specimens) consisted of 144 upper P3/P4 teeth (Table A in S2 File and data set in S3 File) and 128 lower p3/p4 teeth (Table B in S2 File and data set in S4 File).

#### 2.2.2 Upper cheek teeth landmark acquisition

We used the computer software tpsDig 2.16 [80] to digitize 24 landmarks on the images, including both photographs and images of the sectioned 3-D surface models, of the upper P3/P4 teeth. The landmarks used in this analysis are presented in Fig 4 and are listed in Table C in S2 File; they are based on a study reported by Barrón-Ortiz and Theodor [25]. The first 18 landmarks are considered type II landmarks under Bookstein’s [81, 82] classification of landmarks. Type II landmarks are points located at local maxima and minima of curvature. The remaining six landmarks are considered type III landmarks. Type III landmarks are defined by their relative position to other landmarks [81, 82]. We used type III landmarks to obtain a better characterization of the fossettes and the buccal enamel band of the tooth. We had initially placed a type II landmark on the pli hypostyle of the postfossette and on the pli protoloph of the prefossette, but decided to exclude these landmarks because these plications are not present in all of the specimens imaged. The pli hypostyle is absent from 18 specimens and the pli protoloph is absent from four teeth. All of the specimen images were processed and digitized by the same researcher (CIB-O).

#### 2.2.3 Lower cheek teeth landmark acquisition

For the lower cheek teeth, we focused on what some researchers call the double-knot (e.g., [71]), which consist of the metaconid, linguaflexid, and metastylid (Fig 2). The linguaflexid, in particular, has been considered taxonomically important by different researchers (e.g., [8, 83–86]). These researchers indicate that horses, including the Mongolian wild horse (E. ferus przewalskii), tend to have a deep U-shaped linguaflexid, whereas zebrines have a V-shaped linguaflexid, and hemiones have a shallow V- or U-shaped linguaflexid. One methodological complication with this categorization of the linguaflexid is that it is subjective, that is, whether a linguaflexid is categorized as U-shaped or V-shaped depends on the judgment of the researcher [84]. A further potential complication is that this character may be variable within the same species; at least this has been reported in populations of the extant hemione Equus kiang, in which northern populations tend to have a U-shaped linguaflexid, whereas southern populations tend to present a V-shaped linguaflexid [87]. The first complication can be addressed with the use of outline-based geometric morphometrics. This technique allows for the characterization of outlines or curves in a more objective manner. Regarding the second complication, additional studies are needed to better assess the morphological plasticity of this trait.

The outline of the metaconid-linguaflexid-metastylid complex of each tooth was digitized to obtain 50 evenly-spaced landmarks using the computer software tpsDig 2.16 [80] (Fig 5). The first landmark was placed on the distal point of the bucco-distal margin of the metaconid and the last landmark was placed on the mesial point of the bucco-mesial margin of the metastylid (Fig 5). The first and last landmarks are type II landmarks according to Bookstein’s [81, 82] classification of landmarks. The remaining 48 landmarks represent semilandmarks. All of the specimen images were processed and digitized by the same researcher (CIB-O).

#### 2.2.4 Statistical analyses

The goal of the geometric morphometric analysis was to determine whether the groups that we identified in the analysis of the linear measurements, which were primarily based on differences in size, statistically differed in shape for both the upper and the lower premolars. Shape refers to the geometric features of an object after accounting for differences in size, position, and orientation [88]. To this end, we organized the landmark data by size group (according to groups identified in the linear morphometric analysis) and geographic region. The groups considered in the analysis are: large, medium, and small specimens from northeastern Mexico; large, medium, and small specimens from the American Southwest; large and medium specimens from Natural Trap Cave, Wyoming; large and medium specimens from Alberta; and the specimens from Bluefish Caves, Yukon Territory.

For both the upper and the lower teeth, we superimposed the configuration of landmarks using the generalized least squares Procrustes superimposition algorithm in MorphoJ 1.05f [89]. We then performed a pooled within subgroups multivariate regression of log centroid size on the Procrustes residuals to test for allometry; covariation between size and shape (e.g., [90–93]). As will be seen in the results, the regression for both the upper and lower premolars yielded a statistically significant relationship. We used the regression residuals to control for the variation of shape due to size and conducted a Canonical Variate Analysis (CVA). To test for significant differences between groups, we carried out pair-wise permutation tests, using10,000 permutation rounds, for the Procrustes distances among groups.

### 2.3 Ancient mtDNA analysis

#### 2.3.1 Samples

We sampled 50 late Pleistocene equid teeth from 12 North American localities for ancient mtDNA analysis (Table D in S2 File). These specimens were included in the linear and/or geometric morphometric analyses described above. For each tooth we obtained a fragment of approximately 10 mm in length from the tip of the root in order to avoid damaging the tooth crown. The samples were processed at the Ancient DNA Laboratory of the Department of Anthropology and Archaeology, University of Calgary. Repeat extractions were conducted for five specimens and six other specimens were independently replicated at the Ancient DNA Laboratory of the Department of Archaeology, Simon Fraser University, following the same protocols (Table D in S2 File).

#### 2.3.2 DNA extraction, amplification, and sequencing

The ancient DNA laboratories at the University of Calgary and Simon Fraser University were designed exclusively for ancient DNA (aDNA) analyses and no modern samples have ever been processed in either laboratory. The laboratories are equipped with UV filtered ventilation and positive airflow, as well as UV sources for decontamination; all equipment in the laboratory is dedicated for aDNA use. Strict contamination protocols are followed including: 1) the use of protective clothing such as Tyvex suits, masks, and disposable gloves; 2) separation of the aDNA lab into bone preparation, DNA extraction, and PCR set-up rooms, with dedicated equipment for each room; 3) Separation of pre- and post-PCR workspaces; 4) the inclusion of multiple blank DNA extractions (one for every six to seven samples processed) and negative PCR controls.

Approximately 0.3–1.0 g of sample were subjected to chemical and UV decontamination: samples were immersed in 6% sodium hypochlorite for 7 minutes, rinsed twice in ultra-pure water, and UV irradiated in a crosslinker for 30 minutes on two sides. Following decontamination, the samples were crushed into powder and incubated overnight at 50 ^o^C in 5 ml of lysis solution (0.5 M EDTA pH 8.0, 0.5% SDS, and 0.5 mg/mL proteinase K). We used a modified silica-spin column technique [94, 95] to extract DNA from the decontaminated tooth samples. For each sample, approximately 200 μl of DNA extract were obtained in two separate elutions of 100 μl each.

Eight overlapping primer sets were designed to amplify a 621 bp fragment of the hypervariable region I (HVR I) of equid mitochondrial control region (Table E in S2 File), spanning positions 15,443–16,063 of the *Equus ferus caballus* mtDNA genome (Genbank accession: JN398377). We conducted PCR reactions using an Eppendorf Mastercycler® in a 30 μl reaction volume containing 50 mmol/L KCl, 10 mmol/L Tris-HCl, 2.5 mmol/L MgCl_2_, 0.2mmol/L dNTP, 1.0 mg/mL BSA, 0.3 μmol/L each primer, 3.0–4.0 μl DNA sample, and 2 U Ampli*Taq* Gold LD (Life Technologies Corporation, Carlsbad, California, USA). PCR started with an initial 12 min denaturation period at 95 ^o^C, followed by 60 cycles at 95 ^o^C denaturation for 30 s, 50–52 ^o^C annealing for 30 s, and 72 ^o^C extension for 40 s. We included blank extracts and negative controls in each of the PCR sets. PCR products were sequenced using forward and reverse primers at Eurofins MWG Operon, Inc., Huntsville, Alabama, USA. For all of the samples that yielded DNA we attempted repeat amplifications and sequencing and for five specimens (EQ29, EQ39, EQ43, EQ50, and EQ53) we conducted repeat extractions to ensure the reproducibility of the results and to detect any base pair misincorporations due to DNA damage. Six specimens were independently replicated at Simon Fraser University (EQ1, EQ2, EQ9, EQ30, EQ43, and EQ51).

#### 2.3.3 Data analysis

Contigs of the obtained DNA sequences were produced using ChromasPro software (http://technelysium.com.au/). The aligned DNA fragments were edited and truncated to remove primer sequences and to make them comparable with previously published equid reference sequences from GenBank.

We compiled DNA sequences of the mitochondrial control region of extant and extinct equids (Table F in S2 File) from GenBank, including sequences from the modern horse haplogroups identified by Achilli et al. [96], ancient horse sequences obtained by Weinstock et al. [13], sequences of stilt-legged horses reported by Vilstrup et al. [97], and sequences of specimens identified as *Equus (Amerhippus) neogeus* obtained by Orlando et al. [98]. We also included in the data set the mitochondrial control region of the fossil specimens from Thistle Creek, Yukon, and Taymyr peninsula, Siberia, reported by Orlando et al. [99]. We used sequences of the domestic and African donkeys as outgroups (Table F in S2 File). The sequences from the literature and the ones obtained in this study were arranged into one data set consisting of 125 sequences of a 588 bp fragment of the hypervariable region I (HVR I).

We aligned the sequences by way of a ClustalW Multiple alignment in BioEdit 7.0.5.3 [100]. Subsequently, we used MrModeltest 2.3 [101] in PAUP 4.0 Beta Version10 [102] to determine the best nucleotide substitution model. This analysis identified the general time reversible model with gamma-distributed rate variation across sites and a proportion of invariable sites (GTR+G+I) as the best model for the data set. We then conducted a Bayesian phylogenetic analysis integrating Markov chain Monte Carlo algorithms in MrBayes 3.2 [103]. The posterior probability distribution of trees was approximated by drawing a sample every 1,000 steps over 20,000,000 generations, after discarding a burn-in of 1,000,000 generations.

## 3. Results

### 3.1 Linear morphometrics

**Fig 6:**
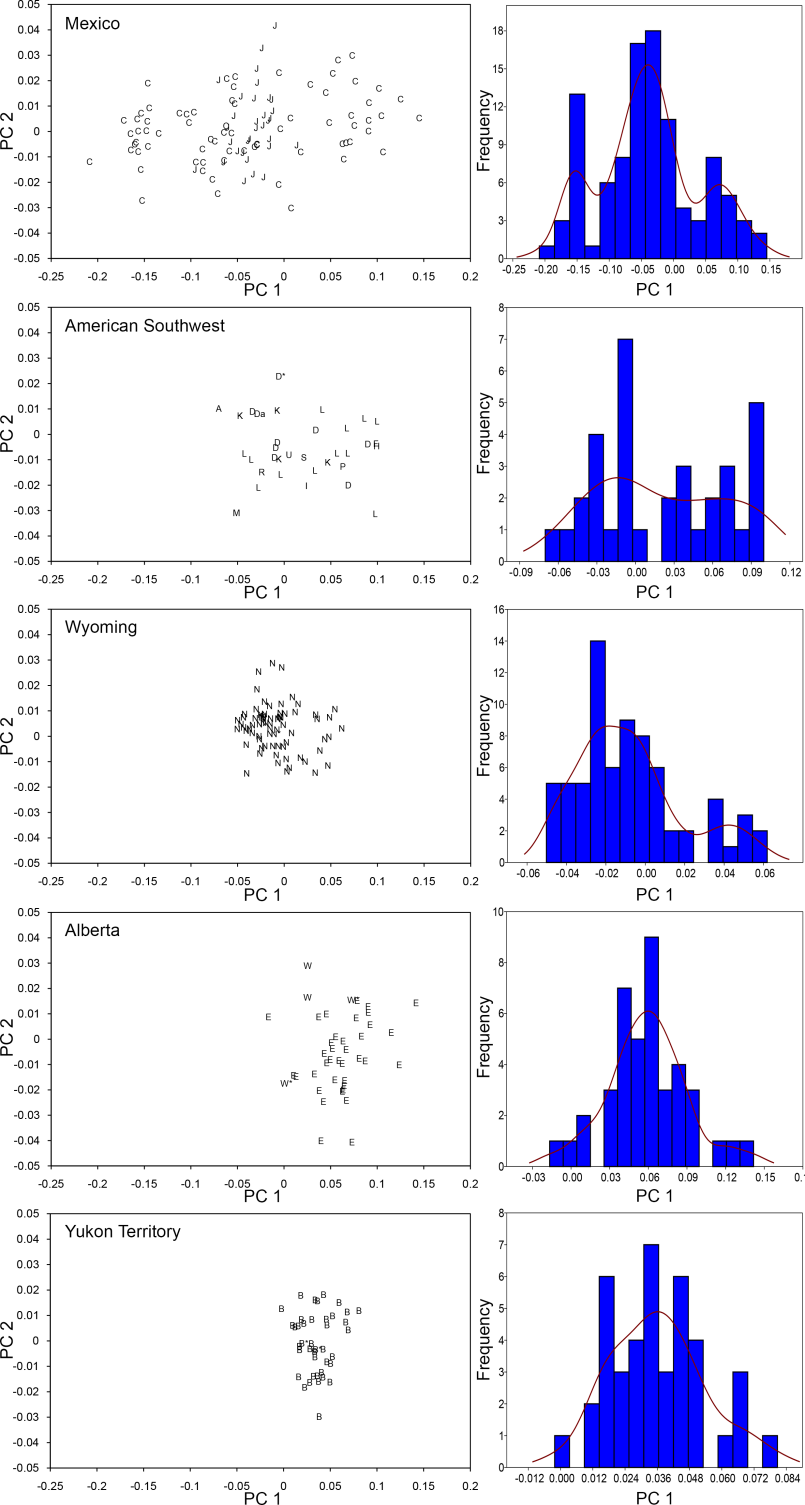
Principal component plots and histograms of PC 1 scores resulting from PCA of the linear measurements of upper M1/M2 teeth. The specimens come from five geographic regions of the Western Interior of North America: Mexico (C, Cedral; J, San Josecito Cave), the American Southwest (A, Algerita Blossom Cave; D, Dry Cave; F, Fresnal Canyon; K, Dark Canyon Cave; H, Nash Draw; I, Isleta Cave No. 2; L, Blackwater Draw; M, Big Manhole Cave; P, Imperial; R, Scharbauer Ranch; S, Salt Creek; U, U-Bar Cave); Wyoming (N, Natural Trap Cave); Alberta (E, Edmonton area gravel pits; W, Wally’s Beach), and the Yukon Territory (B, Bluefish Caves). A lower case “a” beside the specimen symbol indicates a tooth that yielded ancient mtDNA (EQ16 from Dry Cave). An asterisk (*) beside the specimen symbol denotes a tooth associated (i.e., it belongs to the same individual) with a specimen from which ancient mtDNA was obtained (teeth associated with EQ3 from Dry Cave, EQ9 from Natural Trap Cave, EQ29 and EQ43 from Wally’s Beach, EQ44 and EQ47 from Bluefish Caves). The dark line in the histograms corresponds to the kernel density estimation. Table C in S1 File lists all of the specimens included in this analysis.

In all analyses for both the upper and the lower cheek teeth, the first principal component (PC 1) accounted for over 87% of the variation in the data (Tables 1 and 2). The factor loadings indicate that this component reflects variation in size, with larger specimens showing more positive scores (Tables 1 and 2). Plotting the PC scores by geographic region for each tooth category reveals the presence of one to three size groups (Fig 6; Figs A–J in S2 File). These size differences do not correspond to sexual dimorphism as extant equid species and monospecific quarry samples of fossil species do not show sexual dimorphism in the cheek tooth dimensions investigated here [104].

**Table 1:** Eigenvalues, percentage variance, and factor loadings for the principal components resulting from PCA of the linear measurements of the upper teeth (Ap = anteroposterior length; Tr = transverse width), taken at a crown height of 2 cm.

	Upper P2		Upper P3/P4		Upper M1/M2		Upper M3	
	PC1	PC2	PC1	PC2	PC1	PC2	PC1	PC2
Eigenvalue	0.0031	0.0003	0.0044	0.0002	0.0041	0.0002	0.0051	0.0004
% variance	92.49	7.51	94.62	5.38	96.18	3.82	93.04	6.96
Factor loadings								
Ap	0.7773	-0.6291	0.7680	-0.6405	0.7401	-0.6725	0.7905	-0.6125
Tr	0.6291	0.7773	0.6405	0.7680	0.6725	0.7401	0.6125	0.7905

**Table 2:** Eigenvalues, percentage variance, and factor loadings for the principal components resulting from PCA of the linear measurements of the lower teeth (Ap = anteroposterior length; Tr = transverse width), taken at a crown height of 2 cm.

	Lower p2		Lower p3/p4		Lower m1/m2		Lower m3	
	PC1	PC2	PC1	PC2	PC1	PC2	PC1	PC2
Eigenvalue	0.0042	0.0006	0.0037	0.0004	0.0043	0.0005	0.0055	0.0005
% variance	87.34	12.66	89.81	10.19	90.01	9.99	91.90	8.10
Factor loadings								
Ap	0.6873	0.7264	0.7036	0.7105	0.6411	0.7675	0.7230	-0.6909
Tr	0.7264	-0.6873	0.7105	-0.7036	0.7675	-0.6411	0.6909	0.7230

The upper and lower cheek teeth from northeastern Mexico tend to plot into three size groups: large, medium, and small (Fig 6; Figs A and B in S2 File). Moreover, the distribution of the specimens along PC 1 (Fig 6; Figs K and L in S2 File) statistically departs from normality in all tooth categories, except M3 and p2 (Table 3).

**Table 3:** Results of Shapiro-Wilk test for normal distribution of principal component 1 scores for each tooth category and geographic region studied.

Tooth	Mexico			American Southwest			Wyoming			Alberta			Yukon Territory		
	n	Shapiro-Wilk W	*p*-value	n	Shapiro-Wilk W	*p*-value	n	Shapiro-Wilk W	*p*-value	n	Shapiro-Wilk W	*p*-value	n	Shapiro-Wilk W	*p*-value
P2	33	0.931	**0.038**	10	0.915	0.319	27	0.886	**0.006**	5	0.907	0.448	23	0.964	0.550
P3/P4	71	0.933	**0.001**	26	0.916	**0.036**	66	0.953	**0.014**	26	0.972	0.663	45	0.982	0.682
M1/M2	103	0.972	**0.026**	34	0.942	0.069	72	0.939	**0.002**	41	0.984	0.804	41	0.982	0.747
M3	37	0.950	0.094	12	0.932	0.404	35	0.904	**0.005**	11	0.952	0.675	20	0.927	0.135
p2	44	0.951	0.061	14	0.951	0.577	25	0.949	0.238	9	0.819	**0.034**	21	0.913	0.063
p3/p4	77	0.961	**0.018**	34	0.888	**0.002**	46	0.975	0.415	20	0.914	0.075	30	0.968	0.488
m1/m2	134	0.971	**0.006**	56	0.965	0.108	56	0.953	**0.030**	22	0.969	0.678	28	0.986	0.961
m3	33	0.932	**0.041**	11	0.883	0.112	32	0.944	0.097	5	0.852	0.202	19	0.908	0.067

n = sample size. Statistically significant *p*-values are shown in bold.

The specimens from the American Southwest tend to plot into large and medium size clusters, except for the p3/p4 tooth category where there are three small-sized specimens that plot in the same region of the morphospace as the small-sized specimens from northeastern Mexico (Fig 6; Figs C and D in S2 File). Sample sizes for this geographic region are small in four of the eight tooth categories, namely P2, M3, p2, and m3 (Table 3). As a result, greater weight was given to the remaining tooth categories in the interpretation of the Shapiro-Wilk test of normality. The distribution of PC1 scores for P3/P4 and p3/p4 cheek teeth is statistically different from the expected normal distribution, whereas normality is not rejected for the M1/M2 and m1/m2 tooth categories; although, the p-value for the M1/M2 category is marginally greater than 0.05 (Table 3; Fig 6; Figs M and N in S2 File).

The vast majority of the specimens from Natural Trap Cave plot into one cluster that falls in the same region of the morphospace as the medium size cluster from northeastern Mexico and the American Southwest (Fig 6; Figs E and F in S2 File). There are, however, a few specimens that are of larger size, producing a right-skewed distribution of specimens along PC1 in all of the tooth categories except p2 and p3/p4 (Fig 6; Figs O and P in S2 File). Accordingly, the Shapiro-Wilk test is not significant for these two tooth categories (Table 3). The test is also not significant for the m3 tooth category. Significant departures from normality are detected for the remaining five tooth categories (Table 3).

The specimens from Alberta tend to plot on the right side of the graph in the same region of the morphospace as the large specimens from the American Southwest and northeastern Mexico (Fig 6; Figs G and H in S2 File). However, this does not apply to all of the tooth positions, and there are four specimens (one p2, two p3/p4, and one m1) that are smaller in size and that fall in the same region of the morphospace as the medium-sized specimens from Natural Trap Cave, the American Southwest, and northeastern Mexico. The sample size in four of the eight tooth categories (P2, M3, p2, and m3) is small and, therefore, greater weight was given to the interpretation of the Shapiro-Wilk test for the other tooth categories. Normality cannot be rejected for the distribution of specimens along PC1 for the P3/P4, M1/M2, and m1/m2 tooth categories, and the test is marginally not significant for the p3/p4 tooth category (Table 3; Fig 6; Figs Q and R in S2 File).

The specimens from Bluefish Caves form one cluster in all tooth categories (Fig 6; Figs I and J in S2 File). The specimens from this locality show a size range that is intermediate to that of the large-sized specimens from Alberta, the American Southwest, and northeastern Mexico and the medium-sized specimens from Alberta, Natural Trap Cave, the American Southwest, and northeastern Mexico. The Shapiro-Wilk test is not significant for any of the tooth categories, although it is only marginally not significant for the p2 and m3 tooth categories (Table 3; Fig 6; Figs S and T in S2 File).

### 3.2 Geometric morphometrics of the enamel pattern of upper premolars

There is a statistically significant relationship between shape (as defined by the Procrustes coordinates) and log centroid size (*p*-value < 0.0001). The regression on centroid size accounts for 6.045% of the total shape variation. Thus, it was necessary to standardize the data by computing the residuals from the regression to remove the shape variation due to allometry. The residuals were then used in further statistical analyses.

**Fig 7:**
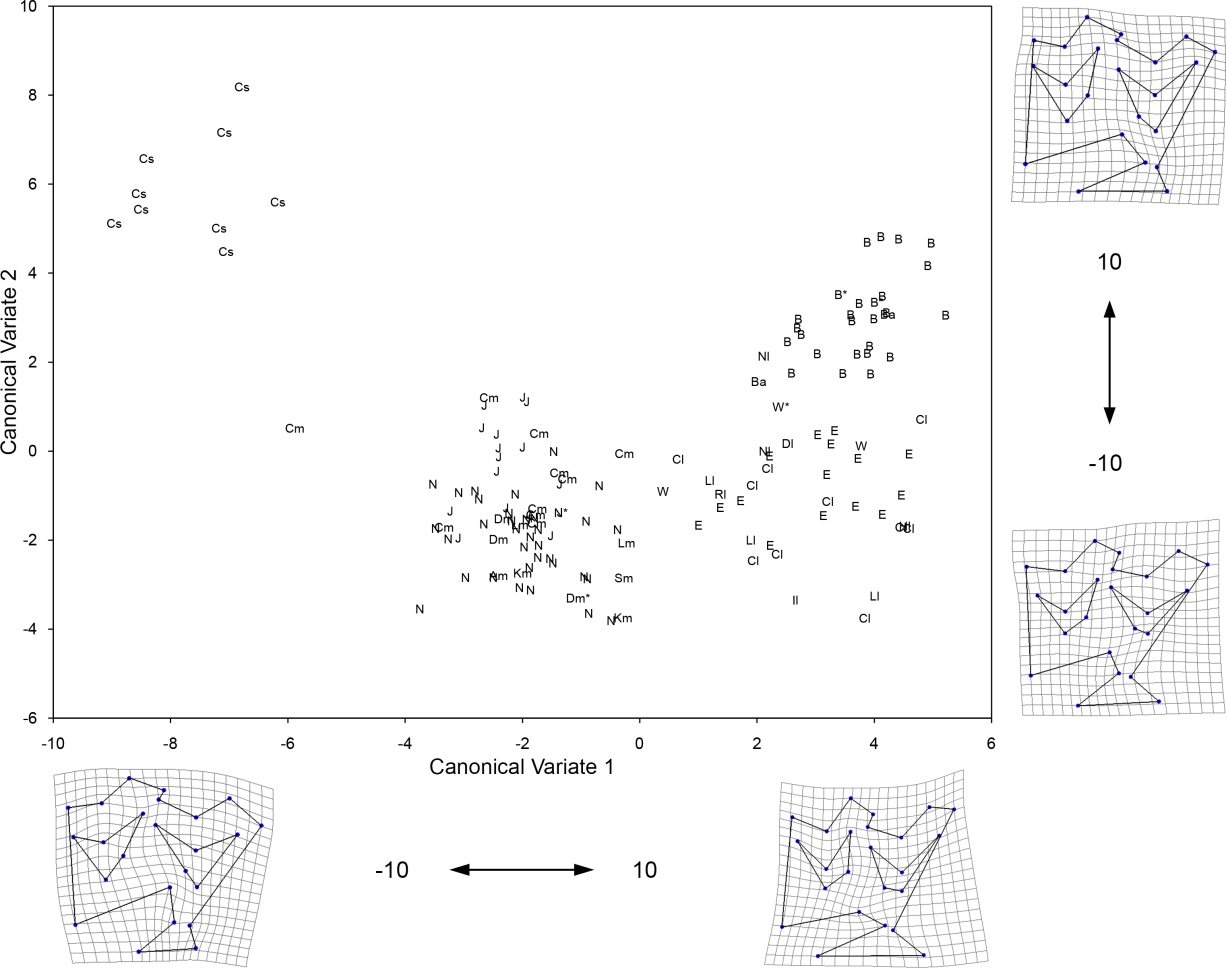
Plot of the first two Canonical Variates resulting from CVA of 24 landmark coordinates of the occlusal enamel pattern of the upper premolars (P3/P4). Shown on the margins of the graph is the change in tooth shape along each corresponding axis. The groups included in the analysis are: 1) large specimens from Cedral, Mexico (Cl); 2) medium specimens from Cedral (Cm) as well as all teeth from San Josecito Cave (J), Mexico; 3) small specimens from Cedral, Mexico (Cs); 4) large specimens from different localities of the American Southwest (identified by a lower case “l” beside the specimen symbol; refer to Fig 1 for locality names); 5) medium specimens from different localities of the American Southwest (identified by a lower case “m” beside the specimen symbol; refer to Fig 1 for locality names); 6) medium specimens from Natural Trap Cave, Wyoming (N); 7) large specimens from Natural Trap Cave, Wyoming (Nl); 8) large specimens from the Edmonton area gravel pits (E) and Wally’s Beach (W), Alberta; and 9) all of the specimens digitized from Bluefish Caves, Yukon (B). A lower case “a” beside the specimen symbol indicates a tooth that yielded aDNA (these include EQ38 and EQ45 from Bluefish Caves). An asterisk (*) beside the specimen symbol denotes a tooth associated (i.e., it belongs to the same individual) with a specimen from which aDNA was obtained (including teeth associated with EQ3 from Dry Cave, New Mexico, EQ9 from Natural Trap Cave, EQ43 from Wally’s Beach, and EQ44 as well as EQ47 from Bluefish Caves). Table A in S2 File lists all of the specimens included in this analysis.

**Fig 8:**
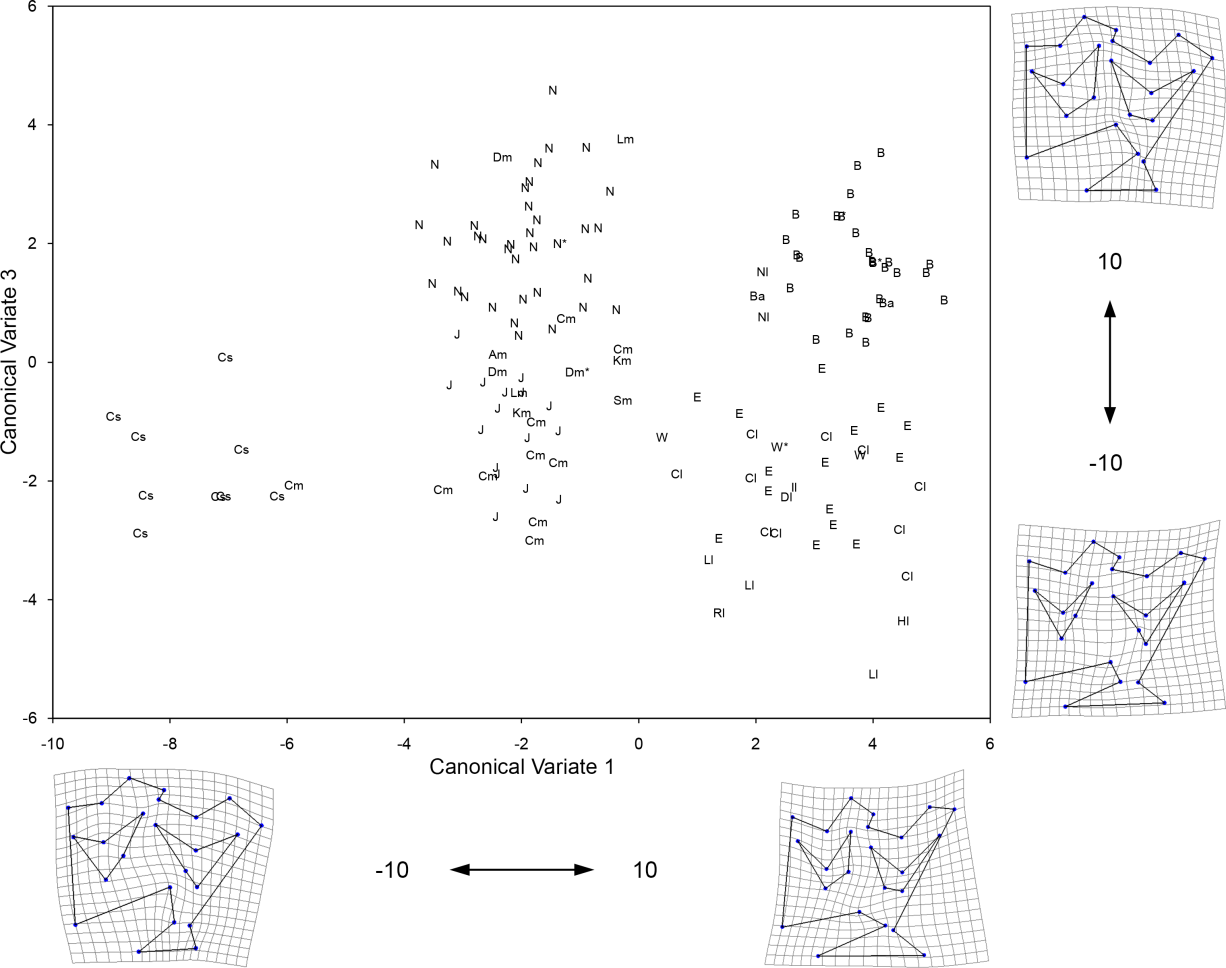
Plot of the first and third Canonical Variates resulting from CVA of 24 landmark coordinates of the occlusal enamel pattern of the upper premolars (P3/P4). Shown on the margins of the graph is the change in tooth shape along each corresponding axis. Refer to caption of Fig 7 for details on the specimens included in this analysis.

The first three Canonical Variates (CV 1 to CV 3) account for 81.84% of the relative between-group variation (Table 4). The different groups are arranged from small to large along CV 1 (Fig 7), largely reflecting the pattern seen in the PCA of the linear measurements. The transformation grids show that negative scores on CV 1 correspond to shallow parastyle-mesostyle and mesostyle-metastyle valleys, bucco-lingually expanded fossettes, relatively short protocones, and mesial displacement of landmark 11 (around the area where the pli caballin is located); the opposite is observed for positive CV 1 scores. The morphological characters associated with negative CV 1 scores are not typically found in extant caballine equids (e.g., [59]). As a result, we identify the groups that have negative CV 1 scores as possessing non-caballine tooth morphologies; these groups are: the small size group from Cedral, Mexico, as well as the intermediate size groups from northeastern Mexico (Cedral and San Josecito Cave), the American Southwest, and Natural Trap Cave. The remaing groups have caballine tooth morphologies (positive CV 1 scores) and these correspond to the large size groups from Cedral, Mexico, the American Southwest, Natural Trap Cave, and Alberta (Edmonton gravel pits and Wally’s Beach), as well as the specimens from Bluefish Caves, Yukon Territory (Fig 7). The second Canonical Axis (CV 2) clearly separates the small specimens from northeastern Mexico and the specimens from Bluefish Caves from the rest of the groups. Negative CV 2 scores reflect a mesial extension of the anterior margin of the protocone and a more prominent mesostyle (landmarks 3 and 4 are more separated from each other); the opposite is seen for positive CV 2 scores. The third Canonical Axis (CV 3) does not clearly separate any of the groups, but arranges the intermediate size groups from south to north: specimens from northeastern Mexico show negative scores, specimens from Wyoming have positive scores, and teeth from the American Southwest have an intermediate position (Fig 8). Examination of the transformation grid for the CV 3 axis, shows that negative scores correspond to a displacement away from the center of the tooth of the pli paraconule (landmark 17), pli postfossette (landmark 14), and landmark 11; the opposite is observed for positive CV 3 scores.

**Table 4:** Eigenvalues, percentage variance, and cumulative percentage variance of the first five Canonical Variates resulting from CVA of 24 landmark coordinates of the occlusal enamel pattern of the upper premolars (P3/P4).

	Eigenvalues	% Variance	Cumulative %
1	10.9984	43.64	43.64
2	6.0334	23.94	67.58
3	3.5944	14.26	81.84
4	1.8776	7.45	89.29
5	1.3469	5.34	94.64

The pair-wise permutation tests identified significant differences in the Procrustes distance for all but eight comparisons (Tables 5 and 6). Two of these comparisons concern the large size group from northeastern Mexico, which is not significantly different from the large size groups of the American Southwest and Alberta. Likewise, these last two groups are not statistically different from each other. The medium size group from Natural Trap Cave is not significantly different from the medium size group of the American Southwest. The four remaining pair-wise permutation tests that are non-significant include the large size group from Natural Trap Cave, which has a sample size of only two specimens and, thus, the reliability of these results is questionable.

**Table 5:** Procrustes distances among groups for the upper premolars (P3/P4).

	Cl	B	Cm/J	Cs	SWl	SWm	Nl	N
B	0.0606							
Cm/J	0.0847	0.0649						
Cs	0.1354	0.1099	0.0683					
SWl	0.0358	0.0614	0.0870	0.1351				
SWm	0.0618	0.0543	0.0412	0.0871	0.0657			
Nl	0.0634	0.0657	0.0758	0.1095	0.0745	0.0590		
N	0.0856	0.0698	0.0400	0.0672	0.0864	0.0370	0.0696	
E/W	0.0327	0.0455	0.0746	0.1247	0.0324	0.0538	0.0670	0.0780

Abbreviations: Cl = large specimens from Cedral, Mexico. Cm/J = medium specimens from Cedral and specimens from San Josecito Cave, Mexico; Cs = small specimens from Cedral, Mexico; SWl = large specimens from the American Southwest; SWm = medium specimens from the American Southwest; Nl = large specimens from Natural Trap Cave, Wyoming; N = medium specimens from Natural Trap Cave, Wyoming; E/W = large specimens from the Edmonton area and Wally’s Beach, Alberta; B = specimens from Bluefish Caves, Yukon.

**Table 6:** *P*-values from permutation tests (10,000 permutation rounds) for Procrustes distances among groups of the upper premolars (P3/P4).

	Cl	B	Cm/J	Cs	SWl	SWm	Nl	N
B	**< .0001**							
Cm/J	**< .0001**	**< .0001**						
Cs	**< .0001**	**< .0001**	**< .0001**					
SWl	0.3251	**< .0001**	**< .0001**	**< .0001**				
SWm	**0.0031**	**< .0001**	**0.0153**	**0.0007**	**0.001**			
Nl	0.2502	**0.0250**	**0.0244**	**0.0263**	**0.028**	0.4479		
N	**< .0001**	**< .0001**	**< .0001**	**< .0001**	**< .0001**	0.0514	0.0618	
E/W	0.1672	**< .0001**	**< .0001**	**< .0001**	0.2875	**0.0007**	0.0801	**< .0001**

Abbreviations: Cl = large specimens from Cedral, Mexico. Cm/J = medium specimens from Cedral and specimens from San Josecito Cave, Mexico; Cs = small specimens from Cedral, Mexico; SWl = large specimens from the American Southwest; SWm = medium specimens from the American Southwest; Nl = large specimens from Natural Trap Cave, Wyoming; N = medium specimens from Natural Trap Cave, Wyoming; E/W = large specimens from the Edmonton area and Wally’s Beach, Alberta; B = specimens from Bluefish Caves, Yukon. Statistically significant *p*-values are shown in bold.

### 3.3 Geometric morphometrics of the enamel pattern of lower premolars

The lower premolars show a marginally significant relationship between shape (as defined by the Procrustes coordinates) and log centroid size (p = 0.0437). The regression on centroid size accounts for 2.257% of the total shape variation. As for the case of the upper premolars, the residuals were calculated and used in further statistical analyses.

**Fig 9:**
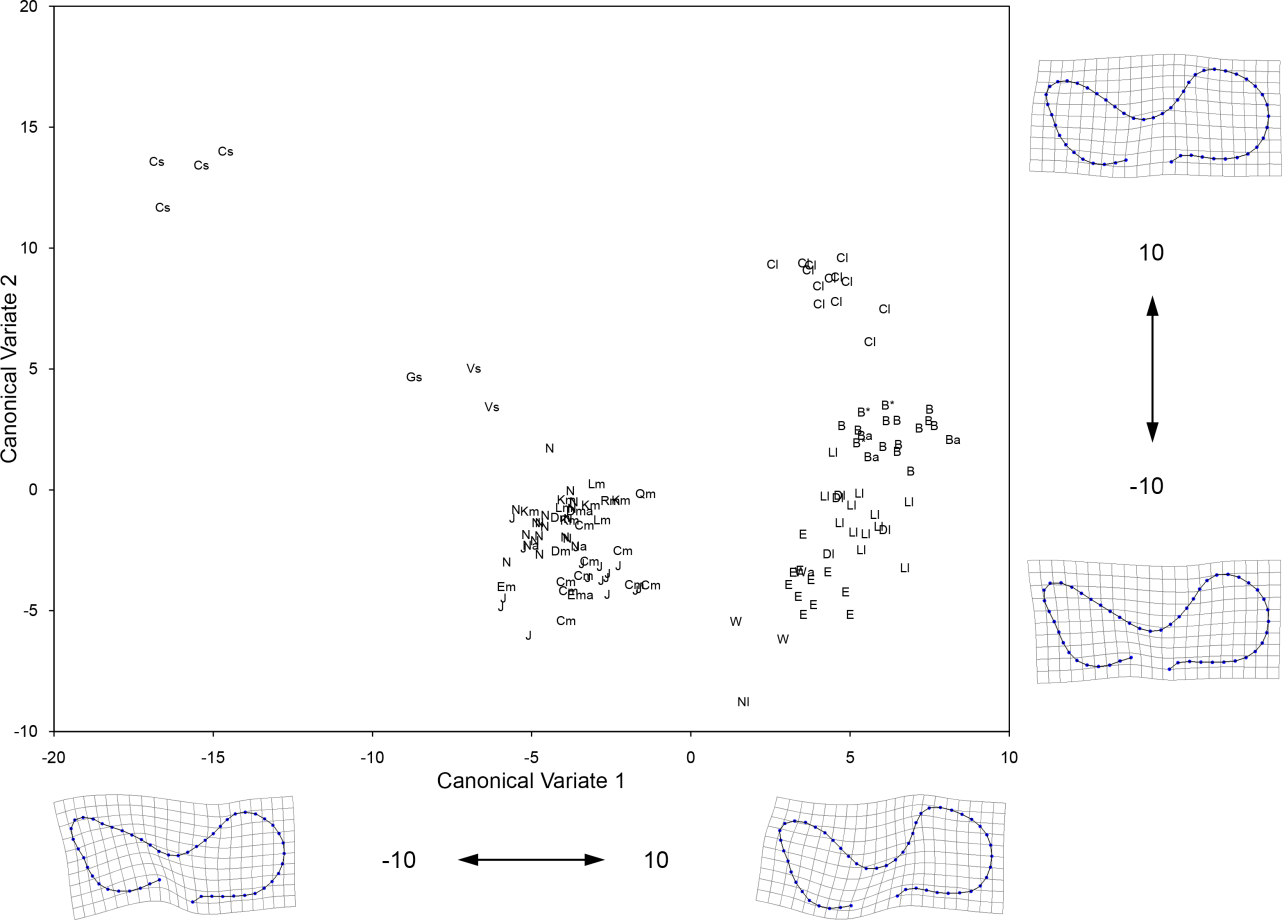
Plot of the first two Canonical Variates resulting from CVA of 50 landmark coordinates of the double knot (metaconid-linguaflexid-metastylid complex) of the lower premolars (p3/p4). Shown on the margins of the graph is the change in shape along each corresponding axis. The groups included in the analysis are: 1) large specimens from Cedral, Mexico (Cl); 2) medium specimens from Cedral (Cm) as well as all teeth from San Josecito Cave (J), Mexico; 3) small specimens from Cedral, Mexico (Cs); 4) large specimens from different localities of the American Southwest (identified by a lower case “l” beside the specimen symbol; refer to Fig 1 for locality names); 5) medium specimens from different localities of the American Southwest (identified by a lower case “m” beside the specimen symbol; refer to Fig 1 for locality names); 6) small specimens from Villa Ahumada (Vs) and Highway 45 (Gs), Chihuahua, Mexico; 7) medium specimens from Natural Trap Cave, Wyoming (N); 8) large specimens from Natural Trap Cave, Wyoming (Nl); 9) large specimens from the Edmonton area gravel pits (E) and Wally’s Beach (W), Alberta; 10) medium specimens from the Edmonton area gravel pits (Em), Alberta; and 11) all of the specimens digitized from Bluefish Caves, Yukon (B). A lower case “a” beside the specimen symbol indicates a tooth that yielded aDNA (these include EQ1 from Dry Cave, New Mexico, EQ4 from the Edmonton area gravel pits, EQ13 as well as EQ22 from Natural Trap Cave, EQ43 from Wally’s Beach, and EQ39, EQ48, and EQ50 from Bluefish Caves). An asterisk (*) beside the specimen symbol denotes a tooth associated (i.e., it belongs to the same individual) with a specimen from which aDNA was obtained (including teeth associated with EQ42, EQ51, and EQ53 from Bluefish Caves). Table B in S2 File lists all of the specimens included in this analysis.

**Fig 10:**
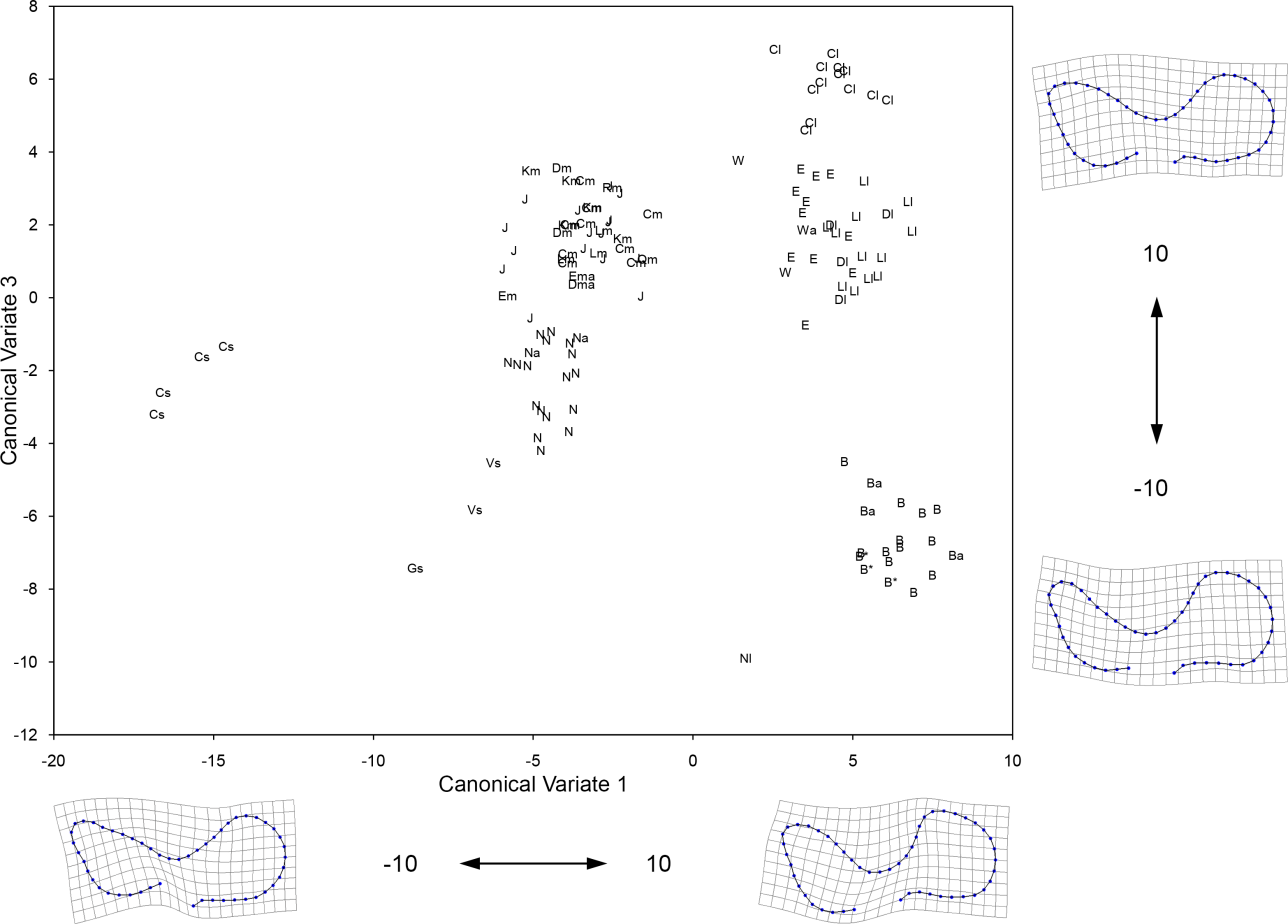
Plot of the first and third Canonical Variates resulting from CVA of 50 landmark coordinates of the double knot (metaconid-linguaflexid-metastylid complex) of the lower premolars (p3/p4). Shown on the margins of the graph is the change in shape along each corresponding axis. Refer to caption of Fig 9 for details on the specimens included in this analysis.

The first three Canonical Variates (CV 1 to CV 3) account for 71.93% of the relative between-group variation (Table 7). The groups generally plot along CV 1 from small to large (Fig 9), reflecting the same overall pattern observed in the CVA of the upper premolars and the PCA of the linear measurements. Examination of the transformation grids reveals that the CV 1 axis corresponds to a morphological gradient which goes from a caballine double knot, with a deep, U-shaped linguaflexid on the right side of the plot (i.e., CV 1 values greater than 0) to a hemione-like double knot with a shallow and more open, U-shaped linguaflexid on the left side of the plot (i.e., CV 1 values less than 0). Moreover, positive CV 1 scores also reflect a tooth morphology in which the metaconid is “constricted” (i.e., the bucco-distal margin of the metaconid is displaced towards the linguaflexid) and the metastylid is “open” (i.e., the bucco-mesial margin of the metastylid is displaced away from the linguaflexid); the opposite pattern is observed for specimens with negative CV 1 scores. As a result, we identify the groups that have positive CV 1 scores as possessing caballine tooth morphologies; these groups are: the large size groups from Cedral, Mexico, the American Southwest, Natural Trap Cave, and Alberta (Edmonton gravel pits and Wally’s Beach), as well as the specimens from Bluefish Caves, Yukon Territory. The remaing groups have non-caballine tooth morphologies (negative CV 1 scores) and these correspond to the small size groups from Cedral, Mexico, and the American Southwest (a small sample of teeth from northern Chihuahua, Mexico), as well as the intermediate size groups from northeastern Mexico (Cedral and San Josecito Cave), the American Southwest, Natural Trap Cave, and Alberta (a small sample of teeth from the Edmonton area gravel pits) (Fig 9). The small and large groups from northeastern Mexico, the small specimens from the American Southwest, and the specimens from Bluefish Caves all have positive CV 2 scores and are clearly separated from the rest of the groups along this axis (Fig 9). The transformation grids show that positive CV 2 scores correspond to a relatively rounded metastylid, whereas specimens with negative CV 2 scores reflect a triangular metastylid. The specimens from Bluefish Caves, the large and medium size specimens from Natural Trap Cave and the small groups from northeastern Mexico and the American Southwest all have negative CV 3 scores and plot separately from the remaining groups in the data set (Fig 10). Negative CV 3 scores correspond to a bucco-lingually compressed metastylid, whereas positive scores reflect a bucco-lingually expanded metastylid.

**Table 7:** Eigenvalues, percentage variance, and cumulative percentage variance of the first five Canonical Variates resulting from CVA of 50 landmark coordinates of the double knot (metaconid-linguaflexid-metastylid complex) of the lower premolars (p3/p4).

	Eigenvalues	% Variance	Cumulative %
1	30.9264	32.85	32.85
2	21.6312	22.98	55.83
3	15.1508	16.10	71.93
4	8.3076	8.83	80.75
5	5.6457	6.00	86.75

In contrast to the upper premolars, there were fewer pair-wise comparisons in which the Procrustes distance between groups was statistically different (Tables 8 and 9). This is partially due to the inclusion of groups with small sample sizes, namely the small groups from northeastern Mexico and the American Southwest, the large size group from Natural Trap Cave, and the medium size group from Alberta. Of the remaining groups in the data set, the most relevant differences are: 1) the Bluefish Caves group is significantly different from all other groups; 2) the medium size group from northeastern Mexico differs from the medium size group of Natural Trap Cave as well as the large size groups from northeastern Mexico, the American Southwest, and Alberta; 3) the medium size group from the American Southwest is statistically different from the large size groups of Alberta and the American Southwest; and 4) the medium size group from Natural Trap Cave differs from the large size group of Alberta.

**Table 8:** Procrustes distances among groups for the lower premolars (p3/p4).

	Cl	Em	B	Cm/J	Cs	SWl	SWm	SWs	Nl	N
Em	0.0894									
B	0.0865	0.1224								
Cm/J	0.1056	0.0762	0.1575							
Cs	0.1383	0.1224	0.2062	0.0688						
SWl	0.0369	0.0699	0.0849	0.0980	0.1389					
SWm	0.0838	0.0519	0.1302	0.0387	0.0892	0.0722				
SWs	0.1093	0.0963	0.1771	0.0728	0.0515	0.1089	0.0739			
Nl	0.1392	0.1072	0.1510	0.0898	0.1452	0.1236	0.0872	0.1494		
N	0.0698	0.0444	0.1111	0.0596	0.1069	0.0620	0.0321	0.0828	0.0977	
E/W	0.0511	0.0722	0.1008	0.1134	0.1443	0.0378	0.0857	0.1071	0.1483	0.0726

Abbreviations: Cl = large specimens from Cedral, Mexico. Cm/J = medium specimens from Cedral and specimens from San Josecito Cave, Mexico; Cs = small specimens from Cedral, Mexico; SWl = large specimens from the American Southwest; SWm = medium specimens from the American Southwest; SWs = small specimens from the American Southwest (Villa Ahumada and Highway 45, Chihuahua); Nl = large specimens from Natural Trap Cave, Wyoming; N = medium specimens from Natural Trap Cave, Wyoming; E/W = large specimens from the Edmonton area gravel pits and Wally’s Beach, Alberta; Em = medium specimens from the Edmonton area gravel pits, Alberta; B = specimens from Bluefish Caves, Yukon.

**Table 9:** *P*-values from permutation tests (10,000 permutation rounds) for Procrustes distances among groups of the lower premolars (p3/p4).

	Cl	Em	B	Cm/J	Cs	SWl	SWm	SWs	Nl	N
Em	0.5058									
B	**0.0406**	0.1796								
Cm/J	**0.0022**	0.3584	**< .0001**							
Cs	0.0784	0.4690	**0.0010**	0.2060						
SWl	0.5379	0.4552	**0.0068**	**0.0003**	**0.0141**					
SWm	0.0630	0.8363	**0.0001**	0.2552	0.1435	**0.0223**				
SWs	0.2377	0.6013	**0.0047**	0.2328	0.9087	0.0705	0.3682			
Nl	0.6303	1.0000	0.1799	0.4938	0.3942	0.2581	0.7017	0.7455		
N	0.0973	0.8424	**0.0017**	**0.0315**	0.0855	0.0571	0.5407	0.2564	0.6039	
E/W	0.2837	0.4330	**0.0009**	**< .0001**	**0.0143**	0.3376	**0.0062**	0.0659	0.1217	**0.0339**

Abbreviations: Cl = large specimens from Cedral, Mexico. Cm/J = medium specimens from Cedral and specimens from San Josecito Cave, Mexico; Cs = small specimens from Cedral, Mexico; SWl = large specimens from the American Southwest; SWm = medium specimens from the American Southwest; SWs = small specimens from the American Southwest (Villa Ahumada and Highway 45, Chihuahua); Nl = large specimens from Natural Trap Cave, Wyoming; N = medium specimens from Natural Trap Cave, Wyoming; E/W = large specimens from the Edmonton area gravel pits and Wally’s Beach, Alberta; Em = medium specimens from the Edmonton area gravel pits, Alberta; B = specimens from Bluefish Caves, Yukon. Statistically significant *p*-values are shown in bold.

### 3.4 Ancient mtDNA

We were able to extract and amplify ancient mtDNA from 22 of 50 late Pleistocene specimens we sampled (Table D in S2 File), including specimens of the different morphological groups identified in the morphometric analyses, except for the small non-caballine equids from northeastern Mexico and the American Southwest. The DNA sequence data we generated was submitted to GenBank (accession numbers KX137124 –KX137148).

**Fig 11:**
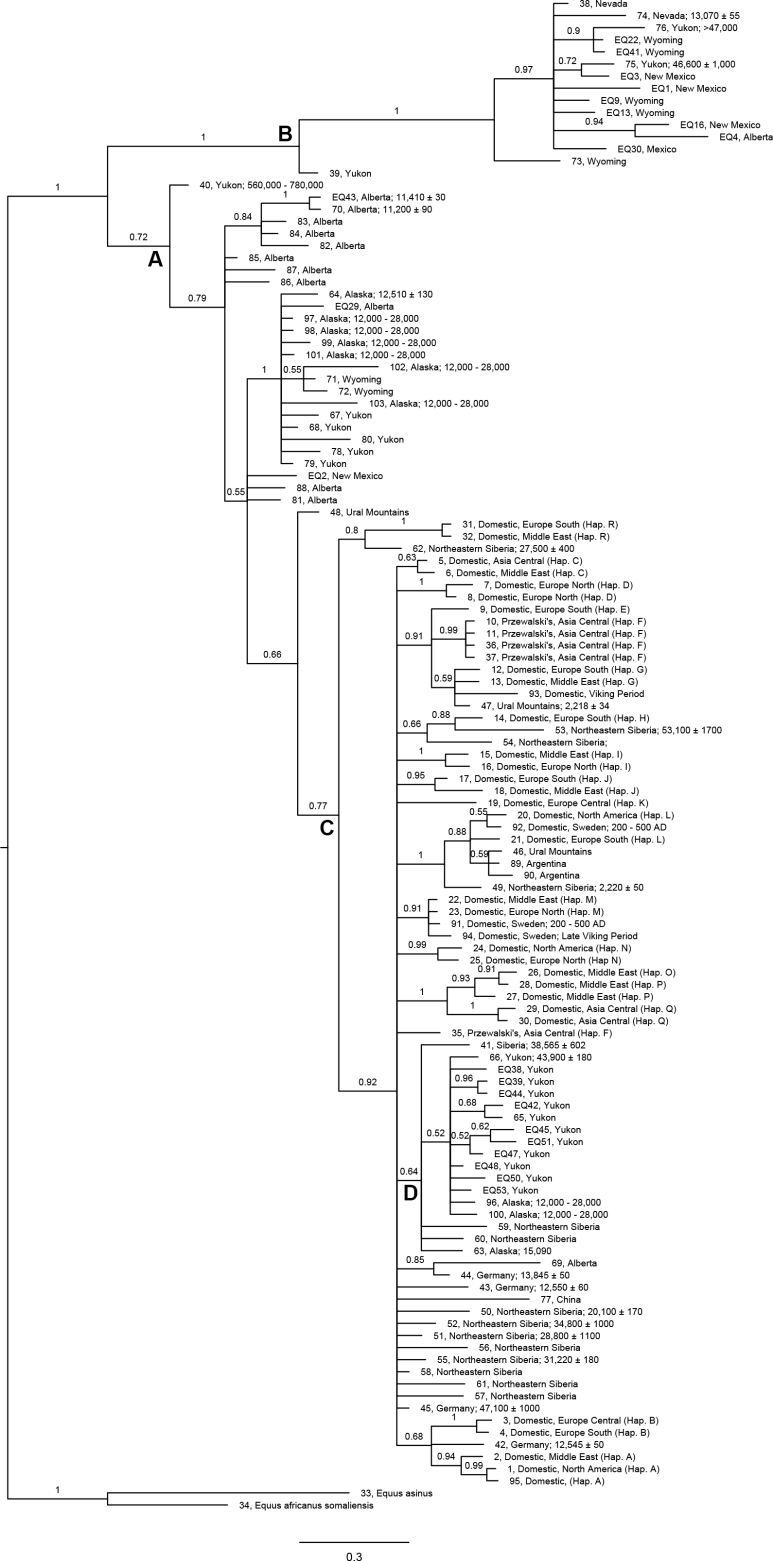
Consensus tree of Bayesian (Markov chain Monte Carlo) phylogenetic analysis displaying relationships between mitochondrial control region (HVR 1) haplotypes of extinct and extant equids, rooted with domestic donkey (*Equus africanus asinus* (L., 1758)) and Somali Wild Ass (*Equus africanus somaliensis* (Noack, 1884)) as the outgroup. The tree was constructed using 588 bp fragments of the HVR I. Posterior probabilities of the major nodes are listed for each of the branches. The groups discussed in the text are indicated by the letters. Tables D and F in S2 File list all of the specimens included in this analysis.

The Bayesian phylogenetic analysis using a 588 bp fragment of the HVR I yields the two lineages of late Pleistocene North American equids that have been identified in previous molecular studies [13, 97]: caballine and New World stilt-legged lineages (identified as clades A and B, respectively in Fig 11). The phylogenetic analysis also recovers 16 of the 18 extant horse haplogroups identified by Achilli et al. [96]. The two horse haplogroups that are not recovered in the analysis are haplogroups O and F.

Interestingly, the medium-sized specimens with a non-caballine tooth morphology from the Edmonton area, Alberta; Natural Trap Cave, Wyoming; Dry Cave, New Mexico; and San Josecito Cave, Mexico fall in the stilt-legged clade. The large-sized specimens with a caballine tooth morphology from Dry Cave, New Mexico and Wally’s Beach site, Alberta; as well as the specimens from Bluefish Caves, Yukon Territory, which also have a caballine tooth morphology cluster within the caballine clade (Fig 11). The caballine specimens from Wally’s Beach site and Dry Cave fall outside of the caballine crown group (clade C in Fig 11). The specimens from Bluefish Caves fall within the caballine crown group and may comprise an extinct horse haplogroup along with specimens from Siberia, Alaska, and other sites in the Yukon Territory (clade D in Fig 11).

## 4. Discussion

The geometric morphometric analyses of the occlusal enamel pattern of the upper and lower premolars suggest the presence of four morphological groups of *Equus* for the Western Interior of North America during the late Pleistocene. Overall, there is good correspondence between these groups and those suggested by the morphometric analysis of tooth dimensions. The geometric morphometric analyses allow a better characterization of the occlusal enamel pattern and facilitate comparisons with descriptions published in the literature; thus, our interpretations of tooth morphology are primarily based on the results of these analyses. Two of the groups identified possess morphological characters that are typically associated with caballine equids, such as a deep U-shaped linguaflexid (e.g., [8, 83–86]), whereas the remaining two groups possess non-caballine enamel patterns (Table 10). For the purpose of this discussion, the four morphological groups identified in our analyses are numbered as follows: Group 1) caballine equids from Bluefish Caves, Yukon Territory; Group 2) caballine equids from Alberta, Natural Trap Cave, the American Southwest, and Cedral, Mexico, which are morphologically different (Table 10) and also tend to be larger than the Bluefish Caves caballine group; Group 3) a non-caballine equid group from Cedral, Mexico, and the American Southwest (comprised of a small sample of teeth from northern Chihuahua), which shows features of the enamel pattern that are different from the other non-caballine group (Table 10) and that also includes the smallest specimens in our dataset; Group 4) a non-caballine equid group which tends to be intermediate in size (relative to the other groups) from northeastern Mexico (Cedral and San Josecito Cave), the American Southwest, Natural Trap Cave, and Alberta (comprised by a small sample of teeth from the Edmonton area gravel pits).

**Table 10:** Summary of the results of the geometric morphometric analyses of the cheek teeth and the Bayesian phylogenetic analysis of ancient mtDNA.

Group	Upper P3/P4	Lower p3/p4	mtDNA	Taxonomic id.
Group 1	Caballine. Deep parastyle-mesostyle and mesostyle-metastyle valleys, fossettes bucco-lingually compressed, landmark 11 displaced distally, anterior margin of protocone does not extend mesially	Caballine. Generally deep and U-shaped linguaflexid, constricted metaconid, and bucco-lingually compressed metastylid	Caballine	*E*. *ferus*
Group 2	Caballine. Deep parastyle-mesostyle and mesostyle-metastyle valleys, fossettes bucco-lingually compressed, landmark 11 displaced distally, anterior margin of protocone extends mesially	Caballine. Generally deep and U-shaped linguaflexid, constricted metaconid, and bucco-lingually expanded metastylid	Caballine	*E*. *ferus*
Group 3	Non-caballine. Shallow parastyle-mesostyle and mesostyle-metastyle valleys, fossettes bucco-lingually expanded, landmark 11 displaced mesially, anterior margin of protocone does not extend mesially	Non-caballine. Generally shallow and V- or broad U-shaped linguaflexid, open metaconid, and relatively rounded metastylid	—	*E*. *cedralensis*
Group 4	Non-caballine. Relatively shallow parastyle-mesostyle and mesostyle-metastyle valleys, fossettes sometimes bucco-lingually expanded, landmark 11 in some specimens displaced mesially, anterior margin of protocone extends mesially	Non-caballine. Generally shallow and V- or broad U-shaped linguaflexid, open metaconid, and triangular metastylid	NWSL	*E*. *conversidens*

Four groups are identified (two caballine and two non-caballine) based on the morphology of the occlusal enamel pattern of the third and forth upper premolars (P3/P4) and the morphology of the metaconid, linguaflexid, and metastylid of the third and fourth lower premolars (p3/p4). Two clades are recognized (caballine and New World stilt-legged, NWSL) based on the analysis of ancient mtDNA of the hypervariable region I. The last column presents the taxonomic identifications referred in the main text. Ancient DNA extraction of specimens identified as *E*. *cedralensis* failed.

The identification of four morphological groups of *Equus* for the late Pleistocene of the Western Interior of North America differs from the latest morphological revisions of the genus. Winans [10] identifies the presence of three equid species groups that were widely distributed throughout North America, including the Western Interior, during the late Pleistocene: *Equus alaskae* (Hay), 1913 (small and stout-legged species group), *E*. *francisci* Hay, 1915 (small and stilt-legged species group), and *E*. *laurentius* Hay, 1913 (large and stout-legged species group). In contrast, Azzaroli [12] recognizes nine species of equids that were present in the continent during this time interval, six of which he mentions have been found in localities from the Western Interior of North America. These are *E*. *fraternus* Leidy, 1860 and *E*. *conversidens* Owen, 1869 (short-legged equids which Azzaroli [12] considers were related to South American species of *Equus*), *E*. *excelsus* Leidy, 1858 (a large and stout-legged equid with a heavy skull and mandible), *E*. *niobrarensis* Hay, 1913 (a large equid with more slender limbs than *E*. *excelsus* as well as a more slender skull and mandible), *E*. *mexicanus* (Hibbard) 1955 (a large equid which according to Azzaroli [12] shares some skull features with species of *Equus* from South America), and *E*. *francisci* Hay, 1915 (a stilt-legged equid).

The analysis of ancient mtDNA is congruent in several respects with the results of the morphological analyses. Ancient mtDNA was successfully extracted, amplified, and sequenced from specimens belonging to each of the four groups identified in the morphological analyses, with the exception of group 3. The mtDNA analysis recovers the two main clades identified previously by Weinstock et al. [13] and which they refer to as the caballine and New World “stilt-legged” (NWSL) clades. The specimens referred to groups 1 and 2 from which mtDNA was obtained have sequences that identify them as belonging to the caballine clade. These results are consistent with the morphology of the enamel pattern as both groups have a caballine tooth morphology (Table 10). The phylogenetic analysis places the specimens from group 2 as stem caballines. The specimens from group 1 fall within the caballine crown group forming a distinct, apparently extinct haplogroup to those identified by Achilli et al. [96], along with specimens from Siberia, Alaska, and other sites in the Yukon Territory. However, the tree at this level is not well resolved and additional work applying a mitochondrial genomic approach or analysis of nuclear protein-coding genes is required to validate these patterns. As a result, we favor a conservative interpretation and regard morphological groups 1 and 2 as geographical variants of a single widely distributed caballine species.

The equid teeth assigned to group 4 from which mtDNA was recovered fall within the NWSL clade in the phylogenetic analysis. This result is consistent with the tooth morphology as specimens in this group have a non-caballine enamel pattern (Table 10). On the other hand, this result was unexpected as the group 4 specimens from Dry Cave, New Mexico and San Josecito Cave, northeastern Mexico, are not associated with slender metapodials (e.g., [10, 12, 20, 21, 22]). These results may suggest a certain degree of plasticity in the metapodial proportions of this group. Examination of the PCA graphs of Winans, (Figures 14.6C and 14.6D in [10]) lends support to this idea and hints at the presence of a geographical cline in which the degree of metapodial slenderness increases from San Josecito Cave to Natural Trap Cave, with the specimens from Dry Cave occupying an intermediate position. These graphs also show that specimens from Natural Trap Cave do not attain the degree of slenderness presented by other North American Pleistocene equid samples, such as those from Channing, Texas, referred to *E*. *semiplicatus* Cope, 1892, by some researchers [12, 44], and the holotypes of *E*. *calobatus* Troxell, 1915, and *E*. *francisci* Hay, 1915 [105]. Eisenmann et al. [44] have identified the two former species as the true North American stilt-legged equids, based not only on the degree of metapodial slenderness, but also on the presence of unique morphological characters. A geographical cline was also revealed in the occlusal enamel pattern of the upper P3/P4 by the geometric morphometric analysis. The third Canonical Axis (CV 3) arranged the group 4 specimens from south to north: specimens from northeastern Mexico have negative scores, whereas specimens from Wyoming have positive scores, with specimens from the American Southwest occupying an intermediate position (Fig 8).

The results of the morphological and molecular analyses support the presence of two equid species for the Western Interior of North America during the late Pleistocene, a caballine species (morphological groups 1 and 2) and a non-caballine species (morphological group 4). A third species might be represented by morphological group 3, which has a distinctive enamel pattern. In both geometric morphometric analyses the first Canonical Variate (CV 1) separates caballine equids, which show positive scores, from non-caballine equids, which show negative scores (Figs 7 and 9). The group 3 specimens have the most negative scores in the plot. This equid is tentatively identified as a separate non-caballine species, however, the recovery and analysis of ancient DNA is required to test its validity.

### 4.1 Taxonomic nomenclature and geographic distribution of late Pleistocene equids from the Western Interior of North America

The taxonomy of North American *Equus* is highly confused and its resolution is beyond the scope of this study, which would require careful re-evaluation of every single holotype. Previous researchers (e.g., [4, 10]) have lamented that several holotypes consist of isolated teeth or partial tooth rows and have questioned the diagnostic value of these elements, regarding the names based on them as *nomina dubia*. The methodology presented here can be applied to evaluate many of these holotypes. This new research direction can potentially help to clarify the nomenclature of North American Pleistocene equids. Until such a study is completed, the name we use (i.e., *Equus cedralensis*) for the putative non-caballine species (morphological group 3 above) is considered provisional.

The caballine equid species appears to be conspecific with *E*. *ferus* Boddaert, 1785, and this is the name we propose should be assigned to this material. We regard the morphological differences between the enamel pattern of the caballine specimens from Bluefish Caves (morphological group 1) and the caballine specimens from the other geographic regions (morphological group 2) as the product of geographic variation. *Equus lambei* Hay, 1917, is the name that has been widely applied in the literature for the caballine equid material from Bluefish Caves (e.g., [54, 55]). Winans [10] suggested that *E*. *lambei* might be a junior synonym of *E*. *alaskae* (Hay), 1913, along with the specimens from San Josecito Cave referred as *E*. *conversidens leoni* by Stock [20, 21]. The synonymy with *E*. *alaskae* may or may not be correct, but the material from San Josecito Cave is clearly distinct based on the morphological and molecular analyses reported here. Azzaroli [11, 12] considered *E*. *lambei* as a valid species, but thought it was probably a subspecies of *E*. *niobrarensis* Hay, 1913. The caballine equid remains we studied from Alberta, Natural Trap Cave, the American Southwest, and northeastern Mexico have been identified under several names including *Equus caballus caballus* Linnaeus, 1758, *E*. *caballus laurentius* Hay 1913, *E*. *excelsus* Leidy, 1858, *E*. *laurentius* Hay, 1913, *E*. *mexicanus* (Hibbard), 1955, *E*. *midlandensis* Quinn, 1957, *E*. *niobrarensis*, and *E*. *scotti* Gidley, 1900 [4, 10, 22–24, 26, 27, 29–32]. Extant caballine equids have historically been assigned to two species, *E*. *caballus* Linnaeus, 1758, and *E*. *przewalskii* Poliakov, 1881, but several studies point to the inclusion of *E*. *przewaslkii* in *E*. *caballus* (e.g., [106–109]). The name *E*. *ferus* was proposed by Gentry et al. [110] to differentiate wild caballines from domestic forms (i.e., *E*. *caballus*). The International Commission on Zoological Nomenclature has approved this proposal [111, 112], and “implementation of the ruling means that names based on wild populations will continue to be used for wild species and will include those for domestic forms if these are considered conspecific” (p. 649 in [112]). We follow this proposal in the present study; however, we point out that there is still some disagreement about the status of *E*. *ferus* as a wild rather than a feral horse [113].

The non-caballine equid species (morphological group 4) whose ancient mtDNA corresponds to the NWSL clade of Weinstock et al. [13] is referred to *Equus conversidens* Owen, 1869. This name has been widely used in the literature of North American late Pleistocene equids, although not without some confusion (see Scott [114], for different morphological concepts of this species). The morphological and molecular data sets we analyzed for this species included several specimens studied by previous authors and which were consistently identified as *E*. *conversidens*, including material from San Josecito Cave (e.g., [11, 12, 20, 21, 114]), specimens from Dry Cave [22, 32], U-bar Cave [37], Scharbauer Ranch [30], and Blackwater Draw [31]. Alberdi et al. [27] also report the presence of *E*. *conversidens* from Cedral, Mexico; however, several of the specimens that Alberdi et al. [27] identify as *E*. *cedralensis* we identify here as *E*. *conversidens*. The specimens from Wally’s Beach, Alberta, were identified as *E*. *conversidens* by McNeil [49]; however, this assignment is not supported by the morphological and molecular analyses of the specimens from this site included in our study. The results show that the specimens from Wally’s Beach are caballine equids and are, therefore, identified as *E*. *ferus*. The NWSL equid from Natural Trap Cave, Wyoming, has been identified under different names, including *Amerhippus* sp., *E*. *alaskae*, and *Hemionus* sp. [10, 43, 44], but our results suggest that it is not distinct morphologically nor genetically from the NWSL equid of the American Southwest and Mexico; thus, it is here re-identified as *E*. *conversidens*. The same is suggested by the phylogenetic analysis for the NWSL equid from Beringia. The proposal put forward by different authors (e.g., [8, 12, 22]) regarding the close phylogenetic affinity of *E*. *conversidens* to South American equids of the subgenus *Amerhippus* (sometimes regarded as a distinct genus (e.g., [44])), based primarily on specimens from San Josecito Cave, is not supported by the molecular analysis. The specimens of *Equus (Amerhippus) neogeus* cluster well within the caballine clade (Fig 11) as it was originally reported by Orlando et al. [98]. Nevertheless, this should be tested further with additional mitochondrial and nuclear DNA data.

Fossil material referred here to *Equus conversidens* was recognized in four of the five geographic regions studied. It is well represented in northeastern Mexico (including Cedral and San Josecito Cave), the American Southwest (i.e., Algerita Blossom Cave, Blackwater Draw, Dark Canyon Cave, Dry Cave, Lubbock Lake, Quitaque Creek, Salt Creek, Scharbauer Ranch, and U-Bar Cave), and Natural Trap Cave, Wyoming. This species is much less common in Alberta, where it was identified based on at least four specimens from the Edmonton area gravel pits, but not from Wally’s Beach, and it was not found in the material examined from Bluefish Caves, Yukon. The presence of this species in the Edmonton area gravel pits is further supported by the association of some of the specimens studied (right and left p2 as well as left p3) as part of a partial dentary (RAM P98.5.480) in which all of the incisors lack an infundibulum (a funnel-like cup of enamel filled with cementum). Our examination of partial mandibles and mandibular symphyses with lower incisors from San Josecito Cave (e.g., LACM 18404, 18383, 18802, 120758, 18644) and those identified as *E*. *conversidens* from Dry Cave (UTEP 22–955, 26–1064) by Harris and Porter [22] and Harris [32] revealed that all of the incisors lack an infundibulum. In contrast, the mandibles and partial mandibles we observed with associated lower incisors that we assign to *E*. *ferus* from Bluefish Caves (e.g., CMH MgVo-2 B3-3-23, MgVo-2 C3(E)-3-19, MgVo-2 H6-3-7, MgVo-3 85–95, MgVo-3 85–76, MgVo-3 85–64, MgVo-3 M-9-83), Wally’s Beach (RAM DhPg-8 876.1, DhPg-8 863, DhPg-8 3437.2), the Edmonton area gravel pits (RAM P97.11.2A), Dry Cave (UTEP 22–1657), Salt Creek (UTEP 34–5), and Scharbauer Ranch (TMM 998–1) have an infundibulum on the first and second lower incisors and this feature is more variable on the third lower incisors. This pattern is certainly consistent with the results obtained for the molecular and morphological analyses of the cheek teeth; nevertheless, the sample size represented by these specimens is not adequate to fully document the frequency of this morphological trait in each species and further study is required. Eisenmann [115] has noted that the frequency of infundibula in the lower incisors of modern equid species can show important intraspecific variation. Moreover, as with other morphological characters of the enamel pattern of equid teeth, the morphology of the infundibulum changes as the tooth wears down until it completely disappears; therefore, the assessment of this character has to take into consideration the stage of tooth wear.

The taxonomic assignment of the small non-caballine equid (morphological group 3) from Cedral, northeastern Mexico, and northern Chihuahua, Mexico, here grouped with the American Southwest samples, is not completely clear. Morphologically, it appears to represent a separate species, but this needs to be validated with the sequencing and analysis of ancient DNA. Alberdi et al. [27] considered that the small equid from Cedral represents a new species, which they named *Equus cedralensis*, but the enamel pattern of the premolars as well as the tooth dimensions are comparable to those of *E*. *tau* Owen, 1869. The maxillary figured and described by Owen [116] (designated the lectotype of *E*. *tau* by Mooser and Dalquest [117]) has the third premolar damaged, but the fourth premolar shows many of the traits found in the group 3 teeth from Cedral identified in the geometric morphometric analysis: mesostyle and parastyle not prominent, shallow parastyle-mesostyle valley (the mesostyle-metastyle valley is not preserved in Owen’s [116] specimen), and the region of the occlusal enamel corresponding to landmark 11 displaced mesially (CV1 transformation grid in Fig 7). Other morphological traits commonly present in the group 3 teeth from Cedral and shared with the cheek teeth figured by Owen [116] are a straight (flat) lingual border of the protocone and the absence of a pli caballin. All of the features mentioned above are also present in the holotype of *E*. *francisci* figured by Lundelius and Stevens [105] and Eisenmann et al. [44]. The holotype of *E*. *tau* has been lost [117]; nevertheless, different researchers have assigned material from a variety of localities in Mexico and the United States to this species (e.g., [9, 24, 117]), some of which may or may not correspond to the species described by Owen [116]. Recently, Eisenmann et al. [44] suggested a partial skull from the Cedazo fossil assemblage, central Mexico, as the neotype of *E*. *tau*. Our examination of this specimen confirmed that the teeth are within the size range of the group 3 specimens and those figured by Owen [116], but they are in an advanced stage of wear and the premolars are too worn for reliable comparisons of their enamel pattern with the teeth in our analyses. The most common morphological concept of *E*. *tau* in the literature is that of a small-sized equid with slender metapodials (e.g., [9, 44]) and, as a result, some researchers have synonymised *E*. *francisci* with *E*. *tau* (e.g., [9]). It is not clear, however, whether *E*. *tau* possessed slender metapodials as the material described by Owen [116] was not associated with metapodials or any other postcranial elements. The same is true for the neotype proposed by Eisenmann et al. [44]. Although a slender metatarsal of the size range expected for the neotype skull of *E*. *tau* has been recovered from Cedazo [44, 117], its association with the skull cannot be firmly established as neither possess precise stratigraphic information. Stratigraphic studies at several of the localities that have produced the Cedazo fossil assemblage identified different fossiliferous strata, which range biochronologically from the latest Blancan to the Rancholabrean NALMAs (early to late Pleistocene) [118, 119]. This work suggests that what was originally described as the Cedazo local fauna by Mooser and Dalquest [117] actually includes specimens of different ages. It is, therefore, possible that the proposed neotype skull of *E*. *tau* and the small slender metatarsal are from different strata. According to Alberdi et al. [27], *Equus cedralensis* does not possess slender metapodials. Until the exact taxonomic status of *E*. *tau* and other small North American equids (e.g., *E*. *littoralis* Hay, 1913; *E*. *achates* Hay and Cooke, 1930) is clarified we provisionally refer to the morphological group 3 equid as *E*. *cedralensis*.

Extant equid species are mostly allopatric, except for small areas where two species are known to coexist (e.g., [120–122]). In contrast to the modern distribution of equids, the fossil record shows that throughout much of the history of the group in North America the coexistence of two or more species was the norm rather than the exception [104]. This pattern may have extended into the late Pleistocene. The coexistence of the species identified here as *E*. *ferus* and *E*. *conversidens* in some areas of the Western Interior is suggested by stratigraphic and biochronologic information, with associated radiocarbon dates, from localities such as Dry Cave [38], U-bar Cave [38], and Blackwater Draw [28, 31]. At Cedral, Mexico, these two equids co-occur with *E*. *cedralensis* [27] and recent paleodietary reconstructions suggest that resource partitioning may have facilitated their coexistence [123]. The restriction of *E*. *cedralensis* to lower latitudes of the Western Interior may seem surprising compared to the distribution of *E*. *ferus* and *E*. *conversidens*; however, our study evaluated specimens from a paleontologically short time interval (primarily mid- to late-Wisconsin glacial stage, *ca*. 50,000 to 10,000 radiocarbon years BP) and it is possible that this species had a wider distribution at other times. Alternatively, *E*. *cedralensis* may have been a resident of lower latitudes throughout most of its evolutionary history. Some North American Pleistocene mammals appear to be restricted to southern localities including pronghorn (*Capromeryx* spp. and *Stockoceros conklingi*), gomphotheres (*Cuvieronious* spp.), capybaras (*Hydrochoerus* spp. and *Neochoerus* spp.), glyptodonts (*Glyptotherium* spp.), camelids (*Palaeolama mirifica*), and pampatheres (*Pampatherium mexicanum* and *Holmesina* spp.) [1–3].

## 5. Conclusions

Two equid species, *Equus ferus* and *E*. *conversidens*, are identified for the late Pleistocene of the Western Interior of North America, based on molecular and morphological analyses of the cheek teeth. A third species, *E*. *cedralensis*, is provisionally recognized based exclusively on the morphological analyses of the cheek teeth. *Equus ferus* is a caballine equid that appears to have been distributed throughout much of the Western Interior of North America. It was identified from Cedral, Mexico, the American Southwest (e.g., Blackwater Draw, Dry Cave, Isleta Cave No. 2, Salt Creek, Scharbauer Ranch, and U-Bar Cave), Natural Trap Cave (where it is represented by relatively few specimens), Alberta (including the Edmonton area gravel pits and Wally’s Beach site), and the Bluefish Caves, Yukon Territory. Geographic variation in morphology in this species is indicated by statistically different occlusal enamel patterns in the specimens from Bluefish Caves relative to the specimens from the other geographic regions. Whether this represents ecomorphological variation and/or a certain degree of geographic and genetic isolation of these Arctic populations requires further study. *Equus conversidens* is a non-caballine equid which was previously identified based on ancient mtDNA as the New World stilt-legged clade [13]. The assignment to this group by the morphometric and ancient mtDNA analyses of specimens that are not associated with slender metapodials (e.g., specimens from Dry Cave, New Mexico and San Josecito Cave, northeastern Mexico [10, 12, 20, 21, 22]), may suggest a certain degree of plasticity in the metapodial proportions of this species. Specimens identified by our analyses as *E*. *conversidens* come from northeastern Mexico (Cedral and San Josecito Cave), the American Southwest (e.g., Blackwater Draw, Dark Canyon Cave, Dry Cave, Quitaque Creek, Salt Creek, Scharbauer Ranch, and U-Bar Cave), Natural Trap Cave, and Alberta (a small sample of teeth from the Edmonton area gravel pits). Specimens assigned on morphological grounds to a different non-caballine species are provisionally identified as *E*. *cedralensis*. We were unable to recover ancient mtDNA from teeth assigned to this species to corroborate its identification as a distinct species. *Equus cedralensis* appears to have been restricted during the late Pleistocene to the southern latitudes of the Western Interior of North America, as the specimens assigned to this species come from Cedral, Mexico, and the American Southwest (sites located in northern Chihuahua, Mexico).

The analyses of tooth morphology and ancient mtDNA reported in this study have provided new insights into the taxonomy of late Pleistocene equids from the Western Interior of North America. Nevertheless, additional work is needed to validate the patterns presented in our study. Of particular importance is the application of a mtDNA genomic approach to better resolve the phylogenetic relationships within the caballine and stilt-legged clades. Equally relevant is the morphological and, if possible, genomic assessment of several holotypes that consist of isolated teeth or partial tooth rows. Another important aspect is to increase the geographic coverage of the equid sample. Some late Pleistocene morphospecies identified in previous studies appear to be restricted to areas outside of the Western Interior of North America. For example, *E*. *occidentalis* sensu Merriam, 1913, which is commonly identified in late Pleistocene sites from the Pacific Coast of North America (e.g., Rancho La Brea, California) is considered a separate species from the ones described here based on the morphology of the upper P3/P4 premolars [25] as well as the lack of infundibulae on the lower incisors and other cranial characters [11, 12, 124]. Finally, we acknowledge that the temporal coverage of sampling needs to be increased. All of the specimens studied are late Pleistocene in age, mostly from the mid- to late-Wisconsin glacial stage (approximately 50,000 to 10,000 radiocarbon years BP). Further work on these areas will provide a refined understanding of the evolution and extinction of North American Pleistocene equids.

## Supporting information

S1 File Linear measurements of horse teeth studied (Tables A–H).
(XLSX)

S2 File Results of principal component analysis (Figs A–T), upper and lower horse teeth included in the geometric morphometric analyses (Tables A and B), landmarks used in the geometric morphometric analysis of the upper P3/P4 teeth (Table C), equid teeth sampled for ancient mtDNA (Table D), primers used (Table E), and horse mtDNA sequences compiled from the literature used in the Bayesian phylogenetic analysis (Table F).
(PDF)

S3 File Geometric morphometric analysis upper P3/P4 data set. (TPS)

S4 File Geometric morphometric analysis lower p3/p4 data set. (TXT)
