# Supplementary material for: Cheek tooth morphology and ancient mitochondrial DNA of late Pleistocene horses from the western interior of North America: Implications for the taxonomy of North American Late Pleistocene Equus
Source: PLoS One. 2017 Aug 17;12(8):e0183045. doi: 10.1371/journal.pone.0183045 (PMC5560644; doi:10.1371/journal.pone.0183045)
Supplement: S2 File — Results of principal component analysis (Figs A–T), upper and lower horse teeth included in the geometric morphometric analyses (Tables A and B), landmarks used in the geometric morphometric analysis of the upper P3/P4 teeth (Table C), equid teeth sampled for ancient mtDNA (Table D), primers used (Table E), and horse mtDNA sequences compiled from the literature used in the Bayesian phylogenetic analysis (Table F). (PDF) [file pone.0183045.s002.pdf]

Cheek Tooth Morphology and Mitochondrial Ancient DNA of Late Pleistocene Horses from the Western Interior of North America: Implications for the Taxonomy of North American Late Pleistocene *Equus*

Christina I. Barrón-Ortiz, Antonia T. Rodrigues, Jessica M. Theodor, Brian P. Kooyman, Dongya Y. Yang, and Camilla F. Speller.

Supporting Information S2 File

Table of Contents

Figure A. Principal component plots showing upper teeth from Cedral and San Josecito Cave, Mexico....3

Figure B. Principal component plots showing lower teeth from Cedral and San Josecito Cave, Mexico....4

Figure C. Principal component plots showing upper teeth from the American Southwest...5

Figure D. Principal component plots showing lower teeth from the American Southwest...6

Figure E. Principal component plots showing upper teeth from Natural Trap Cave, Wyoming....7

Figure F. Principal component plots showing lower teeth from Natural Trap Cave, Wyoming....8

Figure G. Principal component plots showing upper teeth from the Edmonton area and Wally's Beach, Alberta....9

Figure H. Principal component plots showing lower teeth from the Edmonton area and Wally's Beach, Alberta....10

Figure I. Principal component plots showing upper teeth from Bluefish Caves, Yukon....11

Figure J. Principal component plots showing lower teeth from Bluefish Caves, Yukon....12

Figure K. Histograms showing the distribution of PC 1 scores of upper teeth from Cedral and San Josecito Cave, Mexico....13

Figure L. Histograms showing the distribution of PC 1 scores of lower teeth from Cedral and San Josecito Cave, Mexico. 14

Figure M. Histograms showing the distribution of PC 1 scores of upper teeth from different localities of the American Southwest. 15

Figure N. Histograms showing the distribution of PC 1 scores of lower teeth from different localities of the American Southwest. 16

Figure O. Histograms showing the distribution of PC 1 scores of upper teeth from Natural Trap Cave, Wyoming. 17

Figure P. Histograms showing the distribution of PC 1 scores of lower teeth from Natural Trap Cave, Wyoming. 18

Figure Q. Histograms showing the distribution of PC 1 scores of upper teeth from the Edmonton area and Wally's Beach, Alberta. 19

Figure R. Histograms showing the distribution of PC 1 scores of lower teeth from the Edmonton area and Wally's Beach, Alberta. 20

Figure S. Histograms showing the distribution of PC 1 scores of upper teeth from Bluefish Caves, Yukon. 21

Figure T. Histograms showing the distribution of PC 1 scores of lower teeth from Bluefish Caves, Yukon. 22

Table A. Late Pleistocene equid specimens (upper teeth) included in the geometric morphometric analysis. 23

Table B. Late Pleistocene equid specimens (lower teeth) included in the geometric morphometric analysis. . 27

Table C. Landmarks used in the geometric morphometic analysis of the upper P3/P4 teeth. 30

Table D. Equid specimens that were sampled for ancient mtDNA. 31

Table E. Primers used to amplify a 621 bp fragment of the HVR I, mitochondrial control region. 33

Table F. Equid sequences compiled from various publications that were used in the Bayesian phylogenetic analysis of ancient mtDNA. . 34

References. . 38

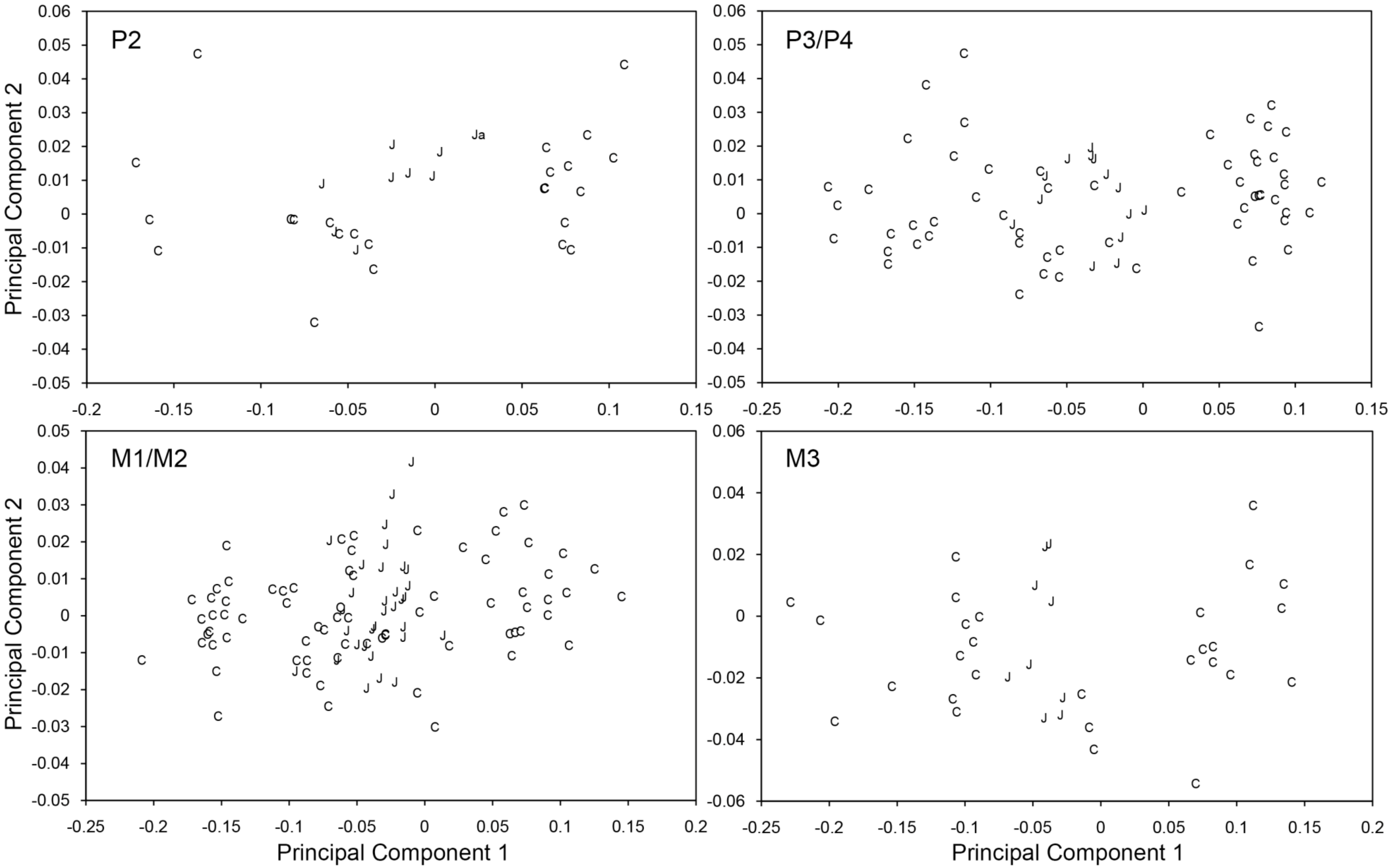

Figure A. Principal component plots showing upper teeth from Cedral (C) and San Josecito Cave (J), Mexico. The P2 specimen that yielded aDNA is identified by "a" and corresponds to EQ30 of the aDNA analysis.

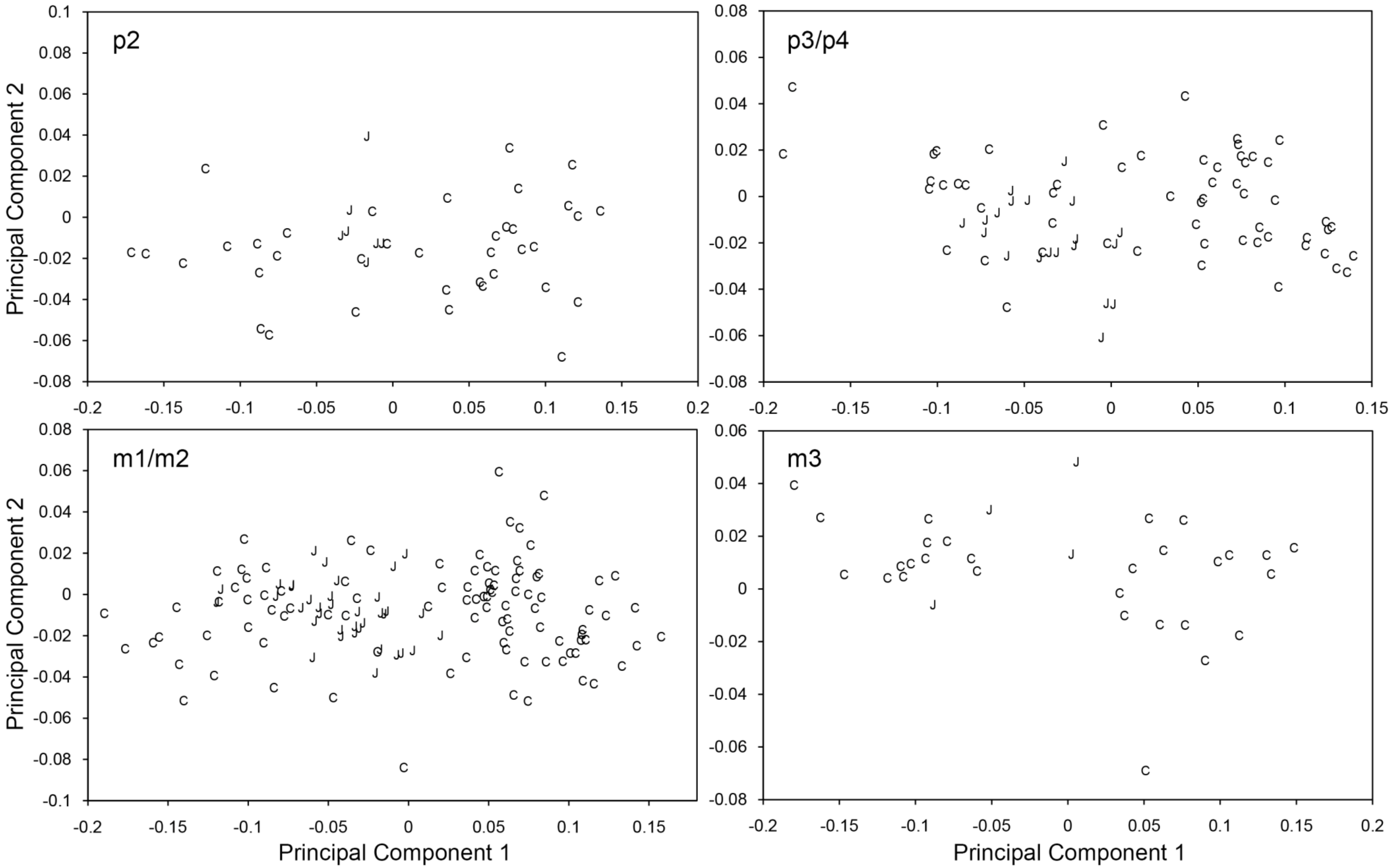

Figure B. Principal component plots showing lower teeth from Cedral (C) and San Josecito Cave (J), Mexico.

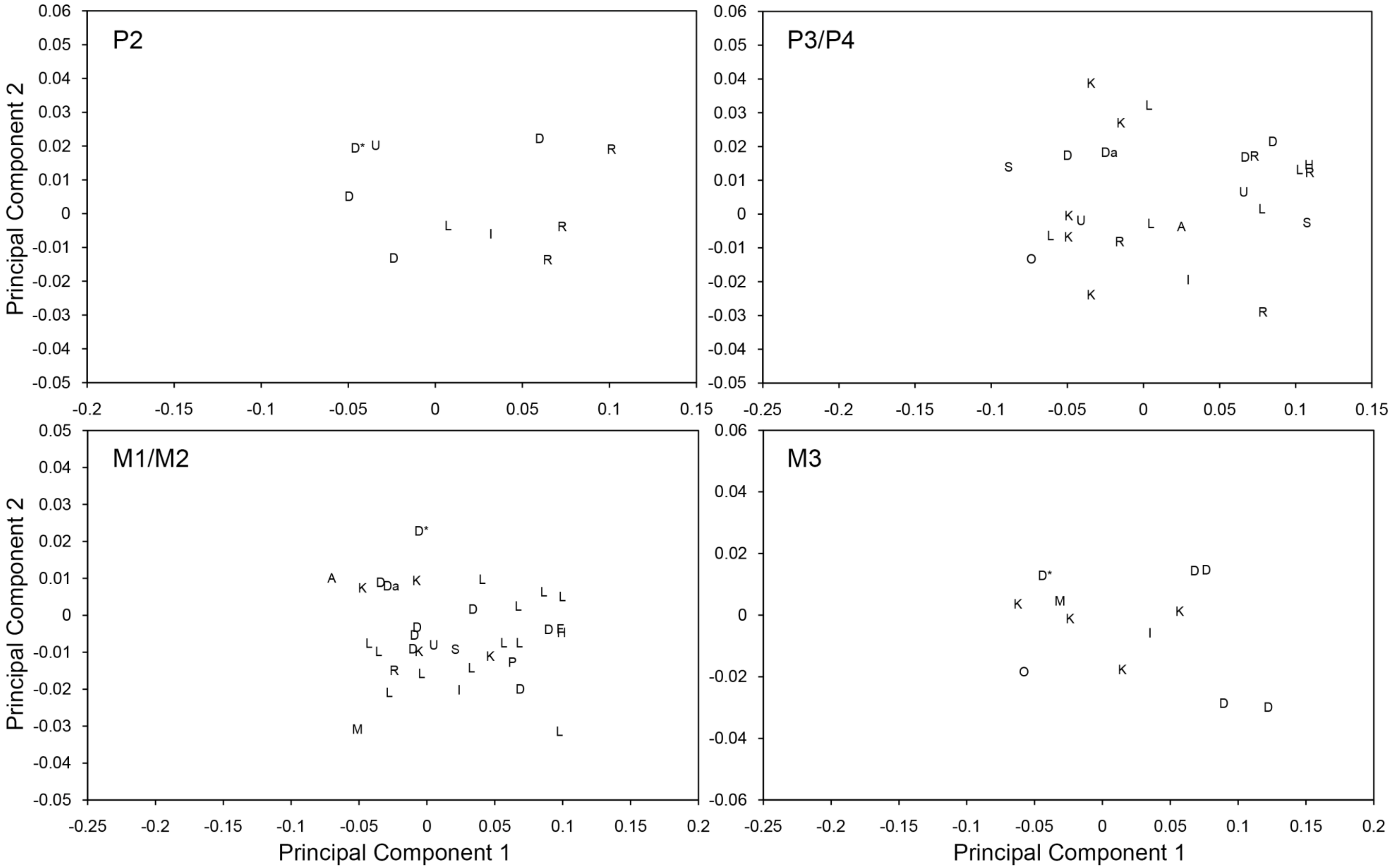

Figure C. Principal component plots showing upper teeth from the American Southwest. Refer to Figure 1 for locality abbreviations. Specimens that yielded aDNA (EQ3 and EQ16) and teeth associated with these specimens are identified by “a” and “*”, respectively.

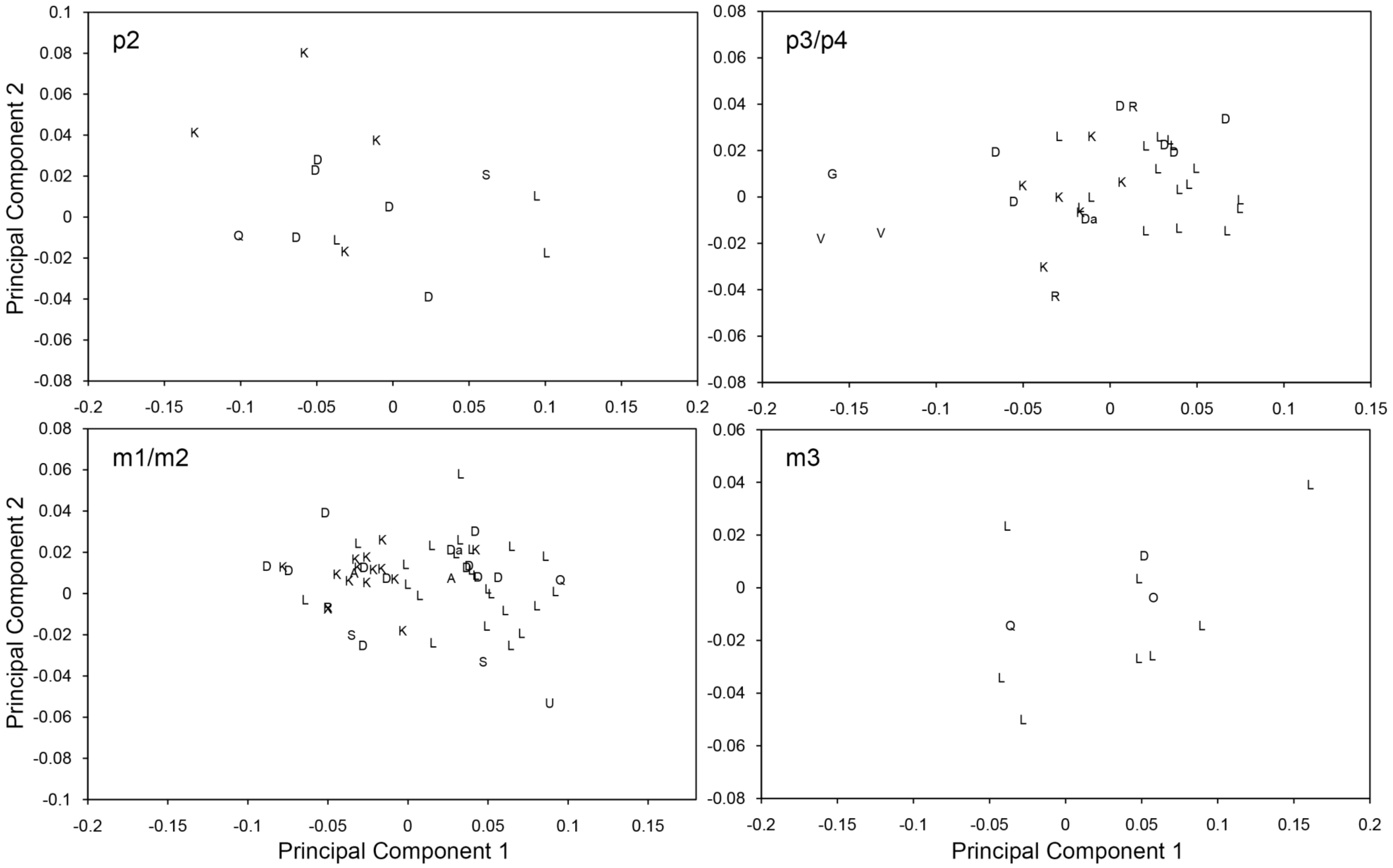

Figure D. Principal component plots showing lower teeth from the American Southwest. Letters indicate specimens according to locality provenance (Figure 1 of the main text). Specimens that yielded aDNA (EQ1 and EQ2) are identified by “a”.

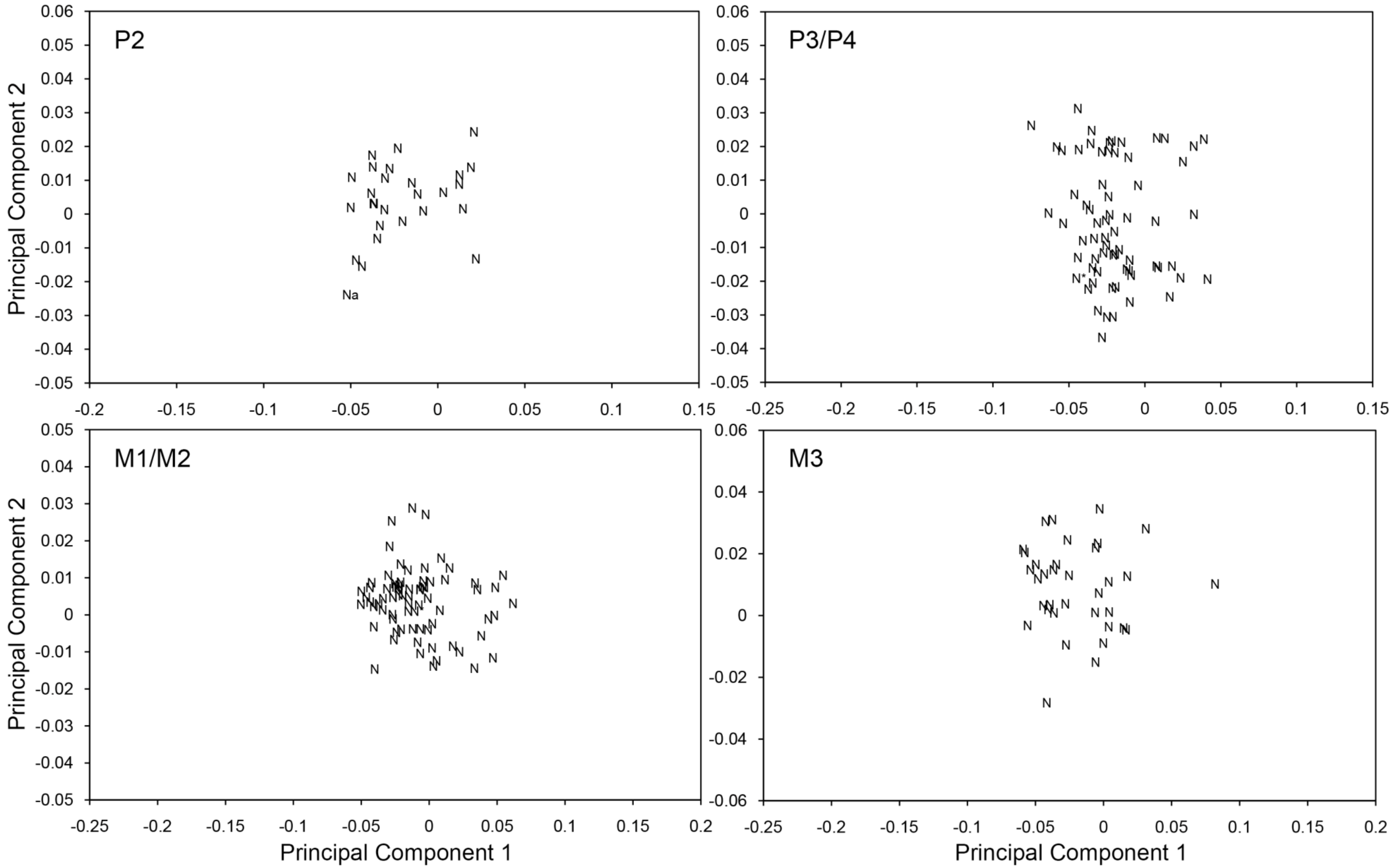

Figure E. Principal component plots showing upper teeth from Natural Trap Cave, Wyoming. The P2 specimen that yielded aDNA (EQ9) and teeth associated with it are identified by “a” and “*”, respectively.

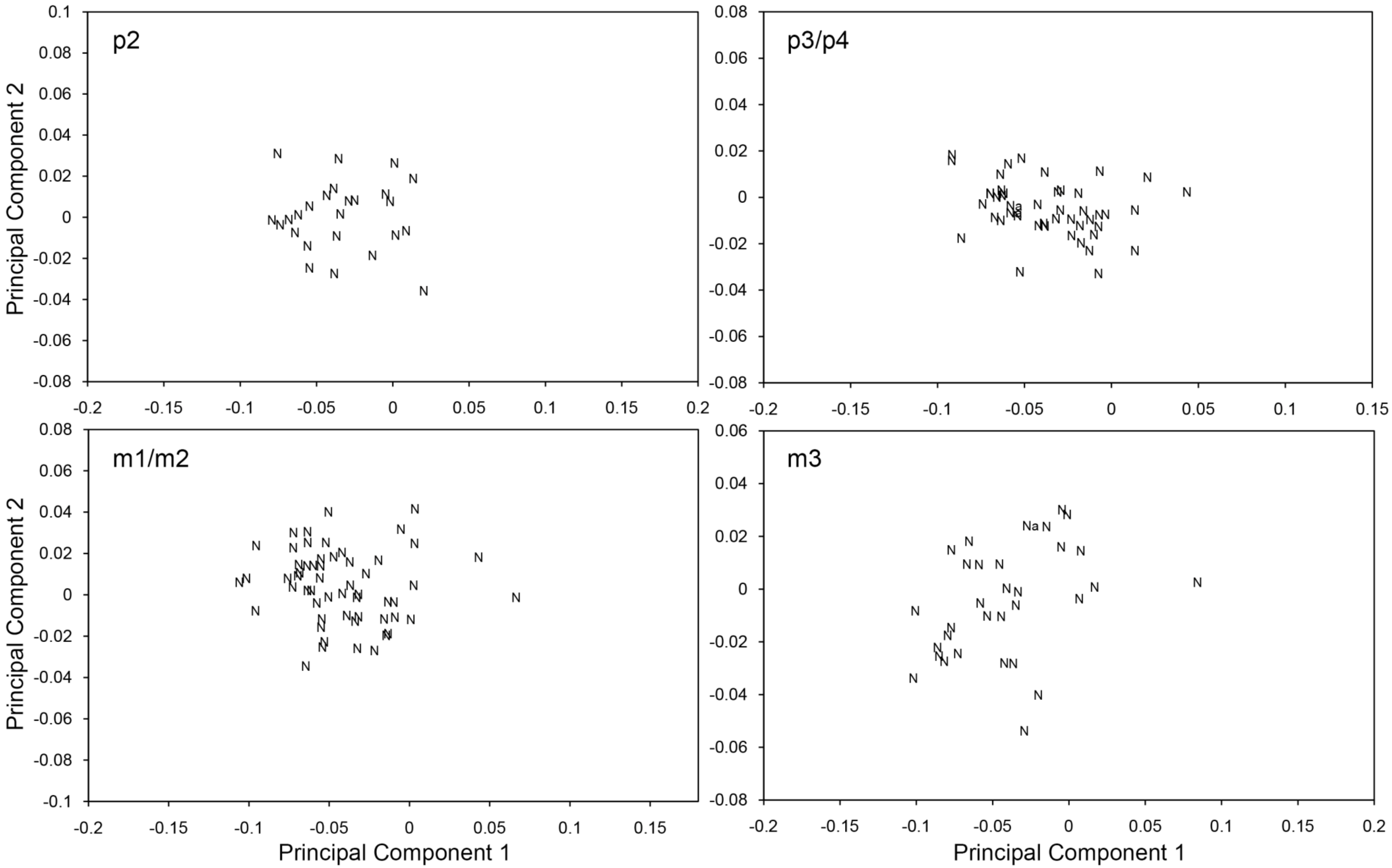

Figure F. Principal component plots showing lower teeth from Natural Trap Cave, Wyoming. Specimens that yielded aDNA (EQ13, EQ22, and EQ41) are identified by “a”.

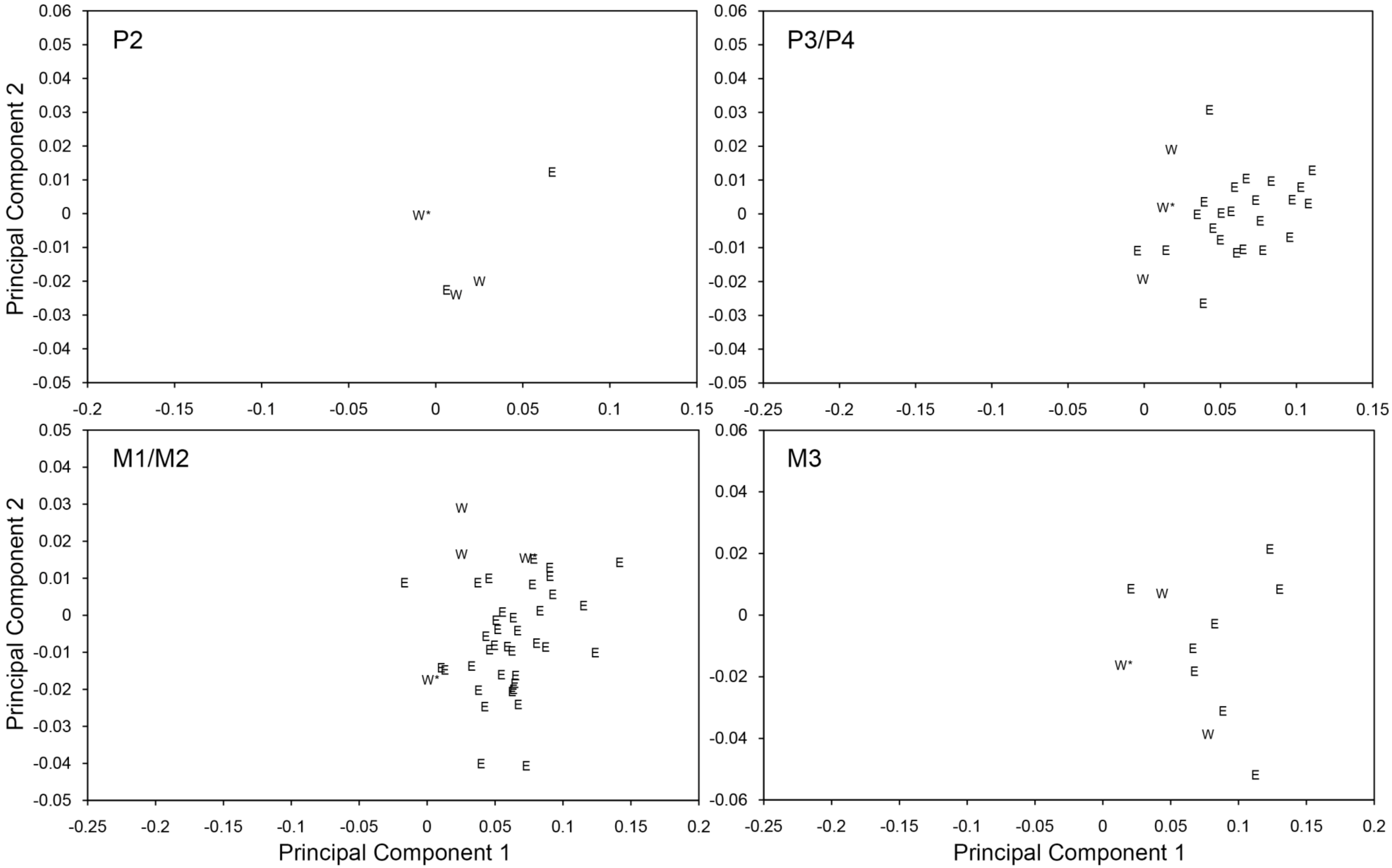

Figure G. Principal component plots showing upper teeth from the Edmonton area (E) and Wally's Beach (W), Alberta. Specimens associated with teeth that yielded aDNA (EQ43) are identified by "*".

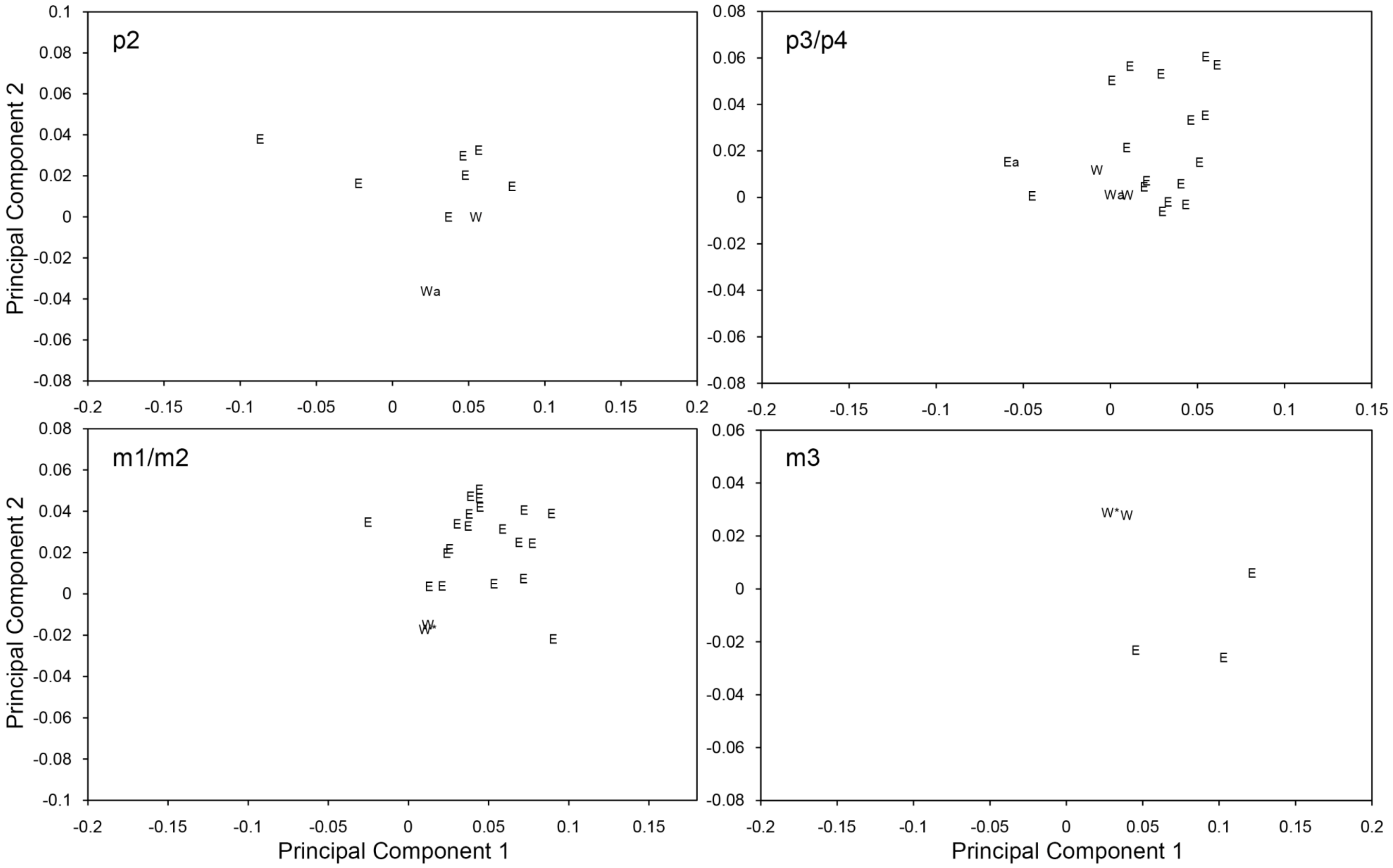

Figure H. Principal component plots showing lower teeth from the Edmonton area (E) and Wally's Beach (W), Alberta. Specimens that yielded aDNA (EQ4 and EQ43) and teeth associated with these specimens are identified by "a" and "*", respectively.

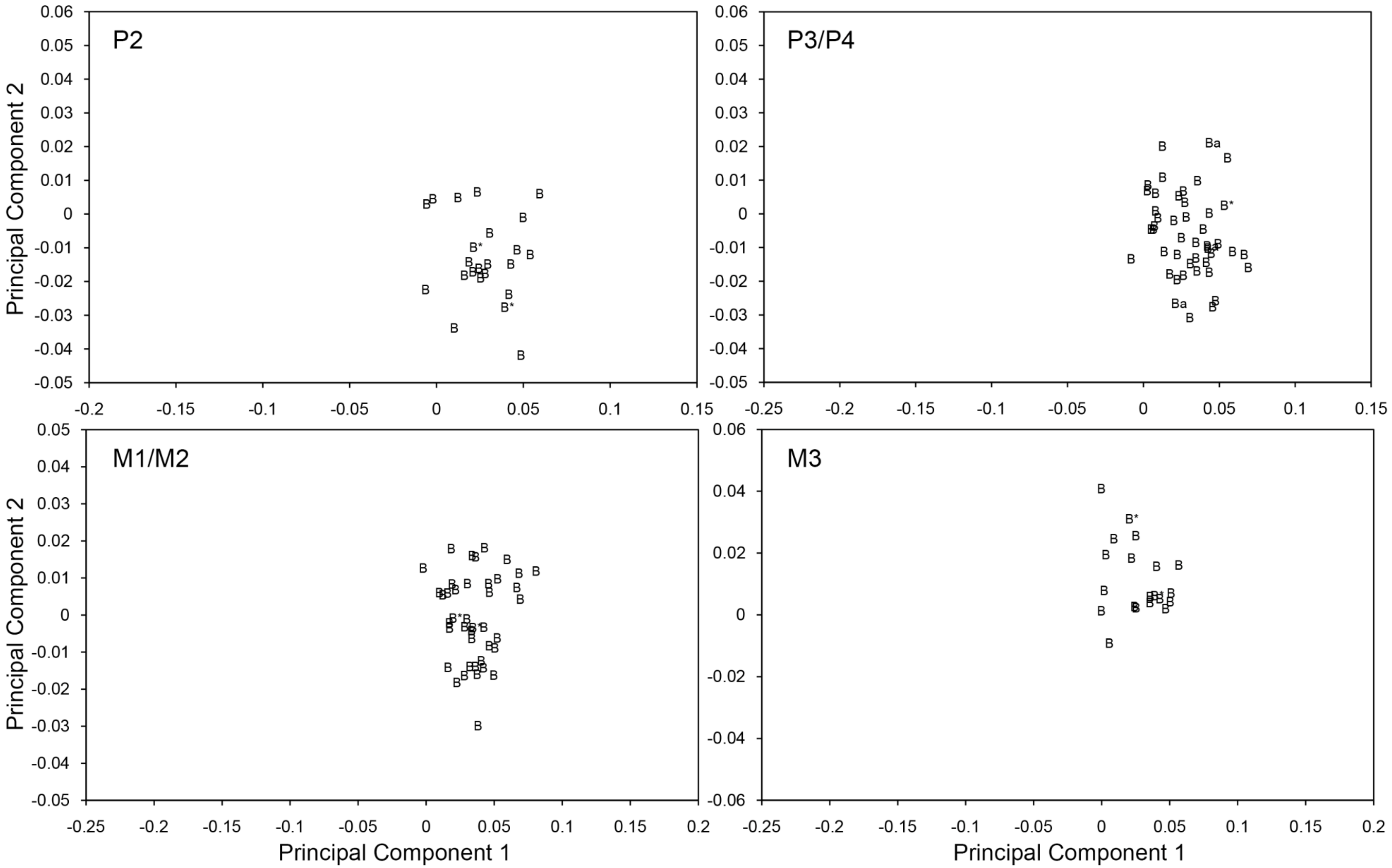

Figure I. Principal component plots showing upper teeth from Bluefish Caves, Yukon. Specimens that yielded aDNA (EQ38, EQ44, EQ45, and EQ47) and teeth associated with these specimens are identified by “a” and “*”, respectively.

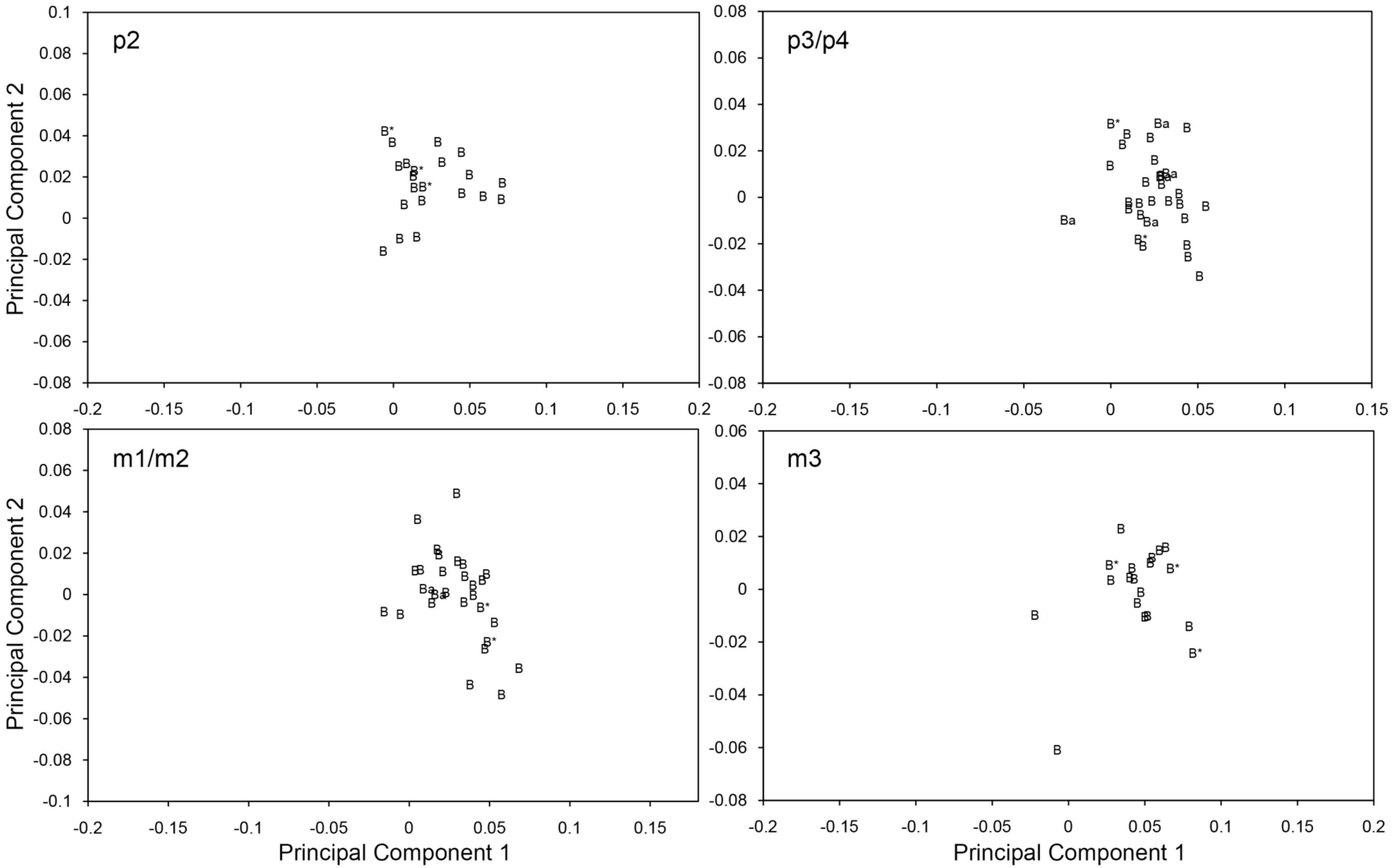

Figure J. Principal component plots showing lower teeth from Bluefish Caves, Yukon. Specimens that yielded aDNA (EQ39, EQ42, EQ47, EQ48, EQ50, EQ51, and EQ53) and teeth associated with these specimens are identified by “a” and “*”, respectively.

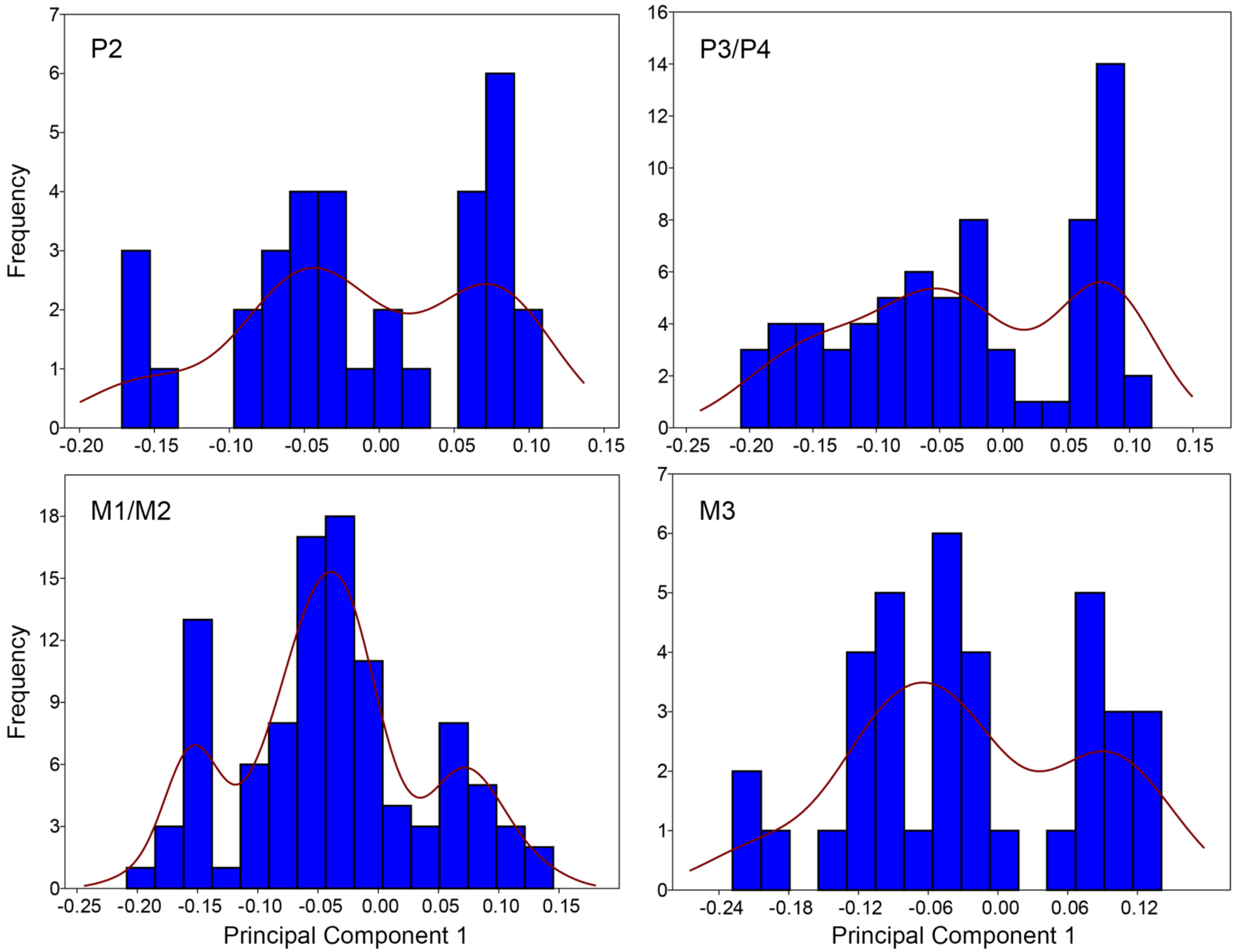

Figure K. Histograms showing the distribution of PC 1 scores of upper teeth from Cedral and San Josecito Cave, Mexico, resulting from PCA of the linear measurements (anteroposterior length and transverse width), taken at a crown height of 2 cm.

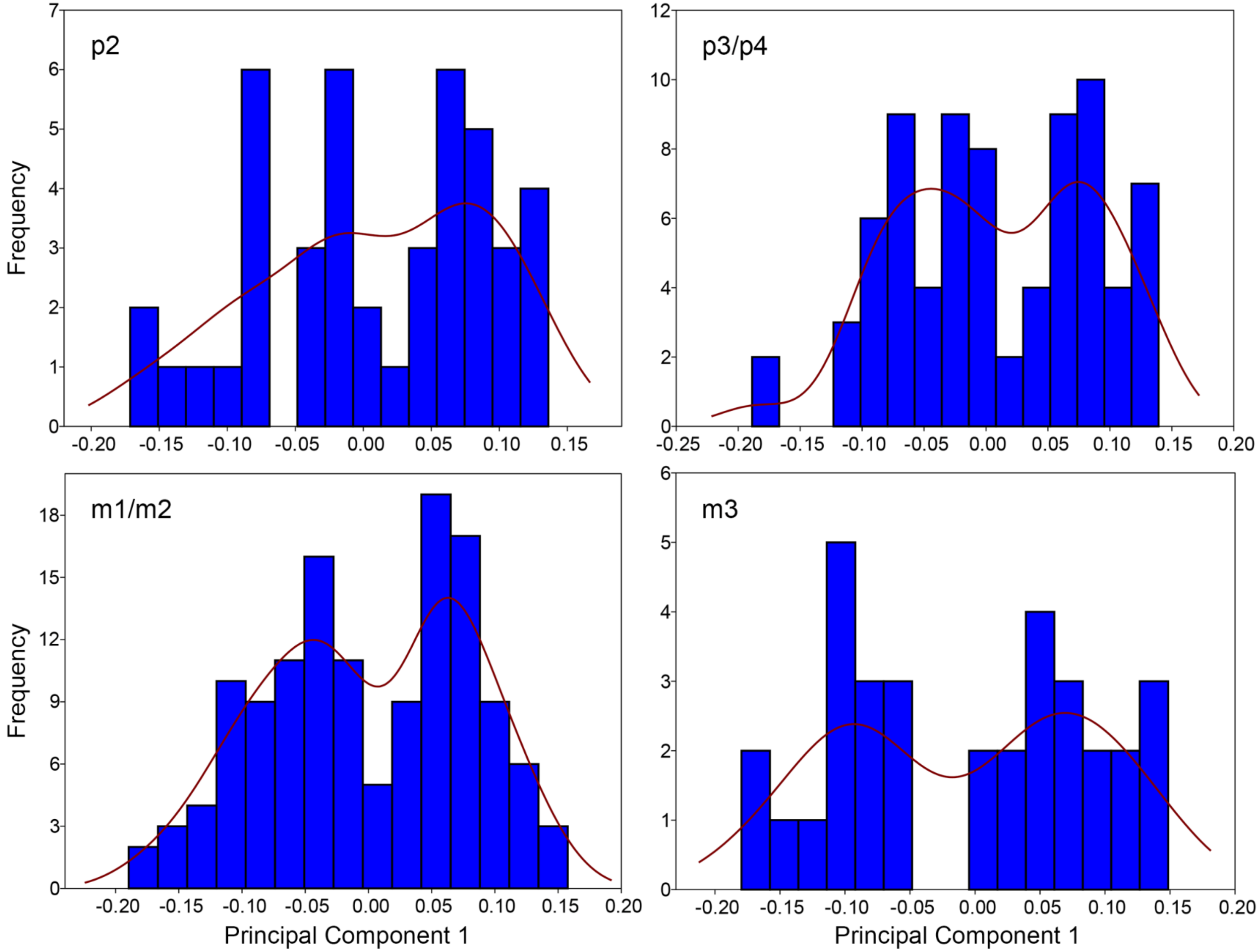

Figure L. Histograms showing the distribution of PC 1 scores of lower teeth from Cedral and San Josecito Cave, Mexico, resulting from PCA of the linear measurements (anteroposterior length and transverse width), taken at a crown height of 2 cm.

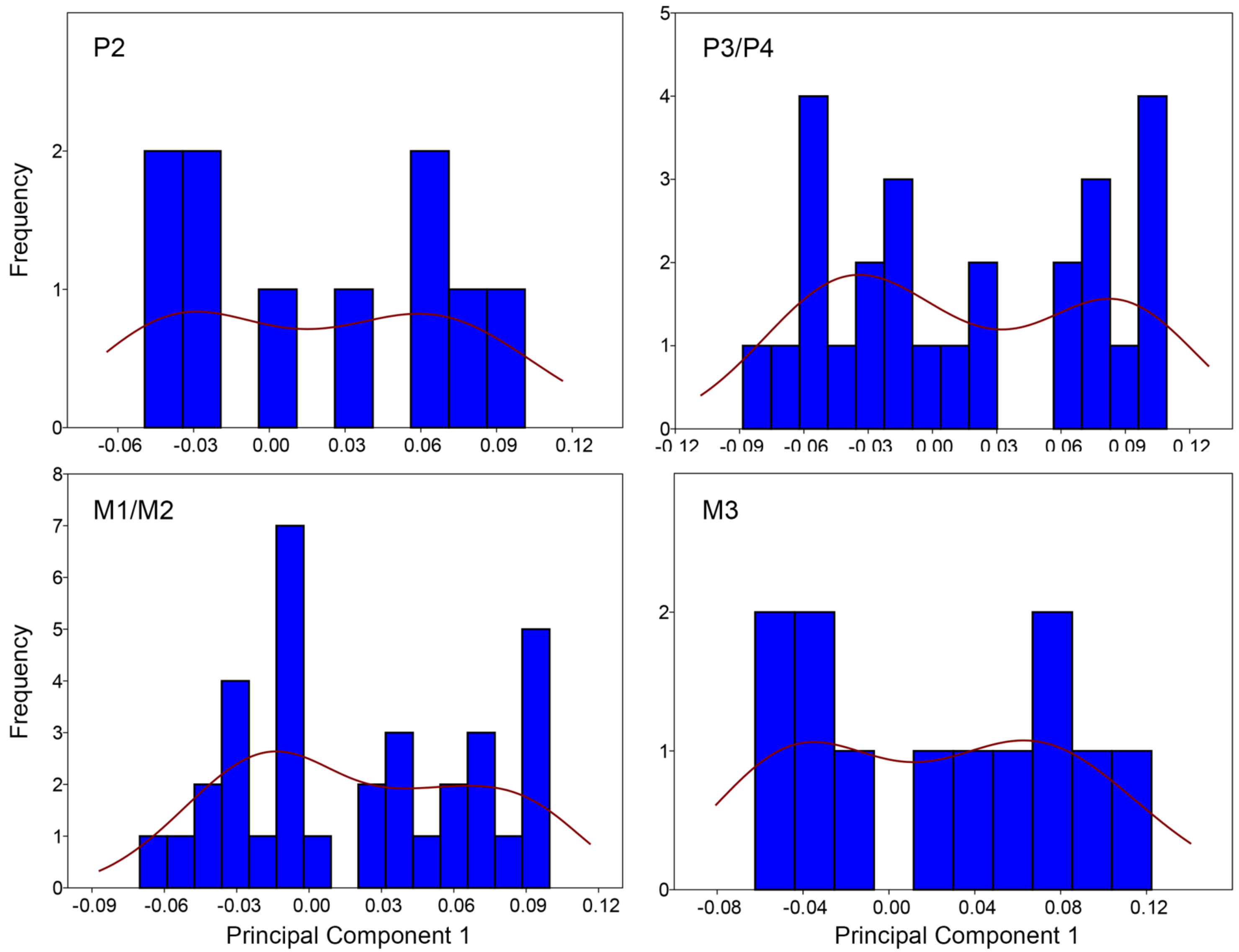

Figure M. Histograms showing the distribution of PC 1 scores of upper teeth from localities in the American Southwest (Figure 1), resulting from PCA of the linear measurements (anteroposterior length and transverse width), taken at a crown height of 2 cm.

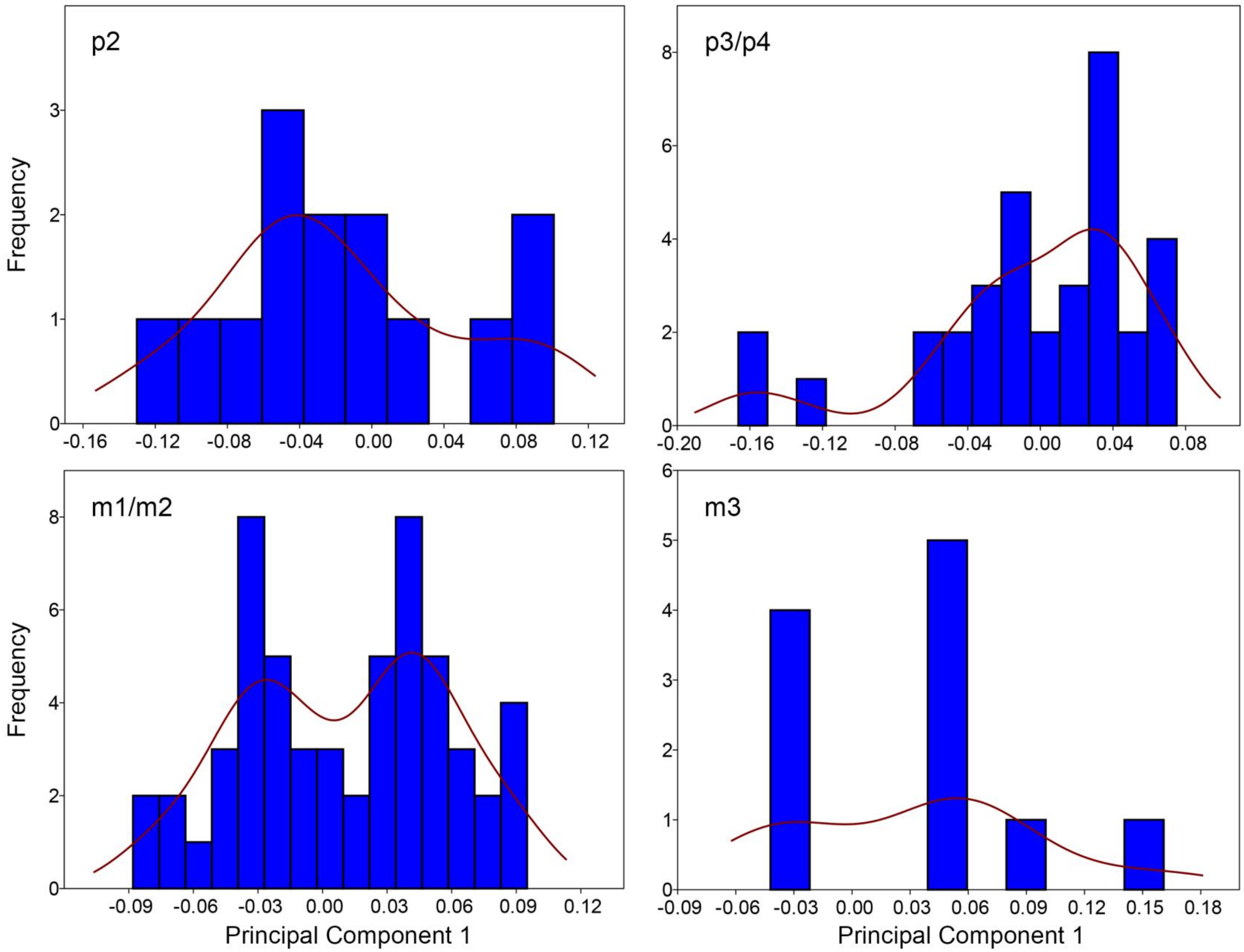

Figure N. Histograms showing the distribution of PC 1 scores of lower teeth from localities in the American Southwest (Figure 1), resulting from PCA of the linear measurements (anteroposterior length and transverse width), taken at a crown height of 2 cm.

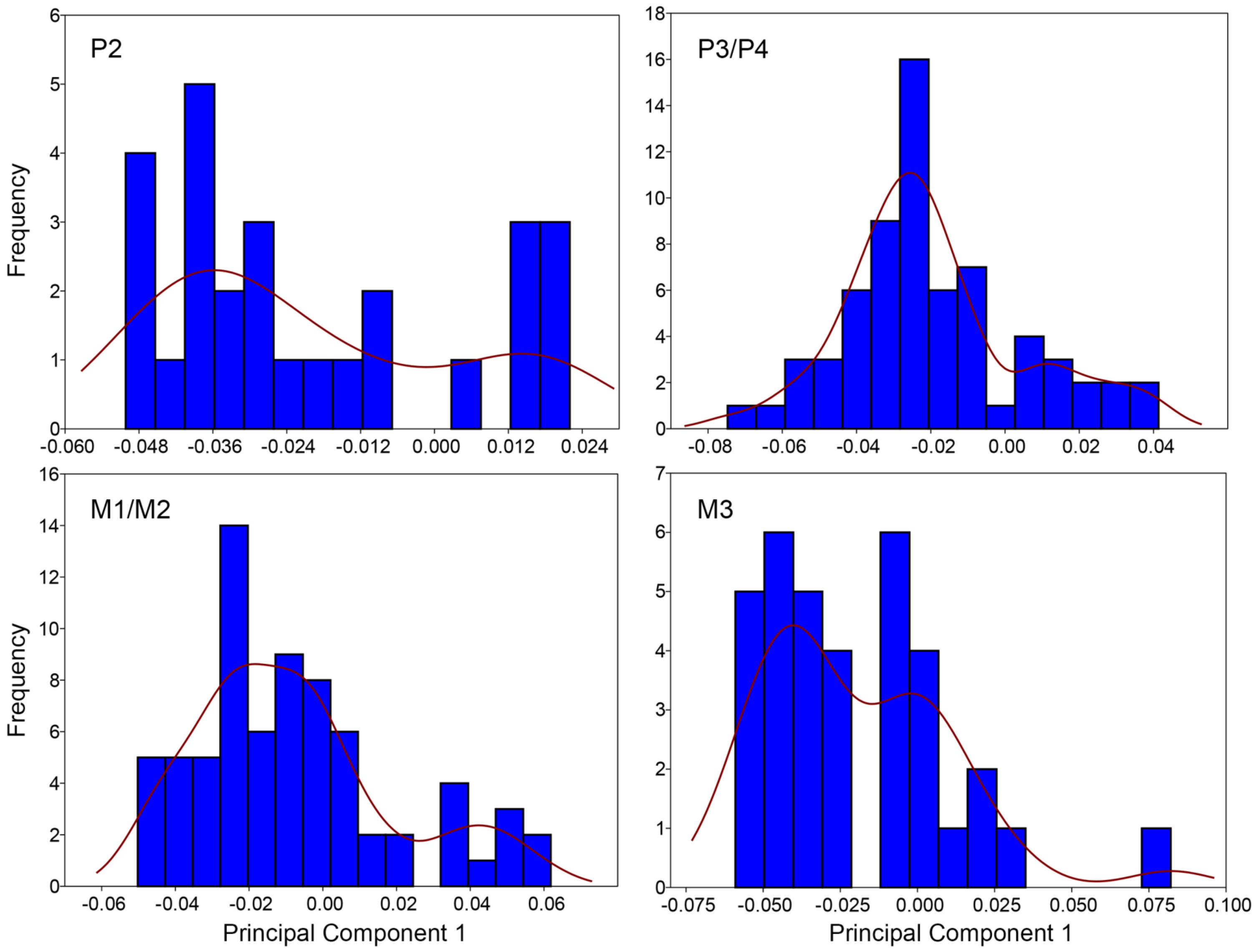

Figure O. Histograms showing the distribution of PC 1 scores of upper teeth from Natural Trap Cave, Wyoming, resulting from PCA of the linear measurements (anteroposterior length and transverse width), taken at a crown height of 2 cm.

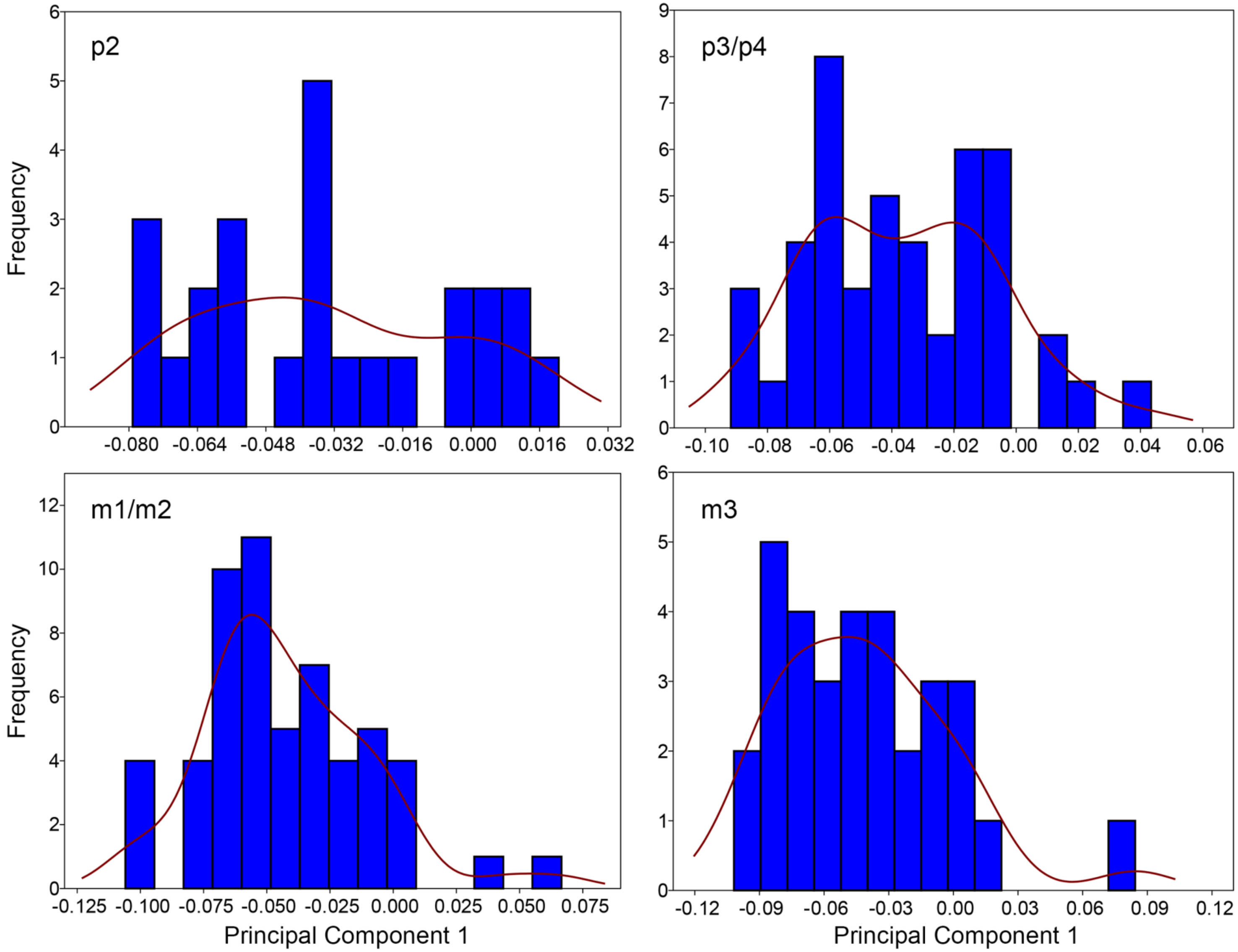

Figure P. Histograms showing the distribution of PC 1 scores of lower teeth from Natural Trap Cave, Wyoming, resulting from PCA of the linear measurements (anteroposterior length and transverse width), taken at a crown height of 2 cm.

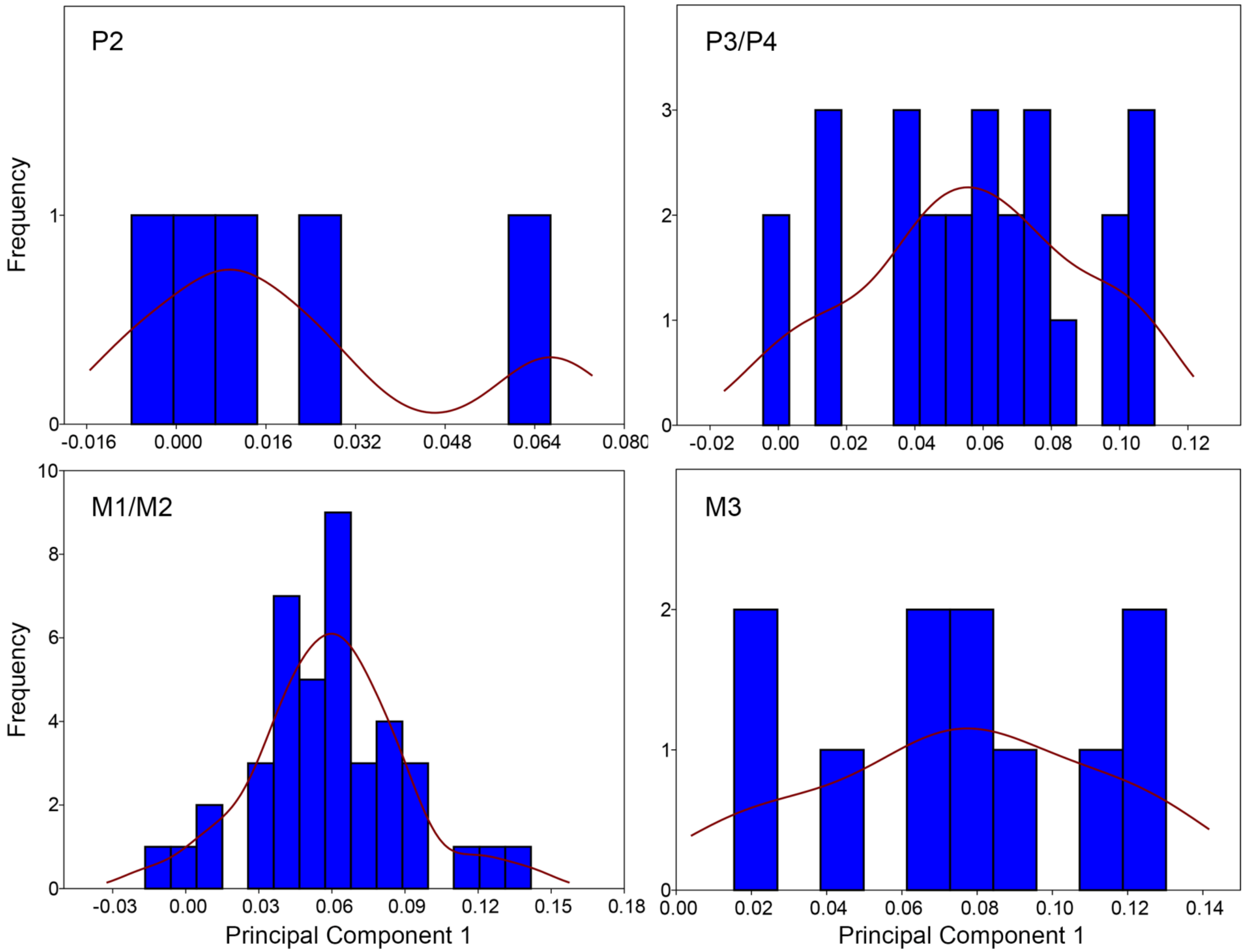

Figure Q. Histograms showing the distribution of PC 1 scores of upper teeth from the Edmonton area and Wally's Beach, Alberta, resulting from PCA of the linear measurements (anteroposterior length and transverse width), taken at a crown height of 2 cm.

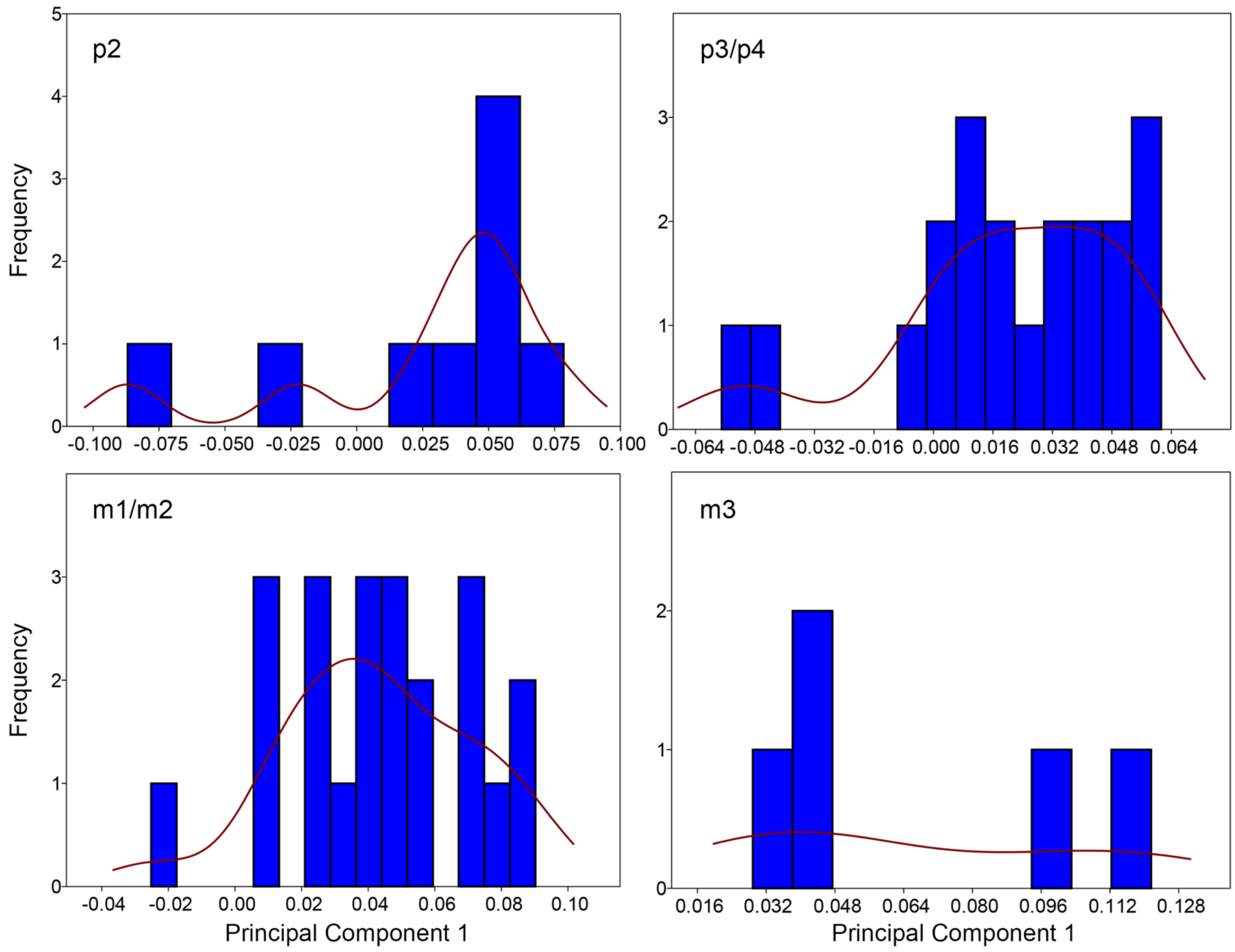

Figure R. Histograms showing the distribution of PC 1 scores of lower teeth from the Edmonton area and Wally's Beach, Alberta, resulting from PCA of the linear measurements (anteroposterior length and transverse width), taken at a crown height of 2 cm.

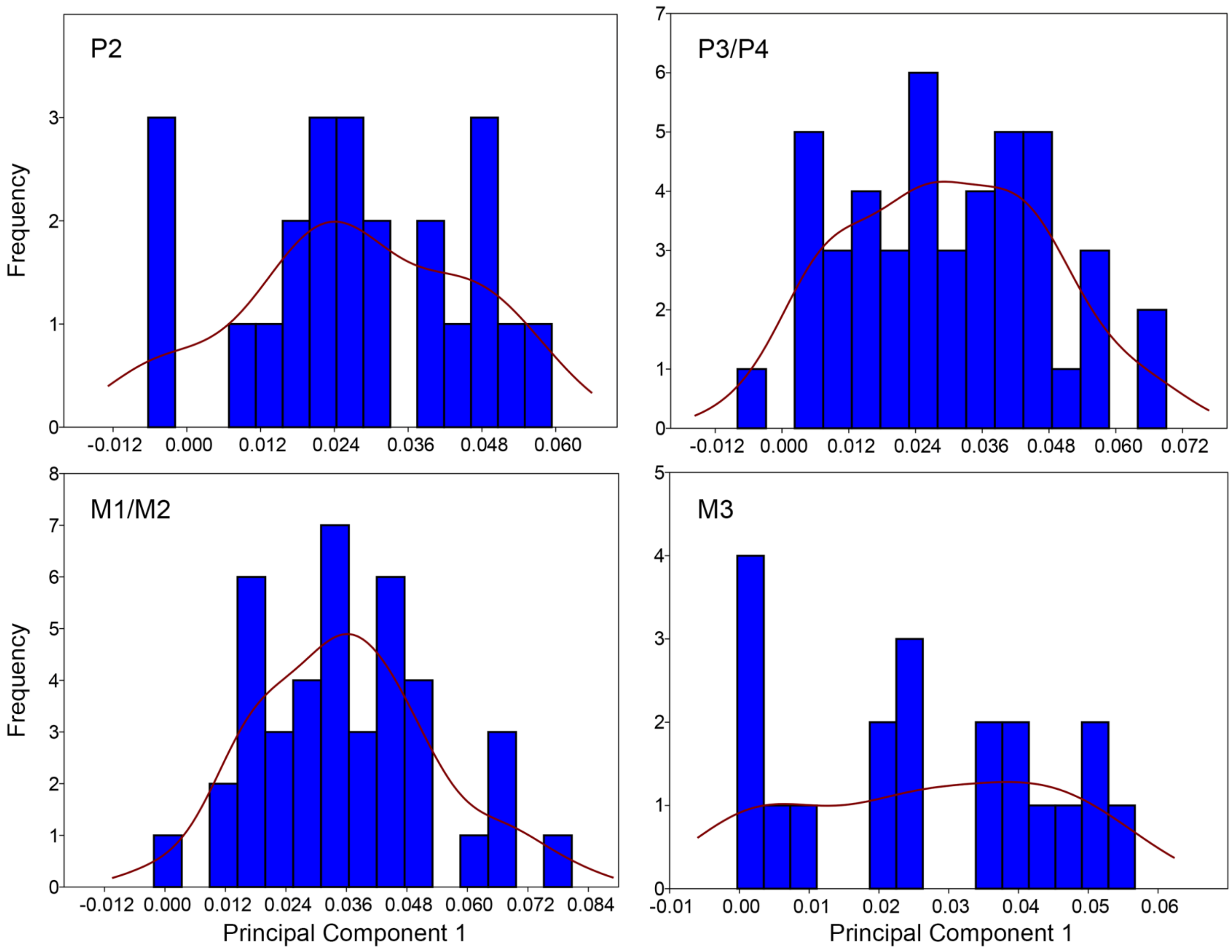

Figure S. Histograms showing the distribution of PC 1 scores of upper teeth from Bluefish Caves, Yukon, resulting from PCA of the linear measurements (anteroposterior length and transverse width), taken at a crown height of 2 cm.

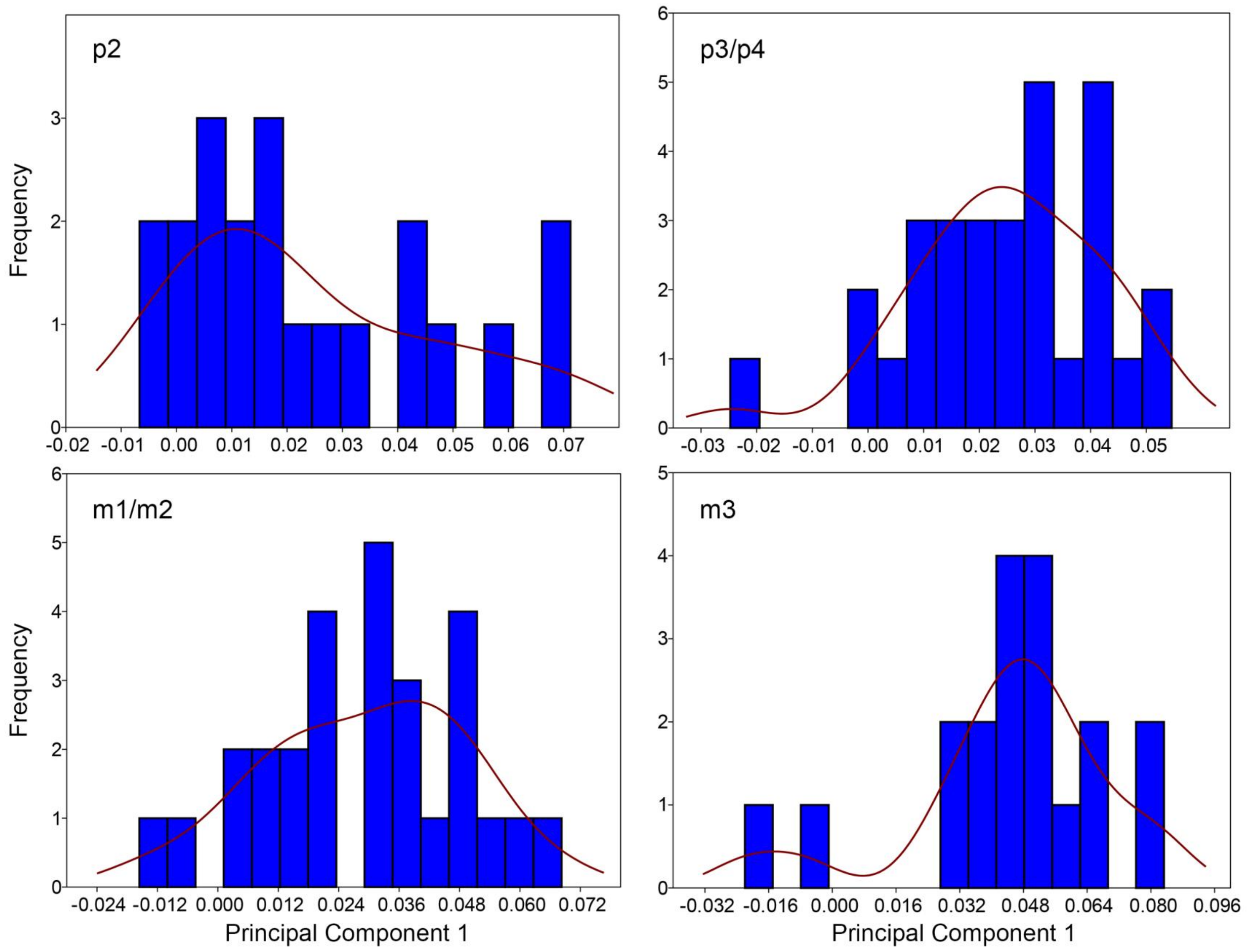

Figure T. Histograms showing the distribution of PC 1 scores of lower teeth from Bluefish Caves, Yukon, resulting from PCA of the linear measurements (anteroposterior length and transverse width), taken at a crown height of 2 cm.

Table A. Late Pleistocene equid specimens (upper teeth) included in the geometric morphometric analysis. Loc. ab. = Abbreviation of locality names shown in Figures 7 and 8 of the main text (l = large, m = medium, s = small specimens; teeth that yielded aDNA are identified by “a” and those associated with specimens from which aDNA was obtained are indicated with “*”).

Specimen	Locality	Loc. ab.	Tooth position
DP 2584	Cedral	Cl	P4l
TMM 41228-1051	Dark Canyon Cave	Km	P3r
MgVo-2 H5-3-24	MgVo-2	B	P3r
LACM 192/120751	San Josecito Cave	J	P3r
P94.1.498	Pit 48	E	P4r
UTEP 34-8	Salt Creek	Sm	P4l
MgVo-1 I7-1-21	MgVo-1	B	P3r
LACM 192/156487	San Josecito Cave	J	P3/P4r
MgVo-1 K8-1-8	MgVo-1	B	P3l
DP 2608	Cedral	Cl	P4r
TMM 937-253	Blackwater Draw	Ll	P4l
DP 2606	Cedral	Cl	P3r
KU 38192	Natural Trap Cave	N	P4l
KU 52450	Natural Trap Cave	N	P4r
LACM 192/18105	San Josecito Cave	J	P4r
MgVo-1 L7-7-3	MgVo-1	B	P3r
P02.10.125	Pit 48	E	P3l
KU 36548	Natural Trap Cave	N	P3r
LACM 192/159131	San Josecito Cave	J	P3l
MgVo-1 E7-19-1	MgVo-1	B	P3r
DP 3838	Cedral	Cl	P3r
KU 62586	Natural Trap Cave	N	P3r
LACM 192/156497	San Josecito Cave	J	P4r
TMM 41228-360	Dark Canyon Cave	Km	P4r
DP 4587	Cedral	Cs	P3r
UTEP 46-139	Isleta Cave No. 2	Il	P3l
LACM 192/18109	San Josecito Cave	J	P3r
UTEP 112-2	Nash Draw	Hl	P4r
KU 32315	Natural Trap Cave	N	p4l
LACM 192/18111	San Josecito Cave	J	P4r
DP 2604	Cedral	Cl	P4l
LACM 192/159126	San Josecito Cave	J	P4r
MgVo-3 85-120	MgVo-3	B	P4l
KU 27214	Natural Trap Cave	N	P3r
KU 32789	Natural Trap Cave	N	P3/P4r
P89.13.397	Pit 48	E	P4/P3l
KU 36614	Natural Trap Cave	N	P3l
P98.5.21	Pit 48	E	P3r
KU 36456	Natural Trap Cave	Nl	P3l

DP 3850	Cedral	Cm	P3r
DP 2572	Cedral	Cm	P4r
LACM 192/156496	San Josecito Cave	J	P3r
MgVo-1 I7-1-48	MgVo-1	B	P3l
KU 36748	Natural Trap Cave	N	P4l
DP 3836	Cedral	Cm	P3r
MgVo-1 G7(E1/2)-14-3[1]	MgVo-1	B*	P3l
UTEP 119-14	Algerita Blossom Cave	Am	P3l
MgVo-1 K8-1-15	MgVo-1	B	P3r
DP 3847	Cedral	Cl	P4l
KU 41984	Natural Trap Cave	Nl	P4r
DP 2662	Cedral	Cl	P3l
TMM 937-504.1	Blackwater Draw	Lm	P3/P4r
KU 46645	Natural Trap Cave	N	P3l
MgVo-1 H7(E)-21-2	MgVo-1	B	P4l
DP 2607	Cedral	Cl	P4r
LACM 192/159127	San Josecito Cave	J	P3l
P94.1.282	Pit 48	E	P3r
MgVo-1 H7(E)-14-20	MgVo-1	B	P4l
MgVo-2 85-Misc-1.5	MgVo-2	B	P4r
KU 32985	Natural Trap Cave	N	P3r
DP 3842	Cedral	Cs	P4l
MgVo-1 K7-5-22	MgVo-1	B	P3l
P83.4.2	Alberta Concrete Products Gravel Pit	E	P3/P4l
KU 38688	Natural Trap Cave	N	P3l
P94.1.557	Pit 48	E	P3r
P94.4.6	Pit 46	E	P4l
DP 3836	Cedral	Cs	P4l
KU 39232	Natural Trap Cave	N	P3r
DhPg-8 3437.1 (Horse 2)[2]	Wally's Beach	W*	P3r
KU 34020	Natural Trap Cave	N	P4r
P89.13.400	Pit 48	E	P4l
KU 35769	Natural Trap Cave	N	P3r
KU 48366	Natural Trap Cave	N	P4r
MgVo-1 G6-4-4	MgVo-1	B	P3l
KU 81897	Natural Trap Cave	N	P3l
DP 4554	Cedral	Cm	P3r
DP 2593	Cedral	Cm	P4r
MgVo-1 H8(N)-8-6	MgVo-1	B	P4r
P94.1.470	Pit 48	E	P3l
DP 3840	Cedral	Cs	P4l
KU 39803	Natural Trap Cave	N	P3r
TMM 998-25	Scharbauer Ranch	Rl	P3/P4r
KU 42970[3]	Natural Trap Cave	N*	P3l

DP 3862	Cedral	Cs	P3r
DP 33219	Natural Trap Cave	N	P3l
DP 2638	Cedral	Cm	P4l
MgVo-2 (D3)-6-7	MgVo-2	B	P3l
MgVo-3 T.P.1-5	MgVo-3	B	P3r
P89.13.610	Pit 48	E	P4r
KU 38555	Natural Trap Cave	N	P3r
TMM 937-253	Blackwater Draw	Ll	P4l
KU 48342	Natural Trap Cave	N	P4l
MgVo-1 J8-1-166	MgVo-1	B	P4l
LACM 192/156487	San Josecito Cave	J	P3/P4r
DhPg-8 860.1 (Horse 3)	Wally's Beach	W	P4r
DP 2595	Cedral	Cl	P3r
UTEP 54-1212	TTII (Dry Cave)	Dm	P3r
DP 4548	Cedral	Cs	P3r
LACM 192/156483	San Josecito Cave	J	P3l
KU 44396	Natural Trap Cave	N	P3r
DP 3863	Cedral	Cs	P3l
KU 81894	Natural Trap Cave	N	P4/P3r
DhPg-8 862 (Horse A)	Wally's Beach	W	P4r
TMM 937-678	Blackwater Draw	Ll	P3r
DP 3854	Cedral	Cs	P4l
KU 34084	Natural Trap Cave	N	P4l
DP 2652	Cedral	Cm	P3?/P4l
MgVo-3 85-89	MgVo-3	Ba	P3l
UTEP 22-1608	Animal Fair (Dry Cave)	Dl	P3l
MgVo-1 T3-21-79[4]	MgVo-1	B*	P4r
KU 27215	Natural Trap Cave	N	P3r
KU 48719	Natural Trap Cave	N	P3l
P94.1.242	Pit 48	E	P3r
MgVo-1 H7(W)-3-36	MgVo-1	Ba	P3r
LACM 192/116874	San Josecito Cave	J	P3l
KU 35085	Natural Trap Cave	N	P3l
MgVo-2 D6-D-4	MgVo-2	B	P3r
MgVo-2 B4(S)-12-8	MgVo-2	B	P3l
DP 4547	Cedral	Cm	P4l
TMM 937-923	Blackwater Draw	Lm	P4r
LACM 192/18104	San Josecito Cave	J	P3l
KU 41178	Natural Trap Cave	N	P3l
MgVo-1 K6-2-23	MgVo-1	B	P3l
MgVo-1 K7-1-6	MgVo-1	B	P3l
UTEP 22-1609[5]	Animal Fair (Dry Cave)	Dm*	P3l
P98.5.234	Pit 48	E	P4l
LACM 192/156484	San Josecito Cave	J	P4r
KU 45751	Natural Trap Cave	N	P3r

DP 2576	Cedral	Cl	P4r
P99.3.6	Pit 48	E	P4?l
P94.1.259	Pit 48	E	P3/P4l
DP 3868	Cedral	Cs	P4r
DP 4540	Cedral	Cm	P3l
MgVo-3 85-41	MgVo-3	B	P3r
KU 27902	Natural Trap Cave	N	P4l
KU 32785	Natural Trap Cave	N	P3r
MgVo-1 J7-1-12	MgVo-1	B	P3l
UTEP 25-537	Camel Room (Dry Cave)	Dm	P3r
KU 36438	Natural Trap Cave	N	P3r
MgVo-2 C6(W)-13-1	MgVo-2	B	P4l
KU 38612	Natural Trap Cave	N	P3r
KU 46778	Natural Trap Cave	N	P3l
DP 2651	Cedral	Cm	P3l
LACM 192/156486	San Josecito Cave	J	P4l

[1]: aDNA obtained from associated P3l: MgVo-1 G7(E1/2)-11-13
[2]: aDNA obtained from associated p2r and p3l: DhPg-8 3437.2
[3]: aDNA obtained from associated P2l: KU 42970
[4]: aDNA obtained from associated m2r: MgVo-1 T3-21-86
[5]: aDNA obtained from associated P4l: UTEP 22-1609

Table B. Late Pleistocene equid specimens (lower teeth) included in the geometric morphometric analysis. Loc. ab. = Abbreviation of locality names shown in Figures 9 and 10 of the main text (l = large, m = medium, s = small specimens; teeth that yielded aDNA are identified by "a" and those associated with specimens from which aDNA was obtained are indicated with "*").

Specimen	Locality	Loc. ab.	Tooth position
KU 36625	Natural Trap Cave	N	p4l
DP 2614	Cedral	Cl	p4l
LACM 192/156491	San Josecito Cave	J	p4r
DP 4562	Cedral	Cm	p3r
TMM 41228-393	Dark Canyon Cave	Km	p3/p4l
P98.5.484	Pit 48	E	p3/p4l
P91.11.2	Pit 46	E	p4r
MgVo-2 C3(E)-2-37	Bluefish Cave 2	B	p3r
LACM 192/156494	San Josecito Cave	J	p4l
TMM 937-961	Blackwater Draw	Ll	p3/p4l
DhPg-8 3437.1 (Horse 2)	Wally's Beach	Wa	p3l
TMM 937-169	Blackwater Draw	Ll	p4r
KU 35082	Natural Trap Cave	N	p4l
KU 27216	Natural Trap Cave	N	p4r
MgVo-2 C3(E)-3-2[1]	Bluefish Cave 2	B*	p4l
UTEP 22-669	Animal Fair (Dry Cave)	Dm	p4r
KU 36626	Natural Trap Cave	N	p3r
DP 2687	Cedral	Cl	p3r
LACM 192/159153	San Josecito Cave	J	p4l
LACM 192/156492	San Josecito Cave	J	p4l
DP 4575	Cedral	Cm	p3r
UTEP 22-1528	Charlies Parlor (Dry Cave)	Dl	p4r
TMM 41228-389	Dark Canyon Cave	Km	p4r
DhPg-8 69.1 (Horse D)	Wally's Beach	W	p4r
LACM 192/159156	San Josecito Cave	J	p4l
MgVo-1 J8-1-147	Bluefish Cave 1	Ba	p3l
LACM 192/156495	San Josecito Cave	J	p4r
DP 2592	Cedral	Cl	p3r
UTEP 22-1538	Charlies Parlor (Dry Cave)	Dl	p4r
P94.1.386	Pit 48	E	p4r
KU 40835	Natural Trap Cave	N	p3/p4r
DP 2688	Cedral	Cl	p4l
LACM 192/156493	San Josecito Cave	J	p3r
TMM 8106	Quitaque Creek	Qm	p4r
DP 4577	Cedral	Cm	p3/p4r
TMM 41228-386	Dark Canyon Cave	Km	p3r
MgVo-1 J7-C-16	Bluefish Cave 1	B	p3l
DhPg-8 861.1 (Horse 3)	Wally's Beach	W	p4l
MgVo-2 J7-8.19[2]	Bluefish Cave 2	B*	p3l

MgVo-1 L8(N)-4-7	Bluefish Cave 1	Ba	p3r
TMM 937-244	Blackwater Draw	Ll	p4l
KU 34344	Natural Trap Cave	N	p3/p4l
UTEP 54-1312	TTII (Dry Cave)	Dl	p4l
KU 35114	Natural Trap Cave	N	p3/p4l
KU 33729	Natural Trap Cave	N	p4l
KU 36624	Natural Trap Cave	N	p3/p4r
TMM 937-949	Blackwater Draw	Ll	p3l
MgVo-2 H6-3-8	Bluefish Cave 2	B	p3l
UTEP 22-1615	Charlies Parlor (Dry Cave)	Dl	p4/p3r
DP 2591	Cedral	Cl	p3l
KU 41710	Natural Trap Cave	N	p3/p4l
DP 2707	Cedral	Cm	p3l
KU 36543	Natural Trap Cave	Na	p3r
MgVo-3 S-3-98	Bluefish Cave 3	B	p3l
P94.1.499	Pit 48	Ema	p3l
MgVo-1 L8(N)-7-15	Bluefish Cave 1	B	p4l
TMM 937-250	Blackwater Draw	Ll	p3l
TMM 937-973	Blackwater Draw	Lm	p4l
UTEP 23-65	Stalag 17 (Dry Cave)	Dma	p3r
MgVo-3 85-90[3]	Bluefish Cave 3	B*	p4r
DP 2698	Cedral	Cl	p4l
KU 31434	Natural Trap Cave	N	p3/p4l
DP 2708	Cedral	Cm	p3r
LACM 192/159157	San Josecito Cave	J	p3l
MgVo-2 I6-E-5	Bluefish Cave 2	Ba	p4r
P94.1.585	Pit 48	E	p3r
LACM 192/8543	San Josecito Cave	J	p3l
LACM 192/120844	San Josecito Cave	J	p3l
TMM 998-9	Scharbauer Ranch	Rm	p3r
MgVo-3 S-3-94	Bluefish Cave 3	B	p4r
TMM 937-251	Blackwater Draw	Ll	p4l
UTEP 25-537	Camel Room (Dry Cave)	Dm	p3r
DP 2554	Cedral	Cl	p3r
DP 2709	Cedral	Cs	p3r
MgVo-1 K8-2-3	Bluefish Cave 1	B	p4l
DP 4601	Cedral	Cl	p4r
MgVo-3 85-95	Bluefish Cave 3	B	p4r
MgVo-1 J8-1-145	Bluefish Cave 1	B	p4l
LACM 192/159158	San Josecito Cave	J	p3l
P94.1.632	Pit 48	E	p4l
TMM 937-725	Blackwater Draw	Ll	p4r
UTEP 75-31	Dark Canyon Cave	Km	p3r
TMM 937-954	Blackwater Draw	Ll	p4r
DP 2706	Cedral	Cs	p3l

TMM 41228-3821.2	Dark Canyon Cave	Km	p3r
LACM 192/156488	San Josecito Cave	J	p3l
TMM 937-933	Blackwater Draw	Ll	p3/p4l
KU 32587	Natural Trap Cave	N	p4l
KU 50629	Natural Trap Cave	N	p4r
KU 54348	Natural Trap Cave	N	p4r
LACM 192/159155	San Josecito Cave	J	p4r
P89.13.50	Pit 48	E	p3/p4r
KU 33867	Natural Trap Cave	Na	p3/p4l
MgVo-1 L8(N)-7-2	Bluefish Cave 1	B	p3l
UTEP 203-1	Highway 45, Chihuahua	Gs	p3l
UTEP 189-4	Villa Ahumada	Vs	p3/p4r
DP 3929	Cedral	Cs	p4r
LACM 192/159154	San Josecito Cave	J	p4r
DP 3892	Cedral	Cs	p3r
P89.13.7	Pit 48	E	p3r
P99.3.162	Pit 48	E	p3l
MgVo-3 85-64	Bluefish Cave 3	B	p3r
TMM 937-965	Blackwater Draw	Ll	p3/p4l
LACM 192/156489	San Josecito Cave	J	p4l
TMM 937-940	Blackwater Draw	Ll	p4l
DP 2633	Cedral	Cl	p3r
KU 41592	Natural Trap Cave	N	p3l
P89.13.620	Pit 48	E	p4r
UTEP 189-5	Villa Ahumada	Vs	p3/p4r
KU 41983	Natural Trap Cave	N	p3l
DP 4559	Cedral	Cm	p4?l
KU 27390	Natural Trap Cave	Nl	p4l
KU 36158	Natural Trap Cave	N	p4r
LACM 192/156490	San Josecito Cave	J	p3l
P98.5.480	Pit 48	Em	p3l
TMM 937-972	Blackwater Draw	Lm	p3/p4r
TMM 937-945	Blackwater Draw	Ll	p3l
TMM 937-122	Blackwater Draw	Lm	p4l
P97.11.2A	Riverview Pit	E	p3r
MgVo-1 D6(NE)-8-14	Bluefish Cave 1	B	p3l
P94.1.486	Pit 48	E	p3l
DP 2649	Cedral	Cm	p3r
DP 2628	Cedral	Cm	p4r
DP 2312	Cedral	Cm	p3l
DP 2684	Cedral	Cl	p3/p4r
DP 2645	Cedral	Cl	p3r
DP 3909	Cedral	Cl	p3r
DP 4569	Cedral	Cl	p4r

[1]: aDNA obtained from associated m2r: MgVo-2 B3-3-16
[2]: aDNA obtained from associated p4l: MgVo-2 J7-8.19
[3]: aDNA obtained from associated p3r: MgVo-3 85-90

Table C. Landmarks used in the geometric morphometric analysis of the upper P3/P4 teeth.

Landmark	Location
1	Metastyle
2	Posterior point of mesostyle
3	Anterior point of mesostyle
4	Start of mesostyle-parastyle valley
5	Posterior point of parastyle
6	Anterior point of parastyle
7	Pre-protoconal groove
8	Anterior point of protocone
9	Posterior point of protocone
10	Post-protoconal groove
11	Buccal extension of post-protoconal valley
12	Hypocone
13	Posterior enamel loop of postfossette
14	Pli postfossette
15	Anterior enamel loop of postfossette
16	Posterior enamel loop of prefossette
17	Pli paraconule
18	Anterior enamel loop of prefossette
19	Metastyle-mesostyle valley
20	Buccal enamel band of postfossette
21	Lingual enamel band of postfossette
22	Mesostyle-parastyle valley
23	Buccal enamel band of prefossette
24	Lingual enamel band of prefossette

Landmarks 1 to 18 are type II landmarks. Landmarks 19 to 24 are type III landmarks (defined by their relative position to other landmarks). Landmark 19 is placed at the mid-point of landmarks 1 and 2 and at the intersection with the enamel band of the metastyle-mesostyle valley. Landmarks 20 and 21 are placed at the mid-point of landmarks 1 and 2 and at the intersection with the buccal and lingual enamel bands of the postfossette, respectively, following the orientation of the posterior side of the tooth (i.e., landmarks 19, 20, and 21 are parallel to the posterior side of the tooth). Landmark 22 is placed at the mid-point of landmarks 3 and 5 and at the intersection with the enamel band of the mesostyle-parastyle valley. Landmarks 23 and 24 are placed at the mid-point of landmarks 3 and 5 and at the intersection with the buccal and lingual enamel bands of the prefossette, respectively, following the orientation of the posterior side of the tooth (i.e., landmarks 22, 23, and 24 are parallel to the posterior side of the tooth).

Table D. Equid specimens that were sampled for ancient mtDNA. * indicates a specimen that yielded aDNA sequence data.

Sample no.	Specimen	Locality	Element	GenBank #
EQ1*<br>(ind. replicated)	UTEP 23-65	Dry Cave, New Mexico	Lower p3r	KX137124
EQ2*<br>(ind. replicated)	UTEP 22-648	Dry Cave, New Mexico	Lower m1l	KX137125
EQ3*/EQ18	UTEP 22-1609	Dry Cave, New Mexico	Upper P4l	KX137126
EQ4*	P94.1.499	Pit 48, Alberta	Lower p3l	KX137127
EQ5	P94.1.486	Pit 48, Alberta	Lower p3l	
EQ6*/EQ40*<br>(repeat)	U of C Historic Horse,	Calgary, Alberta	Rib	
EQ7	DP 2315	Cedral, Mexico	Lower p4r	
EQ8	UTEP 22-1539	Dry Cave, New Mexico	Lower p3/p4	
EQ9*<br>(ind. replicated)	KU 42970	Natural Trap Cave, WY	Upper P2l	KX137128
EQ10	DP L-12, VII, 152	Loltun, Mexico	Lower p3/p4	
EQ11	DP 4586	Cedral, Mexico	Lower m2l	
EQ12	UTEP 22-65	Dry Cave, New Mexico	Upper M3l	
EQ13*	KU 33867	Natural Trap Cave, WY	Lower p3/p4l	KX137129
EQ14	UTEP 75-29	Dark Canyon Cave, NM	Upper M2r	
EQ15	DP 2676	Cedral, Mexico	Upper M2r	
EQ16*	UTEP 4-827	Dry Cave, New Mexico	Upper M1r	KX137130<br>KX137131
EQ17	UTEP 46-139	Isleta Cave No. 2, NM	Upper P3l	
EQ19	DP 4587	Cedral, Mexico	Lower m3r	
EQ20	UTEP 22-1615/22-1616?	Dry Cave, New Mexico	Lower p4?r	
EQ21	TMM 41228-250	Dark Canyon Cave, NM	Lower m1r	
EQ22*	KU 36543	Natural Trap Cave, WY	Lower p3r	KX137132
EQ23	UTEP 5689-67-6	U-Bar Cave, New Mexico	Upper P3	
EQ24	DP 2592	Cedral, Mexico	Lower m1r	
EQ25	DP 2704	Cedral, Mexico	Lower m2l	

EQ26	UTEP 22-1538	Dry Cave, New Mexico	Lower p4r	
EQ27	KU 32785	Natural Trap Cave, WY	Upper P3r	
EQ28	DP 3843	Cedral, Mexico	Upper M1l	
EQ29*/EQ29-2* (repeat)	DhPg-8 2993.1	Wally's Beach, Alberta	Upper DP4r	KX137133
Q30* (ind. replicated)	LACM 192/156481	San Josecito Cave, Mexico	Upper P2l	KX137134 KX137135
EQ31	UTEP 120-41	Big Manhole Cave, NM	Lower p3l	
EQ32	DP 3863	Cedral, Mexico	Upper P3l	
EQ33	KU 35118	Natural Trap Cave, WY	Lower p3/p4l	
EQ34	DP 3889	Cedral, Mexico	Lower m3r	
EQ35	LACM 192/156482	San Josecito Cave, Mexico	Upper M1	
EQ36	UTEP 22-1664	Dry Cave, New Mexico	Lower m1/m2l	
EQ37	TMM 41228-3821	Dark Canyon Cave, NM	Lower p3r	
EQ38*	MgVo-1 H7(W)-3-36	Bluefish Cave 1, Yukon	Upper P3r	KX137136
EQ39*/EQ39-2* (repeat)	MgVo-1 J8-1-147	Bluefish Cave 1, Yukon	Lower p3l	KX137137
EQ41*	KU 42070	Natural Trap Cave, WY	Lower m3r	KX137138 KX137139
EQ42*	MgVo-2 J7-8-19	Bluefish Cave 2, Yukon	Lower p4l	KX137140
EQ43 (p2r)* / EQ43-2 (p3l)* (repeat) (ind. replicated)	DhPg-8 3437.2 (Horse 2)	Wally's Beach, Alberta	Lower p2r and p3l	KX137141
EQ44*	MgVo-1 G7(E1/2)-11-13	Bluefish Cave 1, Yukon	Upper P3l	KX137142
EQ45*	MgVo-3 85-89	Bluefish Cave 3, Yukon	Upper P3l	KX137143
EQ46	TMM 937-253	Blackwater Draw, NM	Upper P4l	
EQ47*	MgVo-1 T3-21-86	Bluefish Cave 1, Yukon	Lower m2r	KX137144
EQ48*	MgVo-2 I6-E-5	Bluefish Cave 2, Yukon	Lower p4r	KX137145
EQ49	TMM 937-48	Blackwater Draw, NM	Lower p3/p4r	
EQ50*/EQ50-2* (repeat)	MgVo-1 L8(N)-4-7	Bluefish Cave 1, Yukon	Lower p3r	KX137146

EQ51* (ind. replicated)	MgVo-2 B3-3-16	Bluefish Cave 2, Yukon	Lower m2r	KX137147
EQ52	TMM 937-947	Blackwater Draw, NM	Lower m1r	
EQ53*/EQ53-2* (repeat)	MgVo-3 85-90 p3	Bluefish Cave 3, Yukon	Lower p3r	KX137148

Table E. Primers used to amplify a 621 bp fragment of the HVR I, mitochondrial control region. Position numbers are given according to the location of the first nucleotide of the primer over the complete mtDNA sequence of *Equus ferus caballus* (GenBank accession number JN398377).

Primer Name	Sequence	Description
EQL1-15425F	ACCATCAACACCCAAAGC	Horse, mtDNA HVR1, Forward primer
EQH1-15596R	TTAATGCACGAYGTACATAGG	Horse, mtDNA HVR1, Reverse primer
EQ-15496F	ACCCTCATGTRCYATGTCAGTA	Horse, mtDNA HVR1, Forward primer
EQ-15628R	TGTACATGCTTATTATTCATGGG	Horse, mtDNA HVR1, Reverse primer
EQ-15575F	GCCTATGTACRTCGTGCATT	Horse, mtDNA HVR1, Forward primer
EQ-15708R	TGTTGRCTGGAAATGATTTG	Horse, mtDNA HVR1, Reverse primer
EQ-15668_2F	TCGTGCATACCCCATYCAAG	Horse, mtDNA HVR1, Forward primer
EQ-15852R	GAACCAGATGCCAGGTATAGTTTC	Horse, mtDNA HVR1, Reverse primer
EQ-15782F	TCCCAATCCTCGCTCCG	Horse, mtDNA HVR1, Forward primer
EQ-15945R	TGTGAGCATGGGCTGATTAGTC	Horse, mtDNA HVR1, Reverse primer
EQ-15889_2F	CTTTCCCCTTAAATAAGACATCTCG	Horse, mtDNA HVR1, Forward primer
EQ-16018R	CTTTGACGGCCATAGCTGAGT	Horse, mtDNA HVR1, Reverse primer
EQ-15948F	TAACTGTGRTTTCATGCATTTGG	Horse, mtDNA HVR1, Forward primer
EQ-16085R	GGTTGCTGATGCGGAGTAATAA	Horse, mtDNA HVR1, Reverse primer
EQ-15799R	CCGGAGCGAGGATTGGG	Horse, mtDNA HVR1, Reverse primer

Table F. Equid sequences compiled from various publications [1–5] that were used in the Bayesian phylogenetic analysis of ancient mtDNA. Hap. = Haplogroup.

	GenBank #	Specimen	Provenance	Age (yr RCBP)
1	JN398377	*Equus ferus caballus* Chincoteague Pony; Hap. A	North America	Recent
2	JN398380	*E. f. caballus* Arabian; Hap. A	Middle East	Recent
3	JN398386	*E. f. caballus* Westphalian; Hap. B	Europe Centre	Recent
4	JN398387	*E. f. caballus* Maremmano; Hap. B	Europe South	Recent
5	JN398393	*E. f. caballus* Akhal-Teke; Hap. C	Asia Centre	Recent
6	JN398395	*E. f. caballus* Unspecified Iranian Breed; Hap. C	Middle East	Recent
7	JN398398	*E. f. caballus* Norwegian Fjord; Hap. D	Europe North	Recent
8	JN398400	*E. f. caballus* Icelandic Horse; Hap. D	Europe North	Recent
9	JN398401	*E. f. caballus* Maremmano; Hap. E	Europe South	Recent
10	JN398402	*E. f. przewalskii*; Hap. F	Asia Centre	Recent
11	JN398403	*E. f. przewalskii*; Hap. F	Asia Centre	Recent
12	JN398407	*E. f. caballus* Giara Horse; Hap. G	Sardinia; Europe South	Recent
13	JN398412	*E. f. caballus* Arabian; Hap. G	Middle East	Recent
14	JN398413	*E. f. caballus* Maremmano; Hap. H	Europe South	Recent
15	JN398416	*E. f. caballus* Caspian Pony; Hap. I	Middle East	Recent
16	JN398417	*E. f. caballus* Trakhener; Hap. I	Europe North	Recent
17	JN398418	*E. f. caballus* Maremmano; Hap. J	Europe South	Recent
18	JN398419	*E. f. caballus* Unspecified Iranian Breed; Hap. J	Middle East	Recent
19	JN398420	*E. f. caballus* Belgian Draft; Hap. K	Europe Centre	Recent
20	JN398421	*E. f. caballus* American Paint Horse; Hap. L	North America	Recent
21	JN398430	*E. f. caballus* Andalusian; Hap. L	Europe South	Recent

22	JN398436	*E. f. caballus* Caspian Pony; Hap. M	Middle East	Recent
23	JN398439	*E. f. caballus* Clydesdale; Hap. M	Europe North	Recent
24	JN398441	*E. ferus caballus* Saddlebred; Hap. N	North America	Recent
25	JN398442	*E. f. caballus* Exmoor Pony; Hap. N	Europe North	Recent
26	JN398445	*E. f. caballus* Unspecified Iranian Breed; Hap. O	Middle East	Recent
27	JN398446	*E. f. caballus* Unspecified Iranian Breed; Hap. P	Middle East	Recent
28	JN398448	*E. f. caballus* Arabian; Hap. P	Middle East	Recent
29	JN398450	*E. f. caballus* Akhal-Teke; Hap. Q	Asia Centre	Recent
30	JN398452	*E. f. caballus* Akhal-Teke; Hap. Q	Asia Centre	Recent
31	JN398456	*E. f. caballus* Maremmano; Hap. R	Europe South	Recent
32	JN398457	*E. f. caballus* Unspecified Iranian Breed; Hap. R	Middle East	Recent
33	NC_001788	*Equus asinus*	Europe North?	Recent
34	AP012271	*Equus africanus somaliensis*		Recent
35	AP012269	*E. f. przewalskii*	Asia Centre	Recent
36	HQ439484	*E. f. przewalskii*	Asia Centre	Recent
37	AF072994	*E. f. przewalskii*	Asia Centre	Recent
38	JX312726	JW328	Mineral Hill Cave, Nevada	
39	JX312727	YG 401.268[1]	Upper Quartz Creek, Yukon	
40	SRA082086*	TC21/YG148.20[1]	Thistle Creek, Yukon	560,000 – 780,000
41	SRA082086*	CGG10022[2]	Taymyr Peninsula, Siberia	38,565 ± 602
42	DQ007558	PET09[3]	Petersfels, Germany	12,545 ± 50
43	DQ007556	SMNS[4]	Hohlefels, Germany	12,550 ± 60
44	DQ007591	Vog IV-6671[5]	Vogelherd IV, Germany	13,845 ± 50
45	DQ007590	SMNS 8010[4]	Bockstein, Germany	47,100 ± 1000
46	DQ007572	EK 253/557[6]	Ignatievskaya, Ural Mts.	

47	DQ007571	EK 253/556[6]	Ignatievskaya, Ural Mts.	2,218 ± 34
48	JN570954	EK 994/217[6]	Sur'ya 5, Ural Mts.	
49	DQ007573	IEM 202-279[7]	Bol. Lyakhovsky Island, N-E Siberia	2,220 ± 50
50	DQ007605	IEM 202-128[7]	Bol. Lyakhovsky Island, N-E Siberia	20,100 ± 170
51	DQ007574	IEM 202-847[7]	Bol. Lyakhovsky Island, N-E Siberia	28,800 ± 1,100
52	DQ007575	IEM202-244[7]	Bol. Lyakhovsky Island, N-E Siberia	34,800 ± 1,000
53	DQ007553	PIN 3659-6[8]	Ulakhan-Sullar, Adycha R., Yana Basin, N-E Siberia	53,100 ± 1,700
54	DQ007576	PIN 3659-1[8]	Ulakhan-Sullar, Adycha R., Yana Basin, N-E Siberia	
55	DQ007577	IEM203-42[7]	Lena R. Delta, N-E Siberia	31,220 ± 180
56	DQ007578	IEM 205-6[7]	Bykovsky Peninsula, Lena Delta, N-E Siberia	
57	DQ007579	PIN 3658-121[8]	Alyoshkina, Kolyma R., N-E Siberia	
58	DQ007580	IEM 207-30[7]	Lena R. Delta, N-E Siberia	
59	DQ007581	PIN 169-43[8]	Kolyma R. lower course, N-E Siberia	
60	DQ007582	PIN 3751-151[8]	Yana R. Lower course, N-E Siberia	
61	DQ007583	PIN 3100-421[8]	Chukochya R., Kolyma Lowland, N-E Siberia	
62	DQ007552	IEM 200-483[7]	Bykovsky Peninsula, Lena Delta, N-E Siberia	27,500 ± 400
63	DQ007584	UA-97-061-221[9]	Castle River, Alaska	15,090
64	DQ007554	AA26819[10]	Ester Creek, Alaska	12,510 ± 130
65	DQ007585	CMN MgVo-3-85-60[11]	Bluefish Cave 3, Yukon	
66	DQ007557	CMN 49368[11]	Old Crow Loc. 22, Yukon	43,900 ± 180
67	DQ007586	YG110.111[1]	Irish Gulch, Yukon	
68	DQ007587	YG150.82[1]	Thistle Creek, Yukon	
69	DQ007559	P94.1.415[12]	Pit 48, Edmonton, Alberta	
70	DQ007594	P89.21.1[12]	Grande Prairie, Alberta	11,200 ± 90
71	DQ007588	KU42625[13]	Natural Trap Cave, Wyoming	
72	DQ007589	KU51467[13]	Natural Trap Cave, Wyoming	

73	DQ007569	KU62158[13]	Natural Trap Cave, Wyoming	
74	DQ007570	LACM 109/150807[14]	Gypsum Cave, Nevada	13,070 ± 55
75	DQ007567	YG130.3[1]	Quartz Creek, Yukon	46,600 ± 1000
76	DQ007568	YG109.7[1]	Quartz Creek, Yukon	>47,000
77	DQ007604	HS23[15]	Tonghe Bridge, Harbin, China	
78	DQ007601	YT03/280[1]	Thistle Creek, Yukon	
79	DQ007603	YT03/286[1]	Thistle Creek, Yukon	
80	DQ007602	YT03/279[1]	Thistle Creek, Yukon	
81	DQ007593	P97.12.9[12]	Cloverbar Sand and Gravel Pit, Edmonton, Alberta	
82	DQ007592	P98.6.8[12]	Cloverbar Sand and Gravel Pit, Edmonton, Alberta	
83	DQ007597	P96.2.33[12]	Pit 48, Edmonton, Alberta	
84	DQ007600	P96.9.18[12]	Twin Bridges Gravel Pit 80, Edmonton, Alberta	
85	DQ007595	P88.19.3[12]	Apex Evergreen Pit, Edmonton, Alberta	
86	DQ007598	P95.1.12[12]	Cloverbar Sand and Gravel Pit, Edmonton, Alberta	
87	DQ007599	P94.2.1[12]	Twin Bridges Gravel Pit 80, Edmonton, Alberta	
88	DQ007596	P95.2.29[12]	Apex Evergreen Pit, Edmonton, Alberta	
89	EU030680	INCUAPA uncatalogued[16]	Inti Huasi, San Luis province, Argentina	
90	EU030682	MLP 44 XII 29 23[17]	Arroyo Tapalqué (Olavarría), Buenos Aires province, Argentina	
91	AF326676	SK1[18]	Skedemosse in Öland, Sweden	200 – 500 AD
92	AF326677	SK2[18]	Skedemosse in Öland, Sweden	200 – 500 AD
93	AF326678	VenA9[18]	Vendel grave 9, Uppland, Sweden	Viking Period
94	AF326679	HALLA[18]	Broa i Hall in Gotland, Sweden	Late Viking Period
95	AF326665	*E. f. caballus* EC8A	*E. f. caballus*, Hap. A	Recent
96	AF326668	FAM 4695[10]	Fairbanks area, Alaska	12,000 – 28,000
97	AF326669	FAM 4305[10]	Fairbanks area, Alaska	12,000 – 28,000

98	AF326670	FAM 6735[10]	Fairbanks area, Alaska	12,000 – 28,000
99	AF326671	FAM 6206[10]	Fairbanks area, Alaska	12,000 – 28,000
100	AF326672	FAM 133514[10]	Fairbanks area, Alaska	12,000 – 28,000
101	AF326673	FAM 6300[10]	Fairbanks area, Alaska	12,000 – 28,000
102	AF326674	FAM 6171[10]	Fairbanks area, Alaska	12,000 – 28,000
103	AF326675	FAM 6282[10]	Fairbanks area, Alaska	12,000 – 28,000

1, YG = Yukon Department of Tourism and Culture, Whitehorse, Canada; 2, CGG = Centre for GeoGenetics, Copenhagen, Denmark; 3, PET = Landesdenkmalamt Baden-Württemberg, Konstanz, Germany; 4, SMNS = Stuttgart State Museum of Natural History, Stuttgart, Germany; 5, Vog = Institute of Pre- and Protohistory, University of Tübingen, Germany; 6, EK = Institute for Plant and Animal Ecology, Ekaterinburg, Russia; 7, IEM = Institute of Ecology and Evolution Russian Academy of Sciences, Moscow, Russia; 8, PIN = Paleontological Institute, Russian Academy of Sciences, Moscow, Russia; 9, UA = University of Alaska, Fairbanks, USA; 10, AA = Division of Paleontology, American Museum of Natural History, New York, USA; 11, CMN = Canadian Museum of Nature, Ottawa, Canada; 12, P = Royal Alberta Museum, Edmonton, Canada; 13, KU = Biodiversity Institute and Natural History Museum, University of Kansas, Lawrence, USA; 14, LACM = Natural History Museum of Los Angeles County, Los Angeles, USA; 15, HS = X. Gao, Harbin (China) private collection; 16, INCUAPA = Universidad Nacional del Centro de la provincia de Buenos Aires, Olavarría, Argentina; 17, MLP = Museo de La Plata, Buenos Aires, Argentina; 18, Swedish History Museum, Stockholm, Sweden.
* Sequence Read Archive.

References

1. Weinstock J, Willerslev E, Sher A, Tong W, Ho SYW, Rubenstein D, et al. Evolution, systematics, and phylogeography of Pleistocene horses in the New World: A molecular perspective. PLoS Biol. 2005; 3:e241.

2. Orlando L, Male D, Alberdi MT, Prado JL, Prieto A, Cooper A, et al. Ancient DNA clarifies the evolutionary history of American late Pleistocene equids. J Mol Evol. 2008; 66:533-538.

3. Achilli A, Olivieri A, Soares P, Lancioni H, Kashani BH, Perego UA, et al. Mitochondrial genomes from modern horses reveal the major haplogroups that underwent domestication. Proc Natl Acad Sci U S A. 2012; 109:2449-2454.

4. Vilstrup JT, Seguin-Orlando A, Stiller M, Ginolhac A, Raghavan M, Nielsen SCA, et al. Mitochondrial phylogenomics of modern and ancient equids. PLoS One. 2013; 8:e55950.

5. Orlando L, Ginolhac A, Zhang G, Froese D, Albrechtsen A, Stiller M, et al. Recalibrating *Equus* evolution using the genome sequence of an early Middle Pleistocene horse. Nature. 2013; 499:74-78.
